# The Impact of Oxygen Surface Coverage and Carbidic Carbon on the Activity and Selectivity of Two-Dimensional Molybdenum Carbide (2D-Mo_2_C) in Fischer–Tropsch Synthesis

**DOI:** 10.1021/acscatal.3c03956

**Published:** 2024-01-19

**Authors:** Evgenia Kountoupi, Alan J. Barrios, Zixuan Chen, Christoph R. Müller, Vitaly V. Ordomsky, Aleix Comas-Vives, Alexey Fedorov

**Affiliations:** † Department of Mechanical and Process Engineering, ETH Zürich, Zürich CH-8092, Switzerland; ‡ University of Lille, CNRS, Centrale Lille, University of Artois, UMR 8181 − UCCS − Unité de Catalyse et Chimie du Solide, Lille 59000, France; § Laboratory for Chemical Technology, Department of Materials, Textiles and Chemical Engineering, Ghent University, Ghent B-9052, Belgium; ∥ Institute of Materials Chemistry, Technische Universität Wien, Vienna 1060, Austria; ⊥ Departament de Química, Universitat Autònoma de Barcelona, Cerdanyola del Vallès 08193, Catalonia, Spain

**Keywords:** carbide catalysts, defunctionalization of MXenes, Fischer−Tropsch synthesis, two-dimensional (2D) materials, oxygen coverage, molybdenum carbide, DFT calculations

## Abstract

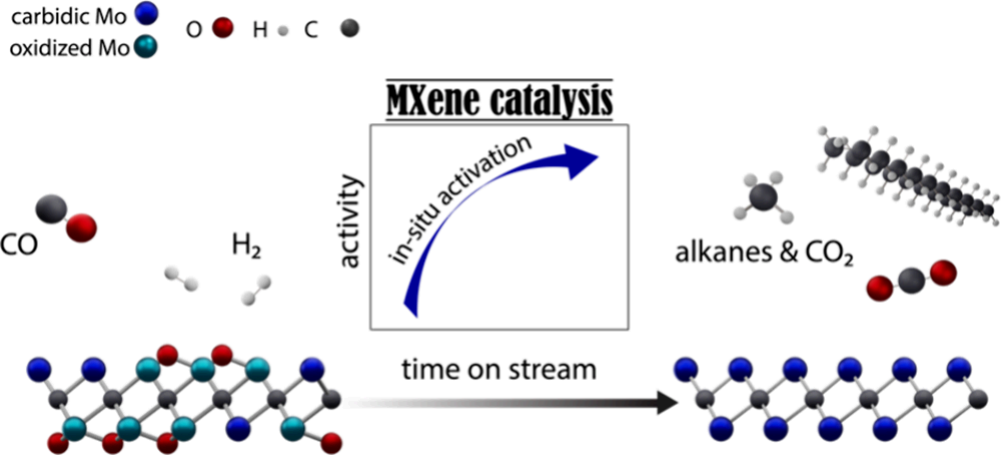

Transformations of oxygenates (CO_2_, CO, H_2_O, etc.) via Mo_2_C-based catalysts are facilitated by the high oxophilicity of the material; however, this can lead to the formation of oxycarbides and complicate the identification of the (most) active catalyst state and active sites. In this context, the two-dimensional (2D) MXene molybdenum carbide Mo_2_C*T*_*x*_ (*T*_*x*_ are passivating surface groups) contains only surface Mo sites and is therefore a highly suitable model catalyst for structure–activity studies. Here, we report that the catalytic activity of Mo_2_C*T*_*x*_ in Fischer–Tropsch (FT) synthesis increases with a decreasing coverage of surface passivating groups (mostly O*). The *in situ* removal of *T*_*x*_ species and its consequence on CO conversion is highlighted by the observation of a very pronounced activation of Mo_2_C*T*_*x*_ (pretreated in H_2_ at 400 °C) under FT conditions. This activation process is ascribed to the *in situ* reductive defunctionalization of *T*_*x*_ groups reaching a catalyst state that is close to 2D-Mo_2_C (i.e., a material containing no passivating surface groups). Under steady-state FT conditions, 2D-Mo_2_C yields higher hydrocarbons (C_5+_ alkanes) with 55% selectivity. Alkanes up to the kerosine range form, with value of α = 0.87, which is ca. twice higher than the α value reported for 3D-Mo_2_C catalysts. The steady-state productivity of 2D-Mo_2_C to C_5+_ hydrocarbons is ca. 2 orders of magnitude higher relative to a reference β-Μo_2_C catalyst that shows no *in situ* activation under identical FT conditions. The passivating *T*_*x*_ groups of Mo_2_C*T*_*x*_ can be reductively defunctionalized also by using a higher H_2_ pretreatment temperature of 500 °C. Yet, this approach leads to a removal of carbidic carbon (as methane), resulting in a 2D-Mo_2_C_1–*x*_ catalyst that converts CO to CH_4_ with 61% selectivity in preference to C_5+_ hydrocarbons that are formed with only 2% selectivity. Density functional theory (DFT) results attribute the observed selectivity of 2D-Mo_2_C to C_5+_ alkanes to a higher energy barrier for the hydrogenation of surface alkyl species relative to the energy barriers for C–C coupling. The removal of O* is the rate-determining step in the FT reaction over 2D-Mo_2_C, and O* is favorably removed in the form of CO_2_ relative to H_2_O, consistent with the observation of a high CO_2_ selectivity (ca. 50%). The absence of other carbon oxygenates is explained by the energetic favoring of the direct over the hydrogen-assisted dissociative adsorption of CO.

## Introduction

The Fischer–Tropsch (FT) process has been utilized for nearly a century to hydrogenate carbon monoxide, typically derived from feedstocks such as coal, natural gas, and more recently, biomass, into chemicals and fuels.^1,2^ This exothermic reaction proceeds according to eq 1.^3^
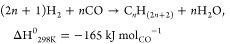
1

Product selectivity can be tuned through the choice of catalyst, and it varies between *n*-alkanes, olefins, and oxygenates (typically, alcohols).^4^ The industrial FT catalysts are usually based on transition metals such as Fe and Co.^5−9^ In the past decades, the development of alternative FT catalysts aimed at tailoring the chain-length distribution of the products, for instance, by narrowing the broad Anderson–Schulz–Flory distribution to the desired fuel range (C_10_–C_20_ hydrocarbons for diesel fuel).^10,11^ In this context, early transition metal carbides (TMCs), in particular Mo carbides, have been explored as FT catalysts.^12^ Unsupported Mo carbides (cubic α-MoC_1–*x*_ and orthorhombic β-Μo_2_C phases) yield mainly methane, CO_2_, lower alkanes, and alkenes as FT products, with a chain probability growth coefficient (α) equal to 0.3–0.4.^13−15^ Using Μo_2_C, the formation of alcohols has been reported as well^16^ and approaches to increase the selectivity of Mo carbides to C_2+_ alcohols include their promotion with alkali metals such as potassium,^17^ or dispersing Mo carbide on a support (e.g., Al_2_O_3_, TiO_2_).^18−20^

Turning to the catalytic pathways of the FT process, the main mechanisms proposed are the carbide mechanism, the CO insertion mechanism, and the hydroxycarbene mechanism, each involving initiation, propagation, and chain termination steps.^2^ Briefly, the carbide mechanism, proposed by Fischer and Tropsch,^21^ proceeds via the dissociative adsorption of CO to C* and O* that occurs simultaneously with the dissociation of H_2_ to H* species and the subsequent hydrogenation of C* to CH_x_* (*x* = 1–3) species; the latter are involved in chain growth (the C–C coupling step).^22^ The dissociation of CO* may occur directly or may be hydrogen-assisted (molecular or H*).^23^ The chain growth step can involve methylene (CH_2_*),^24^ methylidyne (CH*),^25−28^ or coupling between C* and CH* species.^29^ In the CO insertion mechanism, CO inserts into an M–H bond (initiation) followed by the hydrogenation of the formyl (HCO*) species into CH_2_O* species that are hydrogenated to CH_3_* species and H_2_O; further CO insertion into a metal–alkyl bond and hydrogenation is repeated until chain growth is terminated.^30^ In contrast, the hydroxycarbene mechanism is linked to the formation of oxygenates and it includes a coupling reaction between two hydroxycarbene (RCOH*, R = H, alkyl) intermediates formed via the hydrogenation of adsorbed CO.^31^

All three main FT mechanisms have been proposed to proceed on Μo_2_C. In particular, it has been suggested that Mo_2_C catalyzes FT via a direct dissociation of CO into O* and C* species, i.e., a carbidic mechanism.^32^ Specifically, CO adsorption and H_2_ temperature-programmed experiments have been used to confirm the direct dissociation of CO on a Mo_2_C surface.^14^ However, the O* adsorbates formed can inhibit a further dissociation of CO and thereby deactivate the catalyst.^33^ The involvement of an H-assisted pathway for the dissociation of CO has been suggested as well, in particular as a means to avoid the formation of a too-strongly bound O* species that reduce the catalytic turnover.^34^ Moreover, a computational study suggested a CO insertion mechanism with a coupling between the CH_*x*_ and CH_*y*_O species to proceed on Mo_2_C.^35^ Lastly, the hydroxycarbene mechanism has been suggested for Mo_2_C to account for the formation of alcohols.^14^

Oxycarbidic Mo_2_C_*x*_O_*y*_ species/phases can form *in situ* from Mo_2_C via its reaction with oxygenates, and they have been proposed to be the catalytically activity centers for the dry reforming of methane (DRM) or hydrodeoxygenation reactions.^36,37^ Depending on the chemical potential of the gas phase, Mo-terminated surfaces of Mo_2_C can feature different coverages of O* adsorbates that can block adsorption sites and modify adsorption energies of reaction intermediates, which in turn influences the overall catalytic activity and product selectivity.^38,39^ While it is generally believed that higher O* coverages are associated with a lower FT activity,^32,40^ it is yet unclear if there is an optimum (low) O* coverage to yield the highest FT activity on Mo_2_C; furthermore, the dependence of product selectivity on O* coverage of Mo_2_C is also understudied.

One strategy to improve our understanding of the catalytic activity of Mo_2_C relies on the use of model catalysts with well-defined structures and surfaces, which facilitates their spectroscopic characterization and bridges the gap to computational models.^41−44^ In this context, well-defined two-dimensional (2D) carbides of the MXene family,^45−48^ in particular Mo_2_C*T*_*x*_ (*T*_*x*_ are O, OH, and F surface termination groups), can serve as model catalysts owing to the controllable reductive defunctionalization of Mo_2_C*T*_*x*_ (either partial or complete),^49,50^ in combination with a thermal stability of up to ca. 550–600 °C (for multilayer Mo_2_C*T*_*x*_ with a nanoplatelet morphology),^49,50^ and a single (0001) basal surface structure.^46^ Specifically, Mo_2_C*T*_*x*_-derived catalysts proved useful in deciphering the electronic state of Mo atoms (average oxidation state of Mo that is linked to the O* coverage) under (reverse) water gas shift (R)WGS conditions. For instance, under RWGS conditions, a Mo_2_C*T*_*x*_-derived catalyst free from *T*_*x*_ groups evolved toward a structure with a relatively low but measurable O* coverage.^50^ In contrast, under WGS conditions, the same catalyst evolved to a full O* coverage, i.e., similar to the state of Mo in the parent Mo_2_C*T*_*x*_ (ca. +4.5 average Mo oxidation state), and the catalytic activity declined with increasing surface functionalization by O* species.^49,50^

This work aims to understand the relation between the composition of Mo_2_C*T*_*x*_-derived catalysts, in particular their surface oxygen coverage and carbidic carbon content, and activity and selectivity in the FT process. We show that Mo_2_C*T*_*x*_ pretreated at 500 °C in undiluted H_2_ (i.e., Mo_2_C*T*_*x*–500_), a material with Mo atoms only in a carbidic state (Mo^2+^), is a notably more active FT catalyst than Mo_2_C*T*_*x*_ pretreated at 400 °C (i.e., Mo_2_C*T*_*x*–400_), which has both Mo^2+^ and Mo^4+^ states in a ratio of ca. 2:3. Interestingly, the activity of Mo_2_C*T*_*x*–400_ increases appreciably with time on stream (TOS), which is explained by a decreasing *T*_*x*_ coverage with TOS via an *in situ* reduction of Mo^4+^ oxycarbidic states to the Mo^2+^ carbidic state. Interestingly, while both the *in situ* activated Mo_2_C*T*_*x*–400_ and Mo_2_C*T*_*x*–500_ display comparable steady-state CO conversion rates, a substantially different product selectivity is observed between these two catalysts at ca. 90% CO conversion. Specifically, while the *in situ* activated Mo_2_C*T*_*x*–400_ produces predominantly C_5+_ alkanes, Mo_2_C*T*_*x*–500_ is selective to methane (55 and 61%, respectively). This distinct selectivity is explained by differences in the structure (and the active sites) and in particular the substoichiometric carbidic carbon content in Mo_2_C*T*_*x*–500_. The latter material is more accurately described as 2D-Mo_2_C_1–*x*_ (rather than 2D-Mo_2_C), with an atomic ratio of Mo to C^carb^ of 2.8:1, while *in situ* activated Mo_2_C*T*_*x*–400_ features an atomic ratio of Mo to C^carb^ of 1.9:1, which is close to that in the starting Mo_2_C*T*_*x*_, i.e., (2.0 ± 0.2):1. In addition, the morphology of the catalyst is found to impact the chain probability growth coefficient α that is approximately twice higher for the 2D-Mo_2_C catalyst relative to reported values for 3D-Mo_2_C (0.87 and ca. 0.3–0.4, respectively), which might be due to a confinement effect (chain growth in the interlayer space between the MXene sheets). The amount of C_5+_ hydrocarbons produced per catalyst mass is substantially larger (by ca. 2 orders of magnitude) for 2D-Mo_2_C relative to a reference β-Μo_2_C catalyst (exposed to identical pretreatment conditions), highlighting the high (yet understudied) potential of MXenes for thermocatalytic applications. Density functional theory (DFT) calculations identify a low barrier for the direct CO dissociation, suggesting a carbide mechanism, in which chain growth preferentially occurs via the coupling between CH* and C* species. The DFT energy profile corroborates the experimentally observed selectivity patterns, including the production of higher alkanes and CO_2_ in the absence of oxygenates.

## Methods

### Synthesis and Characterization

Mo_2_Ga_2_C was synthesized from β-Mo_2_C and metallic Ga following a reported method.^51^ The subsequent removal of Ga to yield the multilayered Mo_2_C*T*_*x*_ was performed by stirring Mo_2_Ga_2_C with 14 M HF at 140 °C for 7 days.^49,50,52,53^ The activated catalysts denoted Mo_2_C*T*_*x*–400_ and Mo_2_C*T*_*x*–500_ were prepared by treating the as synthesized Mo_2_C*T*_*x*_ (ca. 40 mg) in a vertical quartz reactor (i.d. 12 mm) with a flow of undiluted H_2_ (20 mL min^–1^, 1 bar) at 400 and 500 °C, respectively (heating ramp was 5 °C min^–1^) for 2 h.^50^ While we have reported previously that Mo_2_C*T*_*x*–500_ corresponds to 2D-Mo_2_C, which is a multilayered material with a morphology of Mo_2_C*T*_*x*_, but with the absence of *T*_*x*_ groups,^50^ in what follows we refine this description and demonstrate that Mo_2_C*T*_*x*–500_ is more appropriately represented as 2D-Mo_2_C_1–*x*_. The activated materials were cooled down under N_2_ flow (20 mL min^–1^) and transferred to a glovebox (H_2_O and O_2_ < 1 ppm) without exposure to air. The materials denoted as β-Mo_2_C_(400)_ and β-Mo_2_C_(500)_ were prepared from β-Mo_2_C in the above-described conditions, at 400 and 500 °C, respectively. For the catalytic FT tests, the activated materials were prepared *in situ*, before switching to the reaction conditions, as described below. Additional details on the synthesis of materials and details on the powder X-ray diffraction (XRD), X-ray photoelectron spectroscopy (XPS), Raman spectroscopy, and CO chemisorption methods are provided in the Supporting Information.

### Catalytic Testing

The catalytic performance of β-Mo_2_C and Mo_2_C*T*_*x*_, after H_2_ pretreatment, was evaluated in two different reactors. The first reactor, made of stainless-steel SS316 with an internal diameter and length of 2 and 150 mm, respectively, allowed the quantification of the liquid products. A second reactor, made of Hastelloy X, with an internal diameter and length of 9.1 and 305 mm, respectively, was used in experiments performed to recover and characterize the activated catalysts without air exposure. In a typical catalytic experiment in the SS316 reactor, β-Mo_2_C or Mo_2_C*T*_*x*_ (100 mg) was first pretreated in undiluted H_2_ (20 mL min^–1^) at, respectively, 400 or 500 °C (ramping rate 1 C min^–1^) for 2 h; subsequently, the temperature decreased to 180 °C, the gas atmosphere was switched to syngas (H_2_:CO = 2:1), and the pressure was set to 25 bar with the subsequent increase of the reaction temperature to 330 °C. N_2_ contained in the gas bottle of CO (5%) served as an internal standard. In the material recovery experiments employing the Hastelloy X reactor, a N_2_ flow of 1 or 2 mL min^–1^ was used as an internal standard to calculate the CO conversion. Time zero of the TOS scale corresponds to the first GC point for which the concentration of the internal standard (N_2_) stabilized (i.e., after ca. 2 h of the start of the experiments in the 2 mm reactor and ca. 4.5 h for the experiments in the 9.1 mm reactor). The total flow rate was 8.5 or 9.5 mL min^–1^, yielding weight hourly space velocities (WHSV) of 5.1 or 5.7 L g_cat_^–1^ h^–1^ for the SS316 and Hastelloy X reactors, respectively. Gas chromatography (GC) analysis of the reagents and gaseous reaction products was performed using a Varian CP 3800 instrument equipped with a thermal conductivity detector (TCD) and a flame ionization detector (FID). Two columns were used for the analysis, a packed CTR 1 column connected to the TCD and an Rt-Q-PLOT capillary column connected to the FID. Heavier hydrocarbon products were collected and analyzed offline. ^1^H NMR analysis of the liquid fraction validated the absence of oxygenates (Figure S6). To analyze the heavier hydrocarbon products, ca. 90 mg of the heavy hydrocarbon fraction was dissolved in dichloromethane and subsequently analyzed using a SCION SQ-GCMS instrument. The Anderson–Schulz–Flory distribution was plotted for C_10_–C_23_ products to calculate the chain growth probability coefficient α. When the catalytic experiments were performed in a larger reactor, the activated catalysts were recovered in a glovebox for characterization and handled without exposure to air. In this reactor setup, the gaseous CO consumption was quantified with a PerkinElmer Clarus 580 GC equipped with a TCD.

### Computational Details

Periodic DFT calculations were performed with the Vienna Ab Initio Simulation Package (VASP).^54,55^ The reported energy values correspond to Gibbs energies at 330 °C and 25 bar. The theoretical model of the (0001) facet of 2D-Mo_2_C is shown in Figure S16 and has been previously reported.^56^ Further details of the DFT calculations are provided in the Supporting Information.

## Results

### Materials

**Figure 1:**
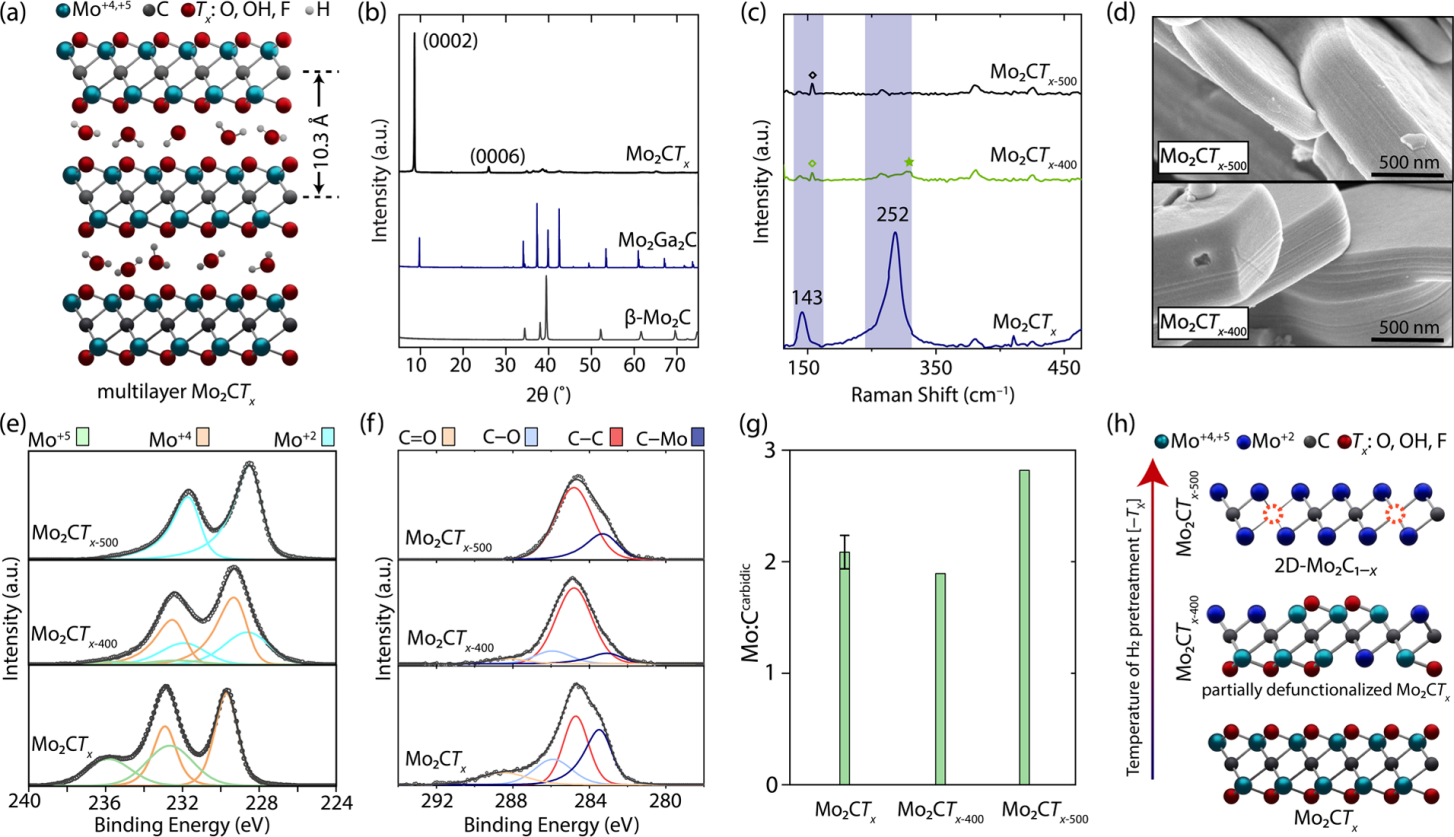
(a) Schematic representation and (b) XRD pattern of as-prepared multilayered Mo_2_C*T*_*x*_. (c) Raman spectra of Mo_2_C*T*_*x*_, Mo_2_C*T*_*x*–400_, and Mo_2_C*T*_*x*–500_. The diamond symbol denotes a peak from the quartz capillary. (d) SEM images of Mo_2_C*T*_*x*–400_ and Mo_2_C*T*_*x*–500_. (e) Mo 3d and (f) C 1s XPS spectra of Mo_2_C*T*_*x*_, Mo_2_C*T*_*x*–400_, and Mo_2_C*T*_*x*–500_. (g) Atomic ratio between molybdenum and carbidic carbon of Mo_2_C*T*_*x*_, Mo_2_C*T*_*x*–400_, and Mo_2_C*T*_*x*–500_. (h) Schematic representation of the reductive defunctionalization of Mo_2_C*T*_*x*_ via H_2_ pretreatment.

Multilayered nanoplatelets of Mo_2_C*T*_*x*_ shown schematically in Figure 1a were obtained by etching Ga from Mo_2_Ga_2_C with HF, following a published method.^49^ The XPS spectrum of Mo_2_C*T*_*x*_ features only a trace signal in the Ga 2p region, consistent with the successful removal of Ga (Figure S1). XRD of Mo_2_C*T*_*x*_ shows no reflections due to Mo_2_Ga_2_C but reveals a sharp characteristic low angle peak at 8.5°, owing to the (0002) planes of the stacked nanosheets in multilayered Mo_2_C*T*_*x*_ (Figure 1b).^49,53^

Our previous Mo K-edge X-ray absorption near edge structure (XANES) study has shown that while the edge energy of Mo_2_C*T*_*x*_ is found at 20011.2 eV, indicating an average ca. Mo^4+^ oxidation state in Mo_2_C*T*_*x*_, the energies for β-Mo_2_C and a material obtained after the pretreatment of Mo_2_C*T*_*x*_ in undiluted H_2_ at 500 °C for 2 h (Mo_2_C*T*_*x*–500_) are close, i.e., 20000.8 and 20001.4 eV, respectively.^50^ In addition, XPS analysis of Mo_2_C*T*_*x*–500_, performed under airtight conditions, revealed the presence of a single electronic state of Mo at a binding energy of 228.4 eV, assigned to the Mo^2+^ state. The layered structure of Mo_2_C*T*_*x*–500_ is evident from the presence of the (0002) reflection at 11.5° (XRD measurement performed in air). To conclude, our reported data showed that after pretreatment in undiluted H_2_ for 2 h, Mo_2_C*T*_*x*_ transforms into a material that is free from detectable amounts of surface termination groups.^50^ That being said, using 20% H_2_/N_2_ at 500 °C leads only to a partial defunctionalization of Mo_2_C*T*_*x*_.^49^

With these results in mind, we pretreated Mo_2_C*T*_*x*_ at 400 °C under a flow of undiluted H_2_ for 2 h (material denoted as Mo_2_C*T*_*x*–400_) and performed a Raman analysis to compare the results obtained to that of as-prepared Mo_2_C*T*_*x*_ and Mo_2_C*T*_*x*–500_. The Raman spectrum of as-prepared Mo_2_C*T*_*x*_, excited by a 780 nm laser, displays two characteristic bands centered at 143 and 252 cm^–1^ (Figure 1c and Figure S2). Raman bands at similar positions were also reported for Ti-based MXenes.^57^ Theoretical calculations of a 2D-Mo_2_CO_2_ model with O* groups occupying a 3-fold hollow site attributed the Raman vibrations at 123 and 236 cm^–1^ to in-plane (E_g_) and out-of-plane (A_1g_) vibrations of the O* groups, respectively.^58^ Our experimentally observed frequencies for Mo_2_C*T*_*x*_ are ca. 20 cm^–1^ higher than the calculated ones, possibly owing to the presence of oxo, hydroxy, and fluoro terminations in Mo_2_C*T*_*x*_ in the experimental system as identified by XPS analysis (Figure S3; the DFT model only considered oxo groups instead). Next, we assessed the evolution of the Raman bands at 143 and 252 cm^–1^ as a function of pretreatment temperature (spectra collected of materials kept in airtight capillaries). While the spectrum of Mo_2_C*T*_*x*–400_ features only a low-intensity A_1g_ band that is shifted to 264 cm^–1^, both E_g_ and A_1g_ vibrations are absent in Mo_2_C*T*_*x*–500_, in line with the complete surface defunctionalization in this material. According to scanning electron microscopy, both Mo_2_C*T*_*x*–400_ and Mo_2_C*T*_*x*–500_ feature a layered nanoplatelet morphology typical for initial Mo_2_C*T*_*x*_ (Figure 1d).^49,50,53^

Initial Mo_2_C*T*_*x*_ features a mixture of Mo^4+^ (55%) and Mo^5+^ (45%) states with the respective binding energies of 229.5 and 232.4 eV, owing to the oxidation of Mo sites by the *T*_*x*_ groups; note that there is no Mo^2+^ state due to carbidic Mo in initial (i.e., as-prepared) Mo_2_C*T*_*x*_ (Figure 1e and Table S7).^49,50^ In contrast, the two main electronic states of Mo found in Mo_2_C*T*_*x*–400_ are Mo^4+^ (57%) and Mo^2+^ (38%) with the respective binding energies of 229.1 and 228.3 eV, (Table S7, Figure 1e, and Figure S4). Finally, in Mo_2_C*T*_*x*–500_, Mo is exclusively in a Mo^2+^ state (carbidic Mo).^49,50^ The corresponding average oxidation states of Mo in Mo_2_C*T*_*x*_, Mo_2_C*T*_*x*–400_, and Mo_2_C*T*_*x*–500_ are ca. +4.5 (Mo^4+^/Mo^5+^ in ca. 1:1 ratio), +3.3 (Mo^4+^ and Mo^2+^ in ca. 3:2 ratio), and +2 (only carbidic Mo), respectively. Overall, the XPS data are consistent with the partially defunctionalized Mo sites in Mo_2_C*T*_*x*–400_.

Turning to the C 1s XPS region of Mo_2_C*T*_*x*_, in addition to adventitious carbon at a binding energy of 284.7 eV, peaks fitted with BE at 283.4, 285.9, and 288.5 eV are assigned to carbidic carbon (C–Mo), C–O, and C=O fragments, respectively (Figure 1f and Table S8). Importantly, initial Mo_2_C*T*_*x*_ displays an atomic ratio of molybdenum to carbidic carbon (Mo:C^carb^) of (2.0 ± 0.2):1 (Figure 1g; the error bar represents the standard deviation from the measurement of three independent batches of Mo_2_C*T*_*x*_). Mo_2_C*T*_*x*–400_ displays a Mo:C^carb^ ratio of 1.9:1, similar to that of initial Mo_2_C*T*_*x*_. In contrast, the Mo:C^carb^ ratio in Mo_2_C*T*_*x*–500_ is increased notably to 2.8:1. This result can be explained by the loss of carbidic carbon during the H_2_ pretreatment at 500 °C (in the form of methane, *vide infra*).

We have further verified inferences from the XPS study by performing a H_2_ temperature-programmed reduction (TPR) experiment using Mo_2_C*T*_*x*_ and following the off-gas by MS analysis (Figure S5). Species with a *m*/*z* ratio of 16 and 15 appear due to the ionization of methane. The signal of those species undergoes only a slight increase during the isothermal segment at 400 °C, and a notable rise is observed when the temperature is increased to 500 °C, consistent with the XPS results discussed above. Species with *m*/*z* 18 and 17 are predominantly due to the ionization of water; the latter is formed during the reductive defunctionalization of the *T*_*x*_ groups. During this experiment, we cofed N_2_ to H_2_ as an internal standard (2 mL min^–1^ of N_2_ flow added to 20 mL min^–1^ of H_2_). The observed stability of the *m*/*z* 28 signal during the entire TPR-MS experiment validates that the observed intensity changes of other signals can be associated with the reductive transformations of Mo_2_C*T*_*x*_.

A schematic representation visualizing the structural modification during the reductive defunctionalization of Mo_2_C*T*_*x*_ under undiluted H_2_ yielding Mo_2_C*T*_*x*–400_ and Mo_2_C*T*_*x*–500_ (i.e., 2D-Mo_2_C_1–*x*_) is shown in Figure 1h.

### *In Situ* Activation of Mo_2_C*T*_*x*–400_ under FT Conditions

**Figure 2:**
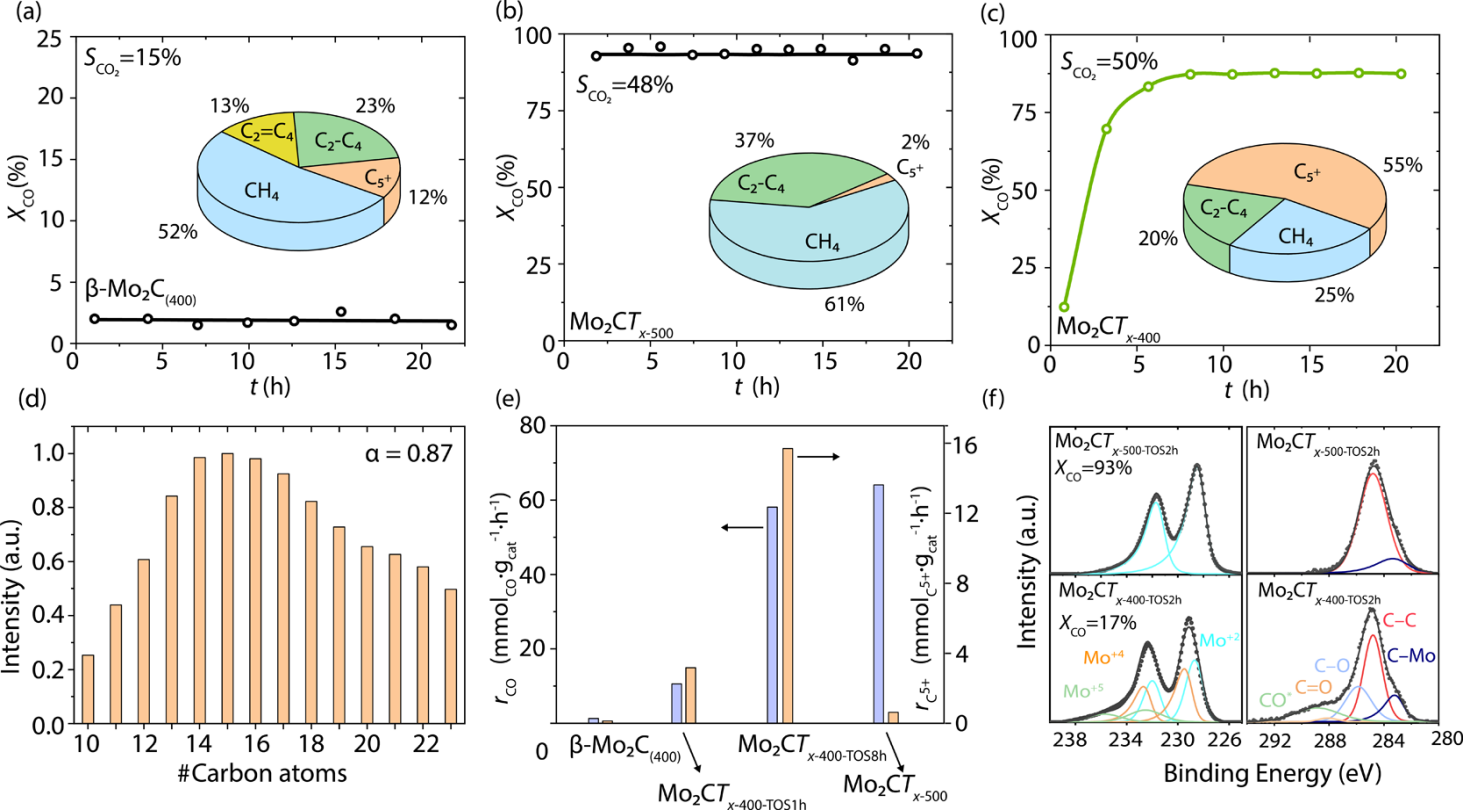
Conversion of CO with TOS using (a) β-Mo_2_C_(400)_, (b) Mo_2_C*T*_*x*–500_, and (c) Mo_2_C*T*_*x*–400_ in FT. Insets show the gas-phase product distribution for the steady-state activity that corresponds to *X*_CO_ = 2, 94, and 88%, respectively. (d) Liquid phase analysis (post reaction) for Mo_2_C*T*_*x*–400_. Catalytic tests were performed in a stainless-steel reactor with 2 mm internal diameter at 25 bar, 330 °C, with a CO:H_2_ ratio of 1:2 and a space velocity of 5.1 L·(g_cat_·h)^−1^. The carbon balance is close to 100% for most of GC points and exceeds 90% for all GC points. (e) Steady-state gravimetric rate of CO consumption and C_5+_ production for β-Mo_2_C_(400)_, Mo_2_C*T*_*x*–500_, and Mo_2_C*T*_*x*–400–TOS8h_ as well as the respective initial rates (i.e., before *in situ* activation) for Mo_2_C*T*_*x*–400–TOS1h_. (f) Mo 3d (left) and C 1s (right) XPS spectra of Mo_2_C*T*_*x*–400_ and Mo_2_C*T*_*x*–500_ after ca. 2 h TOS. Experiments designed to recover and characterize the activated catalyst were performed in a Hastelloy reactor with 9.1 mm internal diameter at 25 bar, 330 °C, with a CO:H_2_ ratio of 1:2 and a space velocity of 5.7 L·(g_cat_·h)^−1^.

Turning to the FT activity of the prepared materials, we first examined the performance of the reference β-Mo_2_C_(400)_ using a H_2_:CO ratio of 2:1, 330 °C, and 25 bar and a WHSV of 5.1 L·(g_cat_·h)^−1^. β-Mo_2_C_(400)_ shows a stable CO conversion of only ca. 2% (Figure 2a). At this very low conversion (that corresponds to a gravimetric CO consumption of 1.3 mmol CO g_cat_ h^–1^), the selectivity to CO_2_ is 15% and the partial selectivities (i.e., selectivity excluding CO_2_) to CH_4_, C_2_–C_4_ alkanes, C_2_–C_4_ alkenes, and C_5+_ alkanes are 52, 23, 13, and 12%, respectively (Figure 2a). No organic liquid fraction in amounts sufficient for analysis was produced.

In sharp contrast, Mo_2_C*T*_*x*–500_ displays, under identical testing conditions, an initial conversion of 94% and shows no further changes in CO conversion and product selectivity within the whole duration of the experiment (ca. 20 h TOS, Figure 2b and Table S1). The selectivity to CO_2_ on Mo_2_C*T*_*x*–500_ is 48%, while the partial selectivities to CH_4_, C_2_–C_4_ alkanes, and C_5+_ alkanes are 61, 37, and 2%, respectively. In contrast to β-Mo_2_C_(400)_, no significant amounts of olefins (i.e., >0.5%) are detected for Mo_2_C*T*_*x*–500_. As in the case of β-Mo_2_C_(400)_, no organic liquid fraction in amounts sufficient for analysis was produced during the catalytic test. Overall, Mo_2_C*T*_*x*–500_ is a poor FT catalyst that produces 19.9 mmol CH_4_ g_cat_ h^–1^ and merely 0.6 mmol C_5+_ g_cat_ h^–1^ (i.e., it is rather a methanation catalyst than a catalyst for FT).

Next, we assessed the FT activity of Mo_2_C*T*_*x*–400_ under identical conditions and observed a remarkable *in situ* activation of Mo_2_C*T*_*x*–400_ with TOS; that is, the initial CO conversion of 12% at ca. 1 h increased to 88% after 8 h of TOS and remained stable until the end of the experiment (ca. 20 h, Figure 2c). We denote the initial catalyst as Mo_2_C*T*_*x*–400–TOS1h_ and the catalyst that has undergone the *in situ* activation and reached the steady-state conditions as Mo_2_C*T*_*x*–400–TOS8h_. The selectivity to CO_2_ for Mo_2_C*T*_*x*–400–TOS8h_ is 50%, which parallels that of Mo_2_C*T*_*x*–500_. Yet, the partial selectivities to CH_4_, C_2_–C_4_ alkanes, and C_5+_ alkanes are 25, 20, and 55%, respectively (Figure 2c and Table S1, entry 4). The liquid fraction, accumulated throughout the experiment, consisted of water and higher alkanes, with an alkane distribution corresponding to a chain growth probability coefficient α = 0.87 (Figure 2d). This α value is ca. two times higher than the value reported for unsupported 3D molybdenum carbides (α-MoC_1–*x*_ and β-Mo_2_C)^14^ and is similar to Fe-based FT catalysts.^59^ Furthermore, no oxygenates (Figure S6) or waxes were formed on Mo_2_C*T*_*x*–400_. When evaluating the transient period of the experiment, it is observed that with increasing CO conversion, the selectivity toward CO_2_ increases at the expense of hydrocarbon selectivity (Figure S7 and Table S1, entries 2–4).

The gravimetric rate of CO consumption and that of C_5+_ production for β-Mo_2_C_(400)_, Mo_2_C*T*_*x*–500_, Mo_2_C*T*_*x*–400–TOS1h_, and Mo_2_C*T*_*x*–400–TOS8h_ are plotted in Figure 2e and presented in Table S2. More specifically, Mo_2_C*T*_*x*–400–TOS8h_ and Mo_2_C*T*_*x*–500_ convert CO with similar rates, i.e., 58 and 62 mmol CO g_cat_ h^–1^, at 88 and 94% CO conversion, respectively. These rates are ca. 7 and 45 times higher relative to Mo_2_C*T*_*x*–400–TOS1h_ and β-Mo_2_C_(400)_, at 16 and 2% CO conversion, respectively. Interestingly, the gravimetric formation rate to C_5+_ alkanes (15.7 mmol C_5+_ g_cat_ h^–1^) displayed by Mo_2_C*T*_*x*–400–TOS8h_ is ca. 25 times higher than that of Mo_2_C*T*_*x*–500_. In what follows, we will rationalize the *in situ* activation results by correlating the activities of Mo_2_C*T*_*x*–400_ and Mo_2_C*T*_*x*–500_ to the *ex situ* XPS analysis of the activated catalysts.

Next, we compared the XPS spectra of Mo_2_C*T*_*x*–400_ and Mo_2_C*T*_*x*–500_ to that of active Mo_2_C*T*_*x*–400–TOS2h_ and Mo_2_C*T*_*x*–500–TOS2h_. Here, Mo_2_C*T*_*x*–400_ and Mo_2_C*T*_*x*–500_ were tested in FT conditions (25 bar, 330 °C, CO:H_2_ ratio of 1:2 for 2 h), followed by their recovery and XPS analysis without exposure to air. Mo_2_C*T*_*x*–400_ displays an initial CO conversion of 13% that increases to 17% after 2 h TOS. We note that in this case, Mo_2_C*T*_*x*–400–TOS2h_ has not yet reached the steady state. In contrast, Mo_2_C*T*_*x*–500_ displays a stable CO conversion of 93%. The values of CO conversion displayed by Mo_2_C*T*_*x*–400–TOS2h_ and Mo_2_C*T*_*x*–500–TOS2h_ are close to those of Mo_2_C*T*_*x*–400_ before and after its *in situ* activation, respectively, of the experiment described in Figure 2c; in addition, both experiments with Mo_2_C*T*_*x*–500_ show similar steady-state conversions (94 and 93%) and no activation period (Figure 2b and Figure S11). Compared to the catalytic FT experiments, *in situ* activation of Mo_2_C*T*_*x*–400_ is slower in the experiment designed to recover and characterize the activated catalyst. This is explained by a less efficient gas–solid contacting when using an undiluted (and therefore low volume) Mo_2_C*T*_*x*–400_ bed and a larger diameter of the reactor used (see experimental section for details).

Mo 3d XPS analysis of Mo_2_C*T*_*x*–400–TOS2h_ and Mo_2_C*T*_*x*–500–TOS2h_ shows little changes when compared to fresh Mo_2_C*T*_*x*–400_ and Mo_2_C*T*_*x*–500_ (Figure 2f, Figure S4, and Table S7). Interestingly, Mo_2_C*T*_*x*–500_ remains fully defunctionalized despite the presence of oxygenates (CO_2_ and water) under FT conditions. For Mo_2_C*T*_*x*–400–TOS2h_, there is a minor increase in its carbidic Mo component, from 38 to 41% relative to Mo_2_C*T*_*x*–400_, which is paralleled by a rise of 4% in its CO conversion during 2 h TOS. Turning to the C 1s XPS region, Mo_2_C*T*_*x*–400–TOS2h_ shows an additional broad peak at a binding energy of ca. 288.9 eV, assigned to molecularly adsorbed CO*. A similar peak was observed by us previously on the active state of a 2D-Mo_2_C_1–__*x*_O*_y_* reverse water–gas shift catalyst.^50^ In contrast, the peaks due to adsorbed CO*, C–O, and C=O are absent in Mo_2_C*T*_*x*–500–TOS2h_, which only shows a MXene peak due to carbidic carbon (Figure 2f).^50^ This observation correlates with a notably higher CO conversion in Mo_2_C*T*_*x*–500_ relative to Mo_2_C*T*_*x*–400_. The atomic ratio of Mo to C^carb^ in Mo_2_C*T*_*x*–400–TOS2h_ is found to be 1.9:1 (Figure S12), which is the same as in Mo_2_C*T*_*x*–400_, as discussed above. In contrast, fitting of Mo_2_C*T*_*x*–500–TOS2h_ reveals a ratio of 2.6:1, which is slightly lower than 2.8:1 in Mo_2_C*T*_*x*–500_. The result indicates that the content of carbidic carbon in Mo_2_C*T*_*x*–500_ may increase slightly under FT conditions within 2 h of TOS. Overall, the substoichiometric ratio between Mo and carbidic carbon in Mo_2_C*T*_*x*–500–TOS2h_ parallels the high and stable methanation selectivity (and low FT selectivity) displayed by Mo_2_C*T*_*x*–500_. Although elucidating the origin of the high methanation selectivity of Mo_2_C*T*_*x*–500_ is beyond the scope of this work, the methanation mechanism may involve carbon vacancy sites of Mo_2_C*T*_*x*–500_.

SEM images of the as-prepared and activated catalysts show that the layered nanoplatelet morphology is preserved during the FT reaction (Figure S13). XRD analysis of Mo_2_C*T*_*x*–500–TOS2h_ and Mo_2_C*T*_*x*–400–TOS2h_, both opened to air, confirms the maintenance of a 2D morphology (cell parameter *c* of 15.50 and 15.45 Å, respectively) and the absence of any new crystalline phases (Figure S14).

A note concerning the determination of the amount of Mo surface sites in our 2D catalysts is that determining the quantity of Mo surface sites by CO chemisorption is challenging because of the low temperature of CO desorption from Mo carbides; that is, 2D-Mo_2_C features a broad CO desorption peak centered at ca. 24 °C,^50^ necessitating the use of low-temperature CO chemisorption experiments to ensure that a full CO coverage is being measured (details about the CO chemisorption experiments are provided in the Supporting Information). However, performing CO chemisorption at −30 °C likely results in gas diffusion limitations into the interlayer space of MXenes, as can be seen from a (unexpected) higher CO chemisorption capacity of Mo_2_C*T*_*x*–400_ relative to Mo_2_C*T*_*x*–500_ (Table S3). Low-temperature gas diffusion limitation is a known issue for the determination of specific surface area of MXenes using N_2_ physisorption (i.e., reported surface area values are notably lower than theoretically predicted values; see further details in the SI).

**Figure 3:**
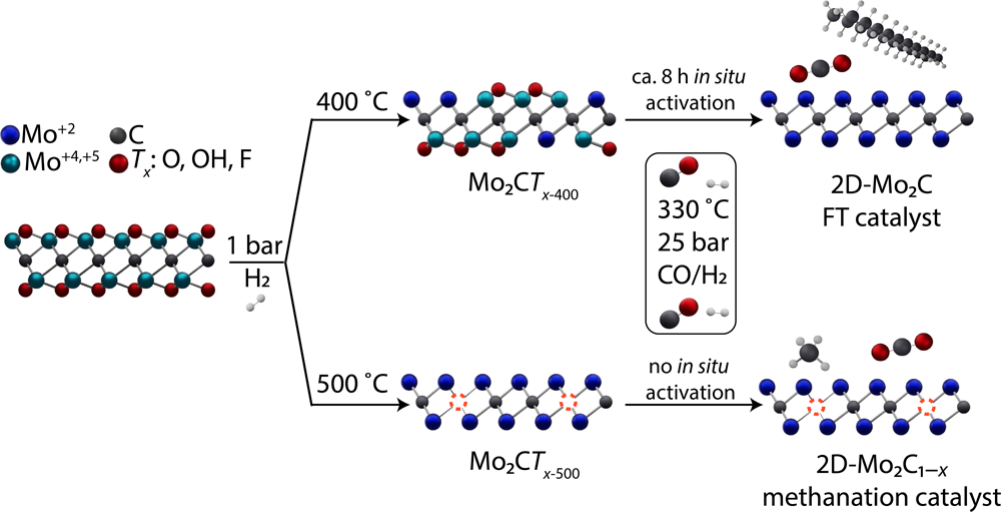
Schematic representation of the likely routes of reductive defunctionalization of Mo_2_C*T*_*x*_ in H_2_ and under FT conditions and implications for FT selectivity.

To conclude, the higher CO conversion displayed by Mo_2_C*T*_*x*–500_ relative to Mo_2_C*T*_*x*–400_ (prior to *in situ* activation) correlates with the absence of *T*_*x*_ passivating species in Mo_2_C*T*_*x*–500–TOS2h_ and the presence of *T*_*x*_ species in Mo_2_C*T*_*x*–400–TOS2h_, as shown by the detection of Mo^4+^ and Mo^5+^ electronic states, in addition to the carbidic Mo^2+^ state, in Mo_2_C*T*_*x*–400–TOS2h_. The selectivity of Mo_2_C*T*_*x*–500_ to C_5+_ (i.e., FT products) is low, while its selectivity to methane is high, and this correlates with the substoichiometric ratio of Mo to carbidic carbon in both Mo_2_C*T*_*x*–500_ and Mo_2_C*T*_*x*–500–TOS2h_, owing to a loss of carbidic carbon during the H_2_ pretreatment at 500 °C. Carbon vacancies in Mo_2_C*T*_*x*–500_ are not readily replenished under the FT testing conditions used in this work (i.e., there is only a small increase of carbidic carbon in Mo_2_C*T*_*x*–500–TOS2h_ relative to Mo_2_C*T*_*x*–500_ that is within the experimental uncertainty). In contrast, H_2_ pretreatment of Mo_2_C*T*_*x*_ at 400 °C does not form significant amounts of carbon vacancies while exposure of Mo_2_C*T*_*x*–400_ to the FT conditions leads to the *in situ* removal of the remaining *T*_*x*_ passivating species (without the concomitant formation of carbon vacancies). The active state of Mo_2_C*T*_*x*–400_ after *in situ* activation can therefore be described as 2D-Mo_2_C, while the active state of Mo_2_C*T*_*x*–500_ is more correctly described as 2D-Mo_2_C_1–*x*_ (Figure 3). These results underline the importance of optimized reductive surface defunctionalization protocols to achieve the full potential of Mo_2_C*T*_*x*_-derived catalysts in FT. In the following, we rationalize the selectivities displayed by 2D-Mo_2_C using DFT calculations and map out the most likely FT reaction pathway.

## DFT Study

### Model Surface

**Figure 4:**
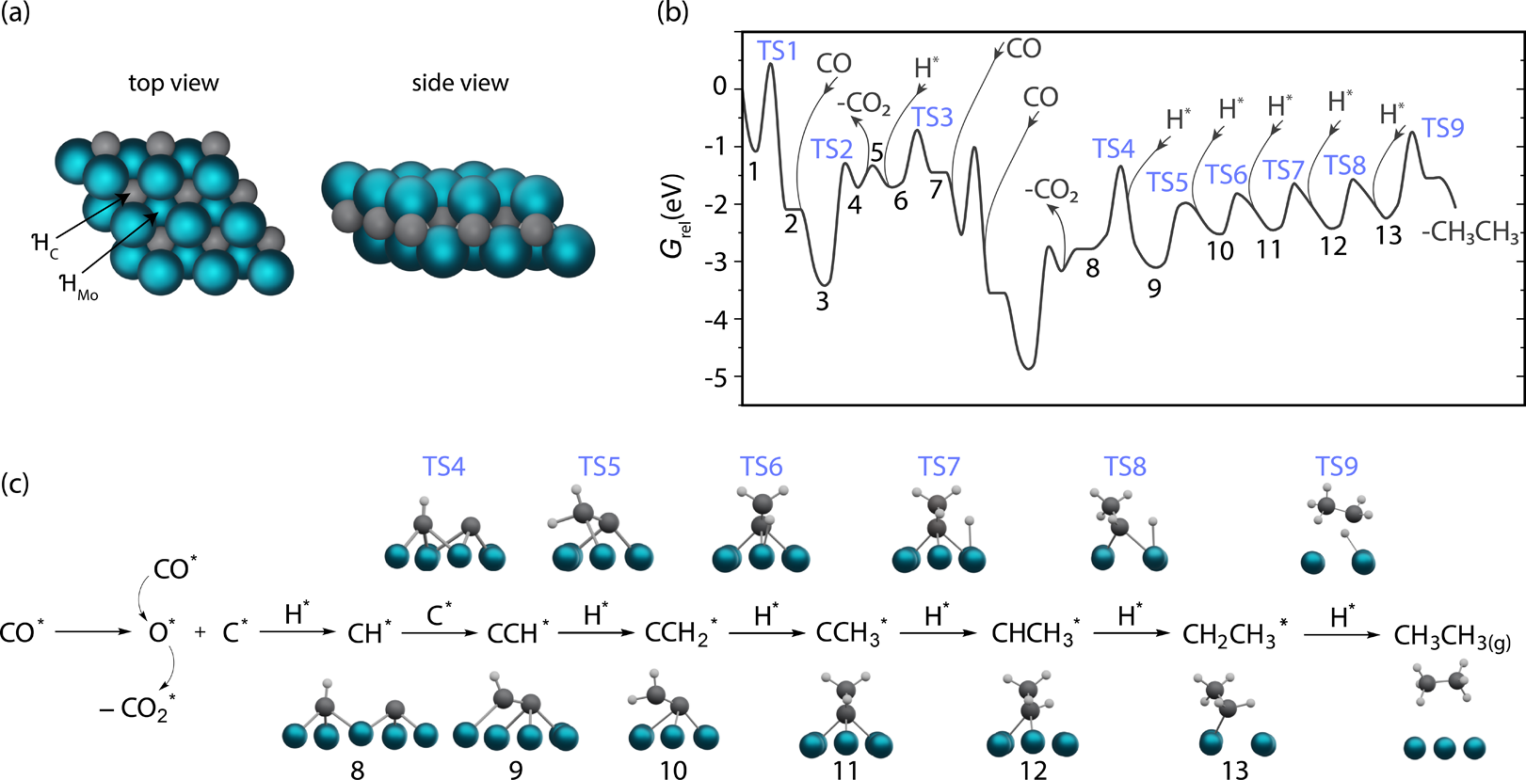
(a) Top and side views of the 2D-Mo_2_C DFT model showing 3-fold hollow sites over a Mo atom () and over a carbon atom (). (b) Energy profile for ethane formation including the C*–CH* coupling steps. (c) Key steps of the calculated reaction mechanism on 2D-Mo_2_C with selected transition states and intermediates. Energies are referenced against the sum of the reactants’ energy (4 CO and 3 H_2_) and the catalytic surface in eV (*G*_rel_). The pathway connecting intermediates **1** and **4** is repeated also between intermediates **7** and **8**.

For our DFT model, we used a fully defunctionalized surface of 2D-Mo_2_C (i.e., absence of any O*) species as a representative model of the Mo_2_C*T*_*x*-400_ catalyst in the steady state, to calculate the Gibbs energy profile and obtain mechanistic insights. As discussed above, the choice of this model is consistent with the presence of only carbidic Mo in the Mo 3d XPS spectrum of active Mo_2_C*T*_*x*–500–TOS2h_, as well as the lack of peaks due to CO* species in the C 1s XPS region, which excludes the presence of a CO* adlayer. The model corresponds to the Mo_2_C (0001) surface and consists of two exterior Mo layers and a central carbon layer sandwiched by two Mo layers.^56,60^ First, we examined the adsorption of carbon, hydrogen, and oxygen on the 3-fold hollow, bridge, and on-top sites (Figure 4a and Figure S17). The adsorption energies and reference energies are given in Table S6. The DFT results show that H* and C* adsorb preferentially on 3-fold hollow sites above a Mo atom (denoted , Figure 4a), whereas O* adsorbs preferentially on 3-fold hollow sites above a C atom (denoted , Figure 4a). The calculated Gibbs energy profiles and snapshots of selected transition states for ethane formation are shown in Figure 4b and c, respectively.

### Dissociation of CO and H_2_ and O* Removal

Adsorption of carbon monoxide on a  site is exergonic by 1.07 eV. CO binds via carbon in a μ_3_-η^1^ fashion orthogonal to the surface. This interaction weakens the C–O bond that elongates by 0.05 Å, consistent with a decrease in the calculated stretching frequency from 2129 to 1670 cm^–1^ owing to π-back-donation and rehybridization of CO.^61^ In the unassisted dissociation pathway, CO* species **1** tilts toward the surface and forms C* and O* species in vicinal 3-fold hollow sites ( and , respectively; Figure S18). This state is denoted **2** (Figure S19). The associated transition state TS1 has a Gibbs energy barrier of 1.51 eV. The direct CO dissociation step is exergonic by 1.02 eV, which is similar to the values reported for the (100) surface of β-Mo_2_C.^35^ The dissociative chemisorption of H_2_ on the 2D-Mo_2_C surface starts from a physisorbed state with a shallow minimum of −20 meV and proceeds to dissociated H_2_ via a small energy barrier (0.15 eV).^62,63^ In this process, the distance from the surface to the center-of-mass of H_2_ decreases from 3.1 to1.4 Å while *d*_H–H_ increases from 0.74 to 3.03 Å (two H*), corresponding to the distance between two adjacent  sites. The Gibbs energy barrier for the recombination of C* and O* species on 2D-Mo_2_C is 2.53 eV, which is considerably higher than the Gibbs energy barrier for the hydrogenation of C* to CH* (0.90 eV), indicating that the direct dissociation of CO* is essentially irreversible in our reaction conditions (330 °C and 25 bar).

Next, we evaluated the energetics of the H_2_-assisted pathways for CO activation and they were found to be less favorable than TS1, owing to the higher energy barriers of 1.77 and 2.18 eV for the formyl and hydroxycarbonyl routes, respectively (Figure S19). Further details on the H_2_-assisted pathways are discussed in the Supporting Information. The O* species formed via the direct dissociation of CO* can be removed as CO_2_ through its reaction with CO* or as H_2_O via the reaction with 2H*. The formation of CO_2_ requires CO* to adopt a distorted μ^3^-η^2^ coordination in the intermediate **3**, located at −3.35 eV with respect to initial reactants. In the transition state TS2, O* migrates from a vicinal  site atop the intersecting Mo atom (that is, the Mo atom that separates the  and  sites, Figure S18) with a barrier of 2.05 eV. In the product, the C atom of CO_2_* is on a bridge position and the O atoms are located on top positions (atop) of adjacent Mo atoms (**4**). The Gibbs desorption energy of a CO_2_ molecule under reaction conditions is 0.44 eV. Therefore, the Gibbs energy required to remove O* through a reaction with CO* (intermediate **3**) and regenerate the active site is 2.02 eV. The removal of O* species via the hydrogenation of a hydroxyl yielding water has a barrier of 2.56 eV and is, therefore, less favorable. O* removal via proton transfer between neighboring hydroxyls has a an even higher energy barrier of 2.66 eV. These kinetically less favorable routes are endergonic by 2.15 eV and are discussed in detail in the SI (Figure S20).

### C–C Coupling and Hydrogenation

Subsequently, we calculated the energetics of the potential C–C coupling and hydrogenation steps for ethane formation to investigate the chain growth mechanism. The calculated Gibbs energies are given in Table S4, and the optimized geometries of the initial, transition, and final states are presented in Figures S21–S24. Figure S25 shows the Gibbs energy of the respective energy barriers plotted against the Gibbs reaction energy for the elementary steps. Generally, hydrogenation steps have lower activation barriers to form C_*x*_H_*y*_ species with larger *y*. In contrast, the C–C coupling steps have lower activation barriers for lower *y* with one exception that is the coupling between two C* species, which has an energy barrier of 1.43 eV, higher than the coupling barrier between C* and CH* (1.20 eV). The average barrier for the hydrogenation of various C_*x*_H_*y*_ species via reactions presented in Table S4 (reactions 19–34) is ca. 1.00 (±0.29) eV. Therefore, since the hydrogenation of C_*x*_H_y_* involves lower barriers than the one calculated for the H*-assisted activation of CO* (1.77 and 2.18 eV for the formyl and hydroxycarbonyl routes, respectively), H* will be consumed preferentially through the hydrogenation of C_*x*_H_y_* rather than through the H-assisted activation of CO*. The average barrier for C–C coupling, presented in Table S4 (reactions 10–18), is ca. 1.35 (±0.22) eV, i.e., higher than the average barrier for the hydrogenation of C_*x*_H_y_* species. However, although the coupling of two CH* species (**14** in Figure S26) is energetically favored, with the lowest Gibbs energy barrier of 0.85 eV among the possible coupling steps, the high bonding energy of CHCH* species to the surface (−2.75 eV) leads to a high Gibbs energy barrier of 1.41 eV (TS11) for transferring H* to convert CHCH* into CHCH_2_* (**16** in Figure S26). The high energy barrier to form CHCH_2_* and consequently also CH_2_CH_2_* species is consistent with the absence of ethene in the experimental product distribution.

An alternative FT pathway involves the coupling between two CH* species and the H*-assisted transformation of acetylene to ethylidyne (i.e., ≡CCH_3_*).^64^ In this alkylidyne mechanism, H* adsorbed on an  site in close proximity to the bound acetylene (**15** in Figure S27), induces a hydrogen transfer from one CH group of acetylene to another. The barrier for this process is 2.26 eV (TS24 in Figure S27). The formed vinylidene (i.e., =C=CH_2_*) species features the sp carbon residing over an  site and the methylidene fragment over a Mo atom. At this point, the transfer of H* from the  site to the Mo atom interacting with the methylidene fragment converts the =C=CH_2_* species to ≡CCH_3_* (**11** in Figure S27). Overall, this alternative route is not only associated with a high barrier (i.e., it is kinetically unfavorable) but also endergonic by 1.24 eV and is therefore an unlikely FT pathway on 2D-Mo_2_C.

The C–C coupling route with the second lowest barrier occurs between C* and CH* species (**8**, Figure 4). Between states **7** (that corresponds to methylidyne, CH*) and **8** in Figure 4, states **1** to **4** are repeated to account for the deposition of an additional C* on the surface. This step involves the migration of C* and CH* species from vicinal  sites to a bridge position, via a TS4 with an energy barrier of 1.20 eV, forming CCH* (**9**). The CCH* species adsorbs parallel to the surface, and the H atom of CCH* does not interact with the surface. The barrier for adding adsorbed H* species to CCH* yielding CCH_2_* is 0.98 eV (TS5), i.e., 0.43 eV, lower than for the hydrogenation of CCH* to give CHCH* species. This low energy barrier suggests that ethane formation occurs on 2D-Mo_2_C via the CCH* and CCH_2_* intermediates. The hydrogenation of CCH* species proceeds in the bridge position, such that the C–C axis of the resulting CCH_2_* is nearly parallel to the surface (**10**). Adding a third H* to CCH_2_* to form CCH_3_* requires a reorientation of the molecular axis of CCH_2_* toward a configuration orthogonal to the surface with the hydrogenated C on top of a Mo atom and the bare C still over an  site. In the transition state (TS6), the H–C–H angle (as seen from above, Figure S23) decreases from ca. 180° to ca. 120°, with an energy cost of 0.70 eV. After H* addition, only one of the H atoms of the formed ethylidyne interacts with the Mo atom in intermediate **11**, as seen from the significant elongation of the C–H bond of the interacting atom (1.18 Å) compared to the other two C–H bonds (1.09 Å).

No notable geometrical change occurs, while a further hydrogen migrates from an  site over a Mo atom to yield the CHCH_3_* species, associated with an energy barrier of 0.75 eV (TS7). In the CHCH_3_* species, the methine hydrogen interacts with one of the two remaining vicinal Mo atoms. Adding a further H* to CHCH_3_* to form CH_2_CH_3_* via TS8 has a Gibbs energy barrier of 0.81 eV and preserves the geometry of the CHCH_3_* species. Both H atoms of the CH_2_ group interact with Mo atoms (intermediate **12**). For the final hydrogenation step, this interaction breaks such that only one H of CH_2_ interacts with the surface (intermediate **13**). In the transition state TS9 (1.28 eV), the methylene carbon decoordinates from the 3-fold hollow site and moves atop the neighboring Mo atom while the H–C–H angle decreases from 180 to 120° (Figure S23). The hydrogenation of the CH_2_CH_3_* species to ethane has a Gibbs energy barrier equal to 1.28 eV (TS9), which is lower than the barrier to form CH_4_ from CH_3_* species (1.57 eV, Figure S28). Note that the energetic cost to form CH_4_ from CH_3_* also exceeds the average barrier for the C–C coupling steps discussed above. Therefore, our DFT results suggest that chain propagation is favored over methanation, in agreement with the experimental observations.

Lastly, to provide a rationale for the lower CO conversion rate of Mo_2_C*T*_*x*–400–TOS1h_ relative to Mo_2_C*T*_*x*–400–TOS8h_ (i.e., prior to and after *in situ* activation), we considered a 2D-Mo_2_C-0.67 O ML model and calculated also for this model the Gibbs energy barriers and Gibbs reaction energies of the key elementary steps identified for the 2D-Mo_2_C model (*vide supra*). Results show that the lower activity of the surface with 0.67 O* ML compared to the pristine surface can be attributed to the significantly higher barrier for the dissociation of CO on 2D-Mo_2_C-0.67 O ML relative to 2D-Mo_2_C. Further details and results for the 2D-Mo_2_C-0.67 O ML model are provided in the SI (Table S5 and Figure S29).

## Discussion

Since the first catalytic applications of Mo_2_C, its reactivity was generally compared to that of Ru.^65^ More recently, it has been reported that the adsorption energies of C-containing intermediates on the Mo-terminated (100) surface of 3D-Mo_2_C are indeed similar to that of Ru (in particular the (211) surface).^35^ However, the adsorption energies of O-containing intermediates on the β-Mo_2_C (100) surface are significantly higher (i.e., more negative) due to the oxophilicity of Mo.^35^ Interestingly, DFT studies of Ru surfaces have shown that in the absence of a dense CO* adlayer, CO* undergoes a direct dissociation to C* and O* species on stepped sites.^66−68^ DFT results presented here also suggest the direct dissociation of CO on the 2D-Mo_2_C surface in the absence of a CO* adlayer, showing further similarities with Ru surfaces.

Our DFT calculations identify notable differences between the reaction barriers and the stability of intermediates on 2D-Mo_2_C and those reported for 3D-Mo_2_C. For instance, on 3D-Mo_2_C, it has been suggested that the H-assisted CO dissociation pathway prevails. In this route, HCO* is formed first, which subsequently dissociates into CH* and O*. The formation of the HCO* intermediate was associated with a low barrier for different facets of 3D-Mo_2_C (energies ranging from 0.12 to 0.36 eV; note that these reported energies are total energy or electronic energy with a zero-point-energy correction),^69,70^ compared to 1.04 eV for the 2D-Mo_2_C (0001) surface. Furthermore, the formation of HCO* is strongly endergonic for 2D-Mo_2_C (1.04 eV) and can vary from exergonic (−0.32 eV) to mildly endergonic (0.13 eV) on 3D-Mo_2_C.^69,70^ Therefore, the hydrogenation of CO* to HCO* is both kinetically and thermodynamically less favorable on 2D-Mo_2_C than on surfaces of 3D-Mo_2_C. The high endothermicity of steps associated with the formation of HCO* and COH* intermediates (1.04 and 1.17 eV, respectively) on the 2D-Mo_2_C (0001) surface is generally consistent with the absence of oxygenates in the reaction products, suggesting the prevalence of the carbidic chain growth mechanism.

The O* removal from the 2D-Mo_2_C (0001) surface is endergonic, with barriers as high as ca. 2 and 2.7 eV for CO_2_ and H_2_O, respectively, owing to the high Mo–O bond strength.^71−74^ In contrast to the desorption of H_2_O, steps associated with the dissociation of hydrogenated oxygen-containing species (OH* and H_2_O*) have lower energy barriers, that is, reactions OH* → O* + H* and H_2_O → OH* + H* proceed via transition states that are only 0.98 and 0.77 eV high, respectively. This indicates that the dissociation of hydrogenated oxygen-containing species occurs faster than the removal of water (assuming similar pre-exponential factors). Remembering that H* has lower barriers for its reaction with C-containing species than with O* or OH* species (Figure S20), one can conclude that CO_2_ is the preferred oxygenate product, which agrees well with the high experimental CO_2_ selectivity. A high rate of CO_2_ formation on 2D-Mo_2_C indicates a high activity for the water gas shift reaction. Interestingly, WGS occurs in FT conditions already at 330 °C, a significantly lower temperature than previously observed for Mo_2_C*T*_*x*_ (ca. 450–500 °C).^49^ This is explained by the fully functionalized surface of Mo_2_C*T*_*x*_ in WGS conditions and a (fully) defunctionalized surface under FT conditions.^32^

In conclusion, a MXene-derived 2D-Mo_2_C-based catalyst, prepared via *in situ* activation under FT conditions, enables the hydrogenation of CO to higher alkanes with a chain growth probability coefficient α of 0.87. The value of α is ca. two times higher than reported previously for other molybdenum carbides. The CO conversion rate of MXene-based catalysts depends strongly on the extent of defunctionalization of the surface passivating groups (*T*_*x*_) such that fully defunctionalized 2D-Mo_2_C and 2D-Mo_2_C_1–*x*_ catalysts show notably higher gravimetric CO conversion rates relative to only a partially defunctionalized catalyst (i.e., initial Mo_2_C*T*_*x*–400_). However, the gravimetric CO consumption rates of 2D catalysts are significantly higher, for both fully and partially defunctionalized catalysts, relative to a reference 3D β-Mo_2_C_(400)_, underlining a yet unharnessed potential of 2D materials such as MXenes in heterogeneous catalysis. In the FT synthesis conditions used here, the partially defunctionalized catalyst Mo_2_C*T*_*x*–400_ undergoes a strong *in situ* activation explained by the reductive defunctionalization of the *T*_*x*_ groups in Mo_2_C*T*_*x*–400_ to form a 2D-Mo_2_C state. Progressive defunctionalization of Mo_2_C*T*_*x*–400_ leads also to an increase in the WGS activity (evidenced by a higher CO_2_ selectivity). The concomitant increase in CO conversion leads to an overall higher hydrocarbon productivity, in particular for C_5+_ products. H_2_ pretreatment at 500 °C does not only fully defunctionalize the passivating *T*_*x*_ groups in Mo_2_C*T*_*x*_ but also partly removes carbidic carbon of Mo_2_C*T*_*x*_, yielding a 2D-Mo_2_C_1–*x*_ catalyst active in CO methanation. In contrast, a 2D-Mo_2_C catalyst prepared via the *in situ* activation of Mo_2_C*T*_*x*–400_ does not feature a depleted content of carbidic carbon and is selective in FT. DFT calculations identified feasible energy profiles for the chain growth mechanism on a 2D-Mo_2_C (0001) surface under reaction conditions and in the absence of a CO adlayer. In particular, according to DFT results, CO directly dissociates into C* and O*, consistent with the absence of oxygenate products (beyond CO_2_). The high barrier for the hydrogenation of CH_3_* species to methane relative to the lower chain growth barrier explains the formation of higher alkanes. Oxygen removal is the rate-limiting step, owing to the high oxophilicity of the carbidic surface, with CO_2_ being the major reaction product (WGS reaction).

## Supporting Information Available

Experimental procedures, characterization, computational details, additional computational results, XPS and Raman spectra, TPD and CO chemisorption results, XRD diffractograms, and catalytic results (PDF)

## References

[ref1] Dry M. E. The Fischer–Tropsch Process: 1950–2000. Catal. Today 2002, 71, 227–241. 10.1016/S0920-5861(01)00453-9.

[ref2] Rommens K. T.; Saeys M. Molecular Views on Fischer–Tropsch Synthesis. Chem. Rev. 2023, 123, 5798–5858. 10.1021/acs.chemrev.2c00508. 36897768

[ref3] Fratalocchi L.; Visconti C. G.; Groppi G.; Lietti L.; Tronconi E. Intensifying Heat Transfer in Fischer–Tropsch Tubular Reactors through the Adoption of Conductive Packed Foams. Chem. Eng. J. 2018, 349, 829–837. 10.1016/j.cej.2018.05.108.

[ref4] Filot I. A. W.; Broos R. J. P.; van Rijn J. P. M.; van Heugten G. J. H. A.; van Santen R. A.; Hensen E. J. M. First-Principles-Based Microkinetics Simulations of Synthesis Gas Conversion on a Stepped Rhodium Surface. ACS Catal. 2015, 5, 5453–5467. 10.1021/acscatal.5b01391.

[ref5] Rofer-DePoorter C. K. A Comprehensive Mechanism for the Fischer–Tropsch Synthesis. Chem. Rev. 1981, 81, 447–474. 10.1021/cr00045a002.

[ref6] Bezemer G. L.; Bitter J. H.; Kuipers H. P. C. E.; Oosterbeek H.; Holewijn J. E.; Xu X.; Kapteijn F.; van Dillen A. J.; de Jong K. P. Cobalt Particle Size Effects in the Fischer–Tropsch Reaction Studied with Carbon Nanofiber Supported Catalysts. J. Am. Chem. Soc. 2006, 128, 3956–3964. 10.1021/ja058282w. 16551103

[ref7] Filot I. A. W.; van Santen R. A.; Hensen E. J. M. The Optimally Performing Fischer–Tropsch Catalyst. Angew. Chem., Int. Ed. 2014, 53, 12746–12750. 10.1002/anie.201406521. 25168456

[ref8] Khodakov A. Y.; Chu W.; Fongarland P. Advances in the Development of Novel Cobalt Fischer–Tropsch Catalysts for Synthesis of Long-Chain Hydrocarbons and Clean Fuels. Chem. Rev. 2007, 107, 1692–1744. 10.1021/cr050972v. 17488058

[ref9] Ordomsky V. V.; Luo Y.; Gu B.; Carvalho A.; Chernavskii P. A.; Cheng K.; Khodakov A. Y. Soldering of Iron Catalysts for Direct Synthesis of Light Olefins from syngas under Mild Reaction Conditions. ACS Catal. 2017, 7, 6445–6452. 10.1021/acscatal.7b01307.

[ref10] Subramanian V.; Cheng K.; Lancelot C.; Heyte S.; Paul S.; Moldovan S.; Ersen O.; Marinova M.; Ordomsky V. V.; Khodakov A. Y. Nanoreactors: an Efficient Tool to Control the Chain-Length Distribution in Fischer–Tropsch Synthesis. ACS Catal. 2016, 6, 1785–1792. 10.1021/acscatal.5b01596.

[ref11] Chen Y.; Batalha N.; Marinova M.; Impéror-Clerc M.; Ma C.; Ersen O.; Baaziz W.; Stewart J. A.; Curulla-Ferré D.; Khodakov A. Y.; et al. Ruthenium Silica Nanoreactors with Varied Metal–Wall Distance for Efficient Control of Hydrocarbon Distribution in Fischer–Tropsch Synthesis. J. Catal. 2018, 365, 429–439. 10.1016/j.jcat.2018.06.023.

[ref12] Zaman S.; Smith K. J. A Review of Molybdenum Catalysts for Synthesis Gas Conversion to Alcohols: Catalysts, Mechanisms and Kinetics. Catal. Rev. - Sci. Eng. 2012, 54, 41–132. 10.1080/01614940.2012.627224.

[ref13] Patterson P. M.; Das T. K.; Davis B. H. Carbon Monoxide Hydrogenation over Molybdenum and Tungsten Carbides. Appl. Catal. A: Gen. 2003, 251, 449–455. 10.1016/S0926-860X(03)00371-5.

[ref14] Schaidle J. A.; Thompson L. T. Fischer–Tropsch Synthesis over Early Transition Metal Carbides and Nitrides: CO Activation and Chain Growth. J. Catal. 2015, 329, 325–334. 10.1016/j.jcat.2015.05.020.

[ref15] Kim H.-G.; Lee K. H.; Lee J. S. Carbon Monoxide Hydrogenation over Molybdenum Carbide Catalysts. Res. Chem. Intermed. 2000, 26, 427–443. 10.1163/156856700X00435.

[ref16] Griboval-Constant A.; Giraudon J. M.; Leclercq G.; Leclercq L. Catalytic Behaviour of Cobalt or Ruthenium Supported Molybdenum Carbide Catalysts for FT Reaction. Appl. Catal. A: Gen. 2004, 260, 35–45. 10.1016/j.apcata.2003.10.031.

[ref17] Woo H. C.; Park K. Y.; Kim Y. G.; Nam I. S.; Chung J. S.; Lee J. S. Mixed Alcohol Synthesis from Carbon Monoxide and Dihydrogen over Potassium-Promoted Molybdenum Carbide Catalysts. Appl. Catal. 1991, 75, 267–280. 10.1016/S0166-9834(00)83136-X.

[ref18] Wu Q.; Christensen J. M.; Chiarello G. L.; Duchstein L. D. L.; Wagner J. B.; Temel B.; Grunwaldt J.-D.; Jensen A. D. Supported Molybdenum Carbide for Higher Alcohol Synthesis from syngas. Catal. Today 2013, 215, 162–168. 10.1016/j.cattod.2013.03.002.

[ref19] Li T.; Virginie M.; Khodakov A. Y. Effect of Potassium Promotion on the Structure and Performance of Alumina Supported Carburized Molybdenum Catalysts for Fischer–Tropsch Synthesis. Appl. Catal. A: Gen. 2017, 542, 154–162. 10.1016/j.apcata.2017.05.018.

[ref20] Vo D.-V. N.; Nguyen T.-H.; Kennedy E. M.; Dlugogorski B. Z.; Adesina A. A. Fischer–Tropsch Synthesis: Effect of Promoter Type on Alumina-Supported Mo Carbide Catalysts. Catal. Today 2011, 175, 450–459. 10.1016/j.cattod.2011.04.045.

[ref21] Fischer F.; Tropsch H. Über die Direkte Synthese von Erdöl-Kohlenwasserstoffen bei Gewöhnlichem Druck. (Zweite Mitteilung.). Ber. Dtsch. Chem. Ges. 1926, 59, 832–836. 10.1002/cber.19260590443.

[ref22] Biloen P.; Sachtler W. M. H. Mechanism of Hydrocarbon Synthesis over Fischer–Tropsch Catalysts. In Adv. Catal., Eley D. D., Pines H., Weisz P. B., Eds.; Vol. 30; Academic Press, 1981; pp 165–216.

[ref23] Craxford S. R.; Rideal E. K. 338. The Mechanism of the Synthesis of Hydrocarbons from Water Gas. J. Chem. Soc. 1939, 1604–1614. 10.1039/jr9390001604.

[ref24] Brady R. C. III; Pettit R. Reactions of Diazomethane on Transition-Metal Surfaces and Their Relationship to the Mechanism of the Fischer–Tropsch Reaction. J. Am. Chem. Soc. 1980, 102, 6181–6182. 10.1021/ja00539a053.

[ref25] Joyner R. W. Mechanism of Hydrocarbon Synthesis from Carbon Monoxide and Hydrogen. J. Catal. 1977, 50, 176–180. 10.1016/0021-9517(77)90020-3.

[ref26] Maitlis P. M.; Zanotti V. Organometallic Models for Metal Surface Reactions: Chain Growth Involving Electrophilic Methylidynes in the Fischer–Tropsch Reaction. Catal. Lett. 2008, 122, 80–83. 10.1007/s10562-007-9359-3.

[ref27] Ciobîcă I. M.; Kramer G. J.; Ge Q.; Neurock M.; van Santen R. A. Mechanisms for Chain Growth in Fischer–Tropsch Synthesis over Ru(0001). J. Catal. 2002, 212, 136–144. 10.1006/jcat.2002.3742.

[ref28] Weststrate C. J.; Sharma D.; Garcia Rodriguez D.; Gleeson M. A.; Fredriksson H. O. A.; Niemantsverdriet J. W. Mechanistic Insight into Carbon-Carbon Bond Formation on Cobalt under Simulated Fischer–Tropsch Synthesis Conditions. Nat. Commun. 2020, 11, 750 10.1038/s41467-020-14613-5. 32029729 PMC7005166

[ref29] Liu Z.-P.; Hu P. A New Insight into Fischer–Tropsch Synthesis. J. Am. Chem. Soc. 2002, 124, 11568–11569. 10.1021/ja012759w. 12296701

[ref30] Pichler H.; Schulz H. Neuere Erkenntnisse auf dem Gebiet der Synthese von Kohlenwasserstoffen aus CO und H_2_. Chem. Ing. Technol. 1970, 42, 1162–1174. 10.1002/cite.330421808.

[ref31] Storch H. H.; Golumbic N.; Anderson R. B. The Fischer–Tropsch and Related Syntheses: Including a Summary of Theoretical and Applied Contact Catalysis; Wiley, 1951; p 592.

[ref32] Ranhotra G. S.; Bell A. T.; Reimer J. A. Catalysis over Molybdenum Carbides and Nitrides: II. Studies of CO hydrogenation and C_2_H_6_ hydrogenolysis. J. Catal. 1987, 108, 40–49. 10.1016/0021-9517(87)90153-9.

[ref33] Posada-Perez S.; Vines F.; Ramirez P. J.; Vidal A. B.; Rodriguez J. A.; Illas F. The Bending Machine: CO_2_ activation and hydrogenation on δ-MoC(001) and β-Mo_2_C(001) Surfaces. Phys. Chem. Chem. Phys. 2014, 16, 14912–14921. 10.1039/C4CP01943A. 24931917

[ref34] Kojima I.; Miyazaki E. Catalysis by Transition Metal Carbides: V. Kinetic Measurements of Hydrogenation of CO over TaC, TiC, and Mo_2_C catalysts. J. Catal. 1984, 89, 168–171. 10.1016/0021-9517(84)90292-6.

[ref35] Medford A. J.; Vojvodic A.; Studt F.; Abild-Pedersen F.; Nørskov J. K. Elementary Steps of syngas Reactions on Mo_2_C(001): Adsorption Thermochemistry and Bond Dissociation. J. Catal. 2012, 290, 108–117. 10.1016/j.jcat.2012.03.007.

[ref36] Kurlov A.; Huang X.; Deeva E. B.; Abdala P. M.; Fedorov A.; Müller C. R. Molybdenum Carbide and Oxycarbide from Carbon-Supported MoO_3_ Nanosheets: Phase Evolution and DRM Catalytic Activity Assessed by TEM and *In Situ* XANES/XRD Methods. Nanoscale 2020, 12, 13086–13094. 10.1039/D0NR02908D. 32542244

[ref37] Schaidle J. A.; Blackburn J.; Farberow C. A.; Nash C.; Steirer K. X.; Clark J.; Robichaud D. J.; Ruddy D. A. Experimental and Computational Investigation of Acetic Acid Deoxygenation over Oxophilic Molybdenum Carbide: Surface Chemistry and Active Site Identity. ACS Catal. 2016, 6, 1181–1197. 10.1021/acscatal.5b01930.

[ref38] Sullivan M. M.; Chen C.-J.; Bhan A. Catalytic Deoxygenation on Transition Metal Carbide Catalysts. Catal. Sci.Technol. 2016, 6, 602–616. 10.1039/C5CY01665G.

[ref39] Sullivan M. M.; Bhan A. Effects of Oxygen Coverage on Rates and Selectivity of Propane-CO_2_ Reactions on Molybdenum Carbide. J. Catal. 2018, 357, 195–205. 10.1016/j.jcat.2017.11.004.

[ref40] Mo T.; Xu J.; Yang Y.; Li Y. Effect of Carburization Protocols on Molybdenum Carbide Synthesis and Study on its Performance in CO Hydrogenation. Catal. Today 2016, 261, 101–115. 10.1016/j.cattod.2015.07.014.

[ref41] Johnson G. E.; Mitrić R.; Bonačić-Koutecký V.; Castleman A. W. Clusters as Model Systems for Investigating Nanoscale Oxidation Catalysis. Chem. Phys. Lett. 2009, 475, 1–9. 10.1016/j.cplett.2009.04.003.

[ref42] Nørskov J. K.; Bligaard T.; Logadottir A.; Bahn S.; Hansen L. B.; Bollinger M.; Bengaard H.; Hammer B.; Sljivancanin Z.; Mavrikakis M.; Xu Y.; Dahl S.; Jacobsen C. J. H.; et al. Universality in Heterogeneous Catalysis. J. Catal. 2002, 209, 275–278. 10.1006/jcat.2002.3615.

[ref43] Pacchioni G.; Freund H.-J. Controlling the Charge State of Supported Nanoparticles in Catalysis: Lessons from Model Systems. Chem. Soc. Rev. 2018, 47, 8474–8502. 10.1039/C8CS00152A. 29697127

[ref44] Kunkel C.; Viñes F.; Illas F. Surface Activity of Early Transition-Metal Oxycarbides: CO_2_ Adsorption Case Study. J. Phys. Chem. C 2019, 123, 3664–3671. 10.1021/acs.jpcc.8b11942.

[ref45] Naguib M.; Mashtalir O.; Carle J.; Presser V.; Lu J.; Hultman L.; Gogotsi Y.; Barsoum M. W. Two-Dimensional Transition Metal Carbides. ACS Nano 2012, 6, 1322–1331. 10.1021/nn204153h. 22279971

[ref46] Anasori B.; Lukatskaya M. R.; Gogotsi Y. 2D Metal Carbides and Nitrides (MXenes) for Energy Storage. Nat. Rev. Mater. 2017, 2, 16098 10.1038/natrevmats.2016.98.

[ref47] Naguib M.; Mochalin V. N.; Barsoum M. W.; Gogotsi Y. 25th Anniversary Article: MXenes: a New Family of Two-Dimensional Materials. Adv. Mater. 2014, 26, 992–1005. 10.1002/adma.201304138. 24357390

[ref48] Lim K. R. G.; Shekhirev M.; Wyatt B. C.; Anasori B.; Gogotsi Y.; Seh Z. W. Fundamentals of MXene Synthesis. Nat. Synth. 2022, 1, 601–614. 10.1038/s44160-022-00104-6.

[ref49] Deeva E. B.; Kurlov A.; Abdala P. M.; Lebedev D.; Kim S. M.; Gordon C. P.; Tsoukalou A.; Fedorov A.; Müller C. R. *In Situ* XANES/XRD Study of the Structural Stability of Two-Dimensional Molybdenum Carbide Mo_2_C*T*_*x*_: Implications for the Catalytic Activity in the Water–Gas Shift Reaction. Chem. Mater. 2019, 31, 4505–4513. 10.1021/acs.chemmater.9b01105.

[ref50] Zhou H.; Chen Z.; Kountoupi E.; Tsoukalou A.; Abdala P. M.; Florian P.; Fedorov A.; Müller C. R. Two-Dimensional Molybdenum Carbide 2D-Mo_2_C as a Superior Catalyst for CO_2_ Hydrogenation. Nat. Commun. 2021, 12, 5510 10.1038/s41467-021-25784-0. 34535647 PMC8448824

[ref51] Hu C.; Lai C. C.; Tao Q.; Lu J.; Halim J.; Sun L.; Zhang J.; Yang J.; Anasori B.; Wang J.; et al. Mo_2_Ga_2_C: a New Ternary Nanolaminated Carbide. Chem. Commun. 2015, 51, 6560–6563. 10.1039/C5CC00980D. 25768789

[ref52] Meshkian R.; Näslund L.-Å.; Halim J.; Lu J.; Barsoum M. W.; Rosen J. Synthesis of Two-Dimensional Molybdenum Carbide, Mo_2_C, from the Gallium Based Atomic Laminate Mo_2_Ga_2_C. Scripta Mater. 2015, 108, 147–150. 10.1016/j.scriptamat.2015.07.003.

[ref53] Halim J.; Kota S.; Lukatskaya M. R.; Naguib M.; Zhao M.-Q.; Moon E. J.; Pitock J.; Nanda J.; May S. J.; Gogotsi Y.; et al. Synthesis and Characterization of 2D Molybdenum Carbide (MXene). Adv. Funct. Mater. 2016, 26, 3118–3127. 10.1002/adfm.201505328.

[ref54] Kresse G.; Furthmüller J. Efficient Iterative Schemes for Ab Initio Total-Energy Calculations Using a Plane-Wave Basis Set. Phys. Rev. B 1996, 54, 11169–11186. 10.1103/PhysRevB.54.11169. 9984901

[ref55] Kresse G.; Hafner J. Ab Initio Molecular-Dynamics Simulation of the Liquid-Metal–Amorphous-Semiconductor Transition in Germanium. Phys. Rev. B 1994, 49, 14251–14269. 10.1103/PhysRevB.49.14251. 10010505

[ref56] Kurlov A.; Deeva E. B.; Abdala P. M.; Lebedev D.; Tsoukalou A.; Comas-Vives A.; Fedorov A.; Müller C. R. Exploiting Two-Dimensional Morphology of Molybdenum Oxycarbide to Enable Efficient Catalytic Dry Reforming of Methane. Nat. Commun. 2020, 11, 4920 10.1038/s41467-020-18721-0. 33009379 PMC7532431

[ref57] Kamysbayev V.; Filatov A. S.; Hu H.; Rui X.; Lagunas F.; Wang D.; Klie R. F.; Talapin D. V. Covalent Surface Modifications and Superconductivity of Two-Dimensional Metal Carbide MXenes. Science 2020, 369, 979–983. 10.1126/science.aba8311. 32616671

[ref58] Yorulmaz U.; Özden A.; Perkgöz N. K.; Ay F.; Sevik C. Vibrational and Mechanical Properties of Single Layer MXene Structures: a First-Principles Investigation. Nanotechnology 2016, 27, 335702 10.1088/0957-4484/27/33/335702. 27377143

[ref59] Eliason S. A.; Bartholomew C. H. Reaction and Deactivation Kinetics for Fischer–Tropsch Synthesis on Unpromoted and Potassium-Promoted Iron Catalysts. Appl. Catal. A: Gen. 1999, 186, 229–243. 10.1016/S0926-860X(99)00146-5.

[ref60] Zhou H.; Chen Z.; López A. V.; López E. D.; Lam E.; Tsoukalou A.; Willinger E.; Kuznetsov D. A.; Mance D.; Kierzkowska A.; et al. Engineering the Cu/Mo_2_C*T*_*x*_ (MXene) Interface to Drive CO_2_ Hydrogenation to Methanol. Nat. Catal. 2021, 4, 860–871. 10.1038/s41929-021-00684-0.

[ref61] Föhlisch A.; Nyberg M.; Hasselström J.; Karis O.; Pettersson L. G. M.; Nilsson A. How Carbon Monoxide Adsorbs in Different Sites. Phys. Rev. Lett. 2000, 85, 3309–3312. 10.1103/PhysRevLett.85.3309. 11019328

[ref62] Nørskov J. K.; Houmøller A.; Johansson P. K.; Lundqvist B. I. Adsorption and Dissociation of H_2_ on Mg Surfaces. Phys. Rev. Lett. 1981, 46, 257–260. 10.1103/PhysRevLett.46.257.

[ref63] King D. A. Kinetics of Adsorption, Desorption, and Migration at Single-Crystal Metal Surfaces. Crit. Rev. Solid State Mater. Sci. 1978, 7, 167–208. 10.1080/10408437808243438.

[ref64] Weststrate C. J.; van Helden P.; Niemantsverdriet J. W. Reflections on the Fischer–Tropsch Synthesis: Mechanistic Issues from a Surface Science Perspective. Catal. Today 2016, 275, 100–110. 10.1016/j.cattod.2016.04.004.

[ref65] Leclercq L.; Imura K.; Yoshida S.; Barbee T.; Boudart M. Synthesis of New Catalytic Materials: Metal Carbides of the Group VI B Elements. In Stud. Surf. Sci. Catal., Delmon B., Grange P., Jacobs P., Poncelet G., Eds.; Vol. 3; Elsevier, 1979; pp 627–639.

[ref66] Shetty S.; Jansen A. P. J.; van Santen R. A. Direct versus Hydrogen-Assisted CO Dissociation. J. Am. Chem. Soc. 2009, 131, 12874–12875. 10.1021/ja9044482. 19691313

[ref67] Shetty S.; van Santen R. A. CO Dissociation on Ru and Co Surfaces: The Initial Step in the Fischer–Tropsch Synthesis. Catal. Today 2011, 171, 168–173. 10.1016/j.cattod.2011.04.006.

[ref68] Loveless B. T.; Buda C.; Neurock M.; Iglesia E. CO Chemisorption and Dissociation at High Coverages during CO Hydrogenation on Ru Catalysts. J. Am. Chem. Soc. 2013, 135, 6107–6121. 10.1021/ja311848e. 23480097

[ref69] Qi K.-Z.; Wang G.-C.; Zheng W.-J. A First-Principles Study of CO Hydrogenation into Methane on Molybdenum Carbides Catalysts. Surf. Sci. 2013, 614, 53–63. 10.1016/j.susc.2013.04.001.

[ref70] Dang Y.; Li S. Catalytic Mechanism and Selectivity Prediction for syngas Conversion over Pure and K-promoted Mo_2_C Catalysts. Appl. Catal. A: Gen. 2021, 610, 117945 10.1016/j.apcata.2020.117945.

[ref71] Sullivan M. M.; Held J. T.; Bhan A. Structure and Site Evolution of Molybdenum Carbide Catalysts upon Exposure to Oxygen. J. Catal. 2015, 326, 82–91. 10.1016/j.jcat.2015.03.011.

[ref72] Lee W.-S.; Kumar A.; Wang Z.; Bhan A. Chemical Titration and Transient Kinetic Studies of Site Requirements in Mo_2_C-Catalyzed Vapor Phase Anisole hydrodeoxygenation. ACS Catal. 2015, 5, 4104–4114. 10.1021/acscatal.5b00713.

[ref73] Kumar A.; Bhan A. Oxygen Content as a Variable to Control Product Selectivity in Hydrodeoxygenation Reactions on Molybdenum Carbide Catalysts. Chem. Eng. Sci. 2019, 197, 371–378. 10.1016/j.ces.2018.12.027.

[ref74] Liu N.; Rykov S. A.; Chen J. G. A Comparative Surface Science Study of Carbide and Oxycarbide: the Effect of Oxygen Modification on the Surface Reactivity of C/W(111). Surf. Sci. 2001, 487, 107–117. 10.1016/S0039-6028(01)01070-6.

