# Supplementary material for: The Impact of Oxygen Surface Coverage and Carbidic Carbon on the Activity and Selectivity of Two-Dimensional Molybdenum Carbide (2D-Mo2C) in Fischer–Tropsch Synthesis
Source: ACS Catal. 2024 Jan 19;14(3):1834–45. doi: 10.1021/acscatal.3c03956 (PMC10845113; doi:10.1021/acscatal.3c03956)
Supplement: Supplementary file 1 — cs3c03956_si_001.pdf [file cs3c03956_si_001.pdf]

Supporting Information

The Impact of Oxygen Surface Coverage and Carbidic Carbon on the Activity and Selectivity of Two-Dimensional Molybdenum Carbide (2D-Mo_2C) in Fischer-Tropsch Synthesis

Evgenia Kountoupi[1], Alan J. Barrios[2,3], Zixuan Chen[1], Christoph R. Müller[1], Vitaly V. Ordomsky[2*], Aleix Comas-Vives[4,5*] and Alexey Fedorov[1*]

[1] Department of Mechanical and Process Engineering, ETH Zürich, CH-8092 Zürich, Switzerland

[2] University of Lille, CNRS, Centrale Lille, University of Artois, UMR 8181 − UCCS − Unité de Catalyse et Chimie du Solide, Lille F-59000, France

[3] Laboratory for Chemical Technology, Department of Materials, Textiles and Chemical Engineering, Ghent University, Ghent B-9052, Belgium

[4] Institute of Materials Chemistry, Technische Universität Wien, 1060 Vienna, Austria

[5] Departament de Química, Universitat Autònoma de Barcelona, 08193 Cerdanyola del Vallès, Catalònia, Spain

E-mail:

vitaly.ordomsky@univ-lille.fr

aleix.comas@tuwien.ac.at, aleix.comas@uab.cat

fedoroal@ethz.ch

List of Figures

Figure S1. XPS survey spectrum of Mo_2CT_x..6
Figure S2. Full range unprocessed Raman spectra of Mo_2CT_x, $Mo_2CT_{x\text{–}400}$, and $Mo_2CT_{x\text{–}500}$ along with the blank measurements of the empty glass slide and the 1 mm quartz capillary. ..7
Figure S3. (a) O 1s (b) F 1s and (c) C 1s XPS spectra of Mo_2CT_x along with the (d) atomic ratio between the oxo, hydroxy and fluoro T_x groups and the carbidic carbon........................8
Figure S4. Fractions of the fitted Mo 3d XPS states of the fresh and activated catalysts. 8
Figure S5. TPR of Mo_2CT_x using 90% H_2 in N_2 followed by MS. 9
Figure S6. (a)^{1}H NMR analysis, (b) GC-MS data (c) Mass spectrum for retention time 11 min (top) of the of the liquid fraction collected during the catalytic testing of $Mo_2CT_{x\text{–}400}$. The reference pattern for tridecane (NIST database) is shown at the bottom part of panel (c). 9
Figure S7. (a) Conversion of CO with time on stream and (b) selectivity to the gas-phase products obtained from $Mo_2CT_{x\text{–}400}$ (330 °C, 25 bar) in a reactor with i.d. = 2 mm..............10
Figure S8. (a) TPR of β-Mo_2C with 5% H_2 in Ar followed by MS. (b) XRD patter of β-Mo_2C after the TPR experiment. 12
Figure S9. (a) Integrated areas of the MS peaks obtained in the dynamic CO chemisorption measurement of β-$Mo_2C_{(500)}$ and β-$Mo_2C_{(400)}$ at −50 °C and (b) CO TPD from β-$Mo_2C_{(400)}$ and β-$Mo_2C_{(500)}$...13
Figure S10. Integrated areas of the MS peaks obtained in the dynamic CO chemisorption measurement of $Mo_2CT_{x\text{–}500}$ and $Mo_2CT_{x\text{–}400}$ (a) at −30 °C and (b) at 30 °C. 13
Figure S11. Conversion of CO obtained using $Mo_2CT_{x\text{–}500}$ and $Mo_2CT_{x\text{–}400}$ (330 °C, 25 bar) in a reactor with i.d. = 9.1 mm. 14
Figure S12. Atomic ratio between molybdenum and carbidic carbon for Mo_2CT_x, $Mo_2CT_{x\text{–}400}$, $Mo_2CT_{x\text{–}400\text{-}TOS2h}$, $Mo_2CT_{x\text{–}500}$ and $Mo_2CT_{x\text{–}500\text{-}TOS2h}$. 15
Figure S13. SEM images of (a) $Mo_2CT_{x\text{–}400}$, (b) $Mo_2CT_{x\text{–}500}$, (c) $Mo_2CT_{x\text{–}400\text{–}TOS2h}$ and (d) $Mo_2CT_{x\text{–}500\text{–}TOS2h}$ after their exposure to air. 16
Figure S14. XRD patterns of $Mo_2CT_{x\text{–}400}$ and $Mo_2CT_{x\text{–}500}$ after 2 h of TOS at 330 °C (25 bar). 17
Figure S15. Temperature programmed reduction experiments under 5% H_2 in Ar (black trace) and 5% CO in He (red trace)..17
Figure S16. (a) Generation of a DFT model of 2D-Mo_2C. (b) Top view, side view and parameters of the unit cell. Cyan and grey spheres indicate Mo and C atoms, respectively. ..18
Figure S17. On-top sites and bridge sites. 19
Figure S18. Vicinal three-fold hollow sites and intersecting Mo atom..................................19
Figure S19. Energy profile for the (a) H_2-assisted and (b) direct CO dissociation pathways. Energies are calculated with respect to the initial reactants (a) 1 CO and 0.5 H_2 (b) 1 CO. Intermediate and transition states are shown in (c). The respective G_{rel} (eV) values are given in parenthesis..20
Figure S20. Energy profile for (a) water formation via OH hydrogenation and condensation of two OH sites, yielding H_2O^* and O^* (energies are calculated with respect to 2 CO and 1 H_2), and (b) CO_2 formation (energies are calculated with respect to 2 CO). Snapshots of the

intermediate and transition states are shown in (c). The respective G_{rel} (eV) values are given in parenthesis..21
Figure S21. Top view (3 snapshots on the left) and side view (3 snapshots on the right) of initial (left), transition (middle), and final (right) states associated with steps R1-R9 presented in Table S4. 23
Figure S22. Top view (3 snapshots on the left) and side view (3 snapshots on the right) of initial (left), transition (middle), and final (right) states associated with steps R10-R18 presented in Table S4. 24
Figure S23. Top view (3 snapshots on the left) and side view (3 snapshots on the right) of initial (left), transition (middle), and final (right) states associated with steps R19-R29 presented in Table S4. 25
Figure S24. Top view (3 snapshots on the left) and side view (3 snapshots on the right) of initial (left), transition (middle), and final (right) states associated with steps R30-R35 presented in Table S4. 26
Figure S25. Gibbs energy barriers plotted against Gibbs reaction energies for (a) C–C coupling and (b) hydrogenation elementary steps (R1-R34). The arrow shows the direction of increase with increasing value of index x. 26
Figure S26. (a) Energy profile for ethane formation on a 2D-Mo_2C slab including CH–CH coupling steps via the formation of ethylene (black trace) as key intermediate. (b) Snapshots of selected intermediates and transition states. 27
Figure S27. Energy profile for ethane formation on a 2D-Mo_2C slab including CH–CH coupling steps, via the H-assisted transformation of acetylene to ethylidyne. 28
Figure S28. Energy profile for methane formation. 29
Figure S29. (a) Activation barriers and (b) reaction energies of selected elementary steps in the pathway of CO hydrogenation towards ethane on 2D-Mo_2C-0.67 O ML (orange bars) compared to 2D-Mo_2C (green bars). 31

List of Tables

Table S1. Catalytic results for tests using β-$Mo_2C_{(400)}$, $Mo_2CT_{x\text{–}400}$ and $Mo_2CT_{x\text{–}500}$ (T = 330 °C, H_2/CO = 2, P = 25 bar, GHSV = 5.1 L g^{-1} h^{-1}). Total selectivity (i.e., including CO_2) is shown. 11
Table S2. Gravimetric rate of CO consumption and CH_4 and C_{5+} production for β-$Mo_2C_{(400)}$, $Mo_2CT_{x\text{–}500}$, as well as for $Mo_2CT_{x\text{–}400\text{-}TOS1h}$ and $Mo_2CT_{x\text{–}400\text{-}TOS8h}$. 12
Table S3. CO uptake capacities determined in pulse chemisorption measurements. 14
Table S4. Activation barriers, reaction energies and reverse barriers of all elementary steps examined in the pathways of CO hydrogenation towards ethane on 2D-Mo_2C. 22
Table S5. Activation barriers, reaction energies and reverse barriers of selected elementary steps in the pathway of CO hydrogenation to ethane on the 2D-Mo_2C-0.67 O ML model.....30
Table S6. Adsorption energy of atomic adsorbates C^*, O^*, H^* on Ἡ$_{Mo}$, Ἡ$_C$ and on-top sites. 36
Table S7. XPS fitting parameters for fresh and activated (TOS = 2 h) catalysts for the Mo region. 36
Table S8. XPS fitting parameters for fresh and activated (TOS = 2 h) catalysts in the C region. 37

Material Synthesis

A reported procedure for the synthesis of Mo_2CT_x was used.[1, 2] It is provided below for completeness.

In a typical experiment, powder of β-Mo_2C (3 g, Sigma-Aldrich, 99.5 %) was carefully mixed with an excess of metallic Ga (9 g, Sigma-Aldrich, 99.9995% trace metals basis). The resulting gray paste was placed into a quartz ampule and the tube was flame-sealed under ca. 10^{-5} mbar. The sealed ampule was then heated in a furnace to 850 °C with a heating rate of 10 °C min^{-1} and held at this temperature for 48 h before cooling down to room temperature. To remove excess Ga, the resulting material was washed in HCl (30 mL of a 12 M technical grade HCl, Fisher Scientific) for 48 h at room temperature under magnetic stirring. The reaction mixture was transferred into a centrifuge tube and washed repeatedly with DI water until a pH of ca. 6 was reached. The resulting Mo_2Ga_2C solid was dried in air at 80 °C for 12 h. To prepare Mo_2CT_x, 700 mg of Mo_2Ga_2C were placed in a 200 mL Teflon-lined autoclave together with 40 ml of concentrated aqueous HF (14 M). The sealed autoclave was kept in a sand bath and heated for 1 week at 140 °C under stirring (400 rpm, Teflon coated magnet). Once the suspension was cooled down, it was transferred into a centrifuge tube and washed repeatedly with DI water at 3000 rpm for 3 min until a pH of ca. 6 was reached. The collected solid was dried in air at 80 °C for 12 h.

Characterization Details

Powder X-ray diffraction (XRD) data was collected on a PANalytical Empyrean X-ray diffractometer. The diffractometer uses a Bragg–Brentano HD mirror and operates at 45 kV and 40 mA using Cu Kα radiation (1.5418 Å). The materials were scanned in the 2θ range of 5–70° using a step size of 0.0167°. The scan time per step was 50 s.

X-ray photoelectron spectroscopy (XPS) measurements were conducted on a Sigma 2 instrument (Thermo Fisher Scientific) equipped with a UHV chamber (non-monochromatic 200 W Al Kα source, a hemispherical analyzer, and a seven-channel electron multiplier). The analyzer-to-source angle was 50° and the emission angle was 0°. A pass energy of 50 and 25 eV was set for the survey and the narrow scans, respectively. Referencing of the binding energy scale was performed by setting the C *1s* peak of adventitious carbon to 284.8 eV to eliminate specimen charging effects. XPS spectra of the H_2-pretreated and activated catalysts were recorded using a custom-made air-tight cell that allowed to transfer air-sensitive materials between a glovebox and the UHV chamber while avoiding exposure to air. Data analysis was performed with the CasaXPS software (Version 2.3.25PR1.0). Background subtraction was performed using Shirley background.[3] The Scofield sensitivity factors of were used to calculate the atomic composition.[4]

Raman spectroscopy was performed in a DXR 2 Raman spectrometer (Thermo Fisher) using a 780 nm excitation laser with a laser power of 3 mW, a 10× long path objective and a 50 μm slit aperture. For each measured spot, 25 scans were collected (5 sec $scan^{-1}$). For air-exposed samples, five spots per sample were collected and averaged. Activated samples were loaded into quartz capillaries (i.d. 1 mm) inside a glovebox, sealed and measured without air exposure. For the activated materials, 10 spots were measured per specimen and averaged.

CO chemisorption experiments were performed with a Micromeritics AutoChem II 2920 analyzer equipped with a thermal conductivity detector (TCD) and a mass spectrometer (MS). For each experiment, the material was loaded into a U-shaped quartz reactor tube and fixed between two quartz wool plugs. The flow of gas was 50 ml min^{-1}. The activated materials were prepared *in situ* as described in the materials synthesis section (see the main text). After the pretreatment, the gas atmosphere was switched to He and the specimen was purged for 30 min at the selected pretreatment temperature. The temperature was then decreased to −30 °C or 30 °C. The pulsing of CO (5% v/v in He) was started once

the baseline had been stabilized. During the analysis, the gas was flowed through a cold trap (isopropanol slurry, −90 °C) that served as a short delay path to counteract the flow disturbance caused by the CO injections. The mass of the used specimen were 29 mg and 79 mg for (*in situ* prepared) $Mo_2CT_{x\text{–}500}$ and $Mo_2CT_{x\text{–}400}$, respectively for the experiments performed at −30 °C. For the experiments at 30 °C 14 mg of $Mo_2CT_{x\text{–}500}$ and 40 mg of $Mo_2CT_{x\text{–}400}$ were used. For chemisorption experiments on β-Mo_2C, a sample mass of 500 mg were used, irrespective of the reduction temperature. These amounts were chosen to detect changes with sufficient signal-to-noise ratio in the MS signal.

Temperature programmed desorption (TPD) experiments were performed on β-$Mo_2C_{(400)}$ and β-$Mo_2C_{(500)}$. Prior to desorption, the material was saturated with CO. In these experiments, we performed CO chemisorption at −50 °C following the process described above. Once saturation was achieved, the temperature was increased to 300 °C (50 °C·min^{-1}) under He and the effluent gas was monitored with an MS detector.

H_2 and CO TPR experiments using Mo_2CT_x were performed in the same instrument (Micromeritics AutoChem II 2920). In a typical experiment, 40 mg of the specimen was loaded in the U-shaped reactor. The specimen was heated under nitrogen to 110 °C (5 °C·min^{-1}) and held at that temperature for 1 h to remove physisorbed water. Subsequently, the temperature was decreased to 40 °C and the gas was switched to the analysis gas. For H_2 TPR, the TCD signal was monitored up to 700 °C and for CO TPR up to 900 °C. Additional H_2 TPR experiments using β-Mo_2C (500 mg) and Mo_2CT_x (20 mg) were performed in a quartz flow reactor while the effluent gas was monitored with an MS detector.

Details of Catalytic Tests

The equations used for product quantification are provided below (Eq. I-III).

CO conversion was determined using equation I, where F is the molar flow of CO determined using 5 % N_2 as internal standard:

$$X_{\mathrm{CO}} = 1 - \frac{F_{\mathrm{CO,out}}}{F_{\mathrm{CO,in}}} * 100\ \%\ (\mathrm{I})$$

The selectivity to the products was determined via equation II, where F_i is the molar flow of the product *i*:

$$S_i = \frac{F_i}{F_{\mathrm{CO,in}} - F_{\mathrm{CO,out}}} * 100\%\ (\mathrm{II})$$

The carbon balance (ε_c) was higher than 90 % in the catalytic tests and was calculated as:

$$\varepsilon_c = \frac{\sum_i F_i + F_{\mathrm{CO,out}}}{F_{\mathrm{CO,in}}} * 100\%\ (\mathrm{III})$$

Computational Details

The interactions between valence electrons and cores were described with the projector-augmented wave (PAW) method while the electronic wave functions were expanded as a discrete plane wave (PW) basis set.[5, 6] All calculations utilized a PW energy cutoff of 500 eV. The exchange and correlation

energy were determined via the generalized gradient approximation (GGA), with Bayesian error estimation functionals (BEEFs) including non-local van der Waals correlations.[7] For the correlation part of the exchange correlation functional, the formula according to Vosko, Wilk and Nusair was used.[8] 3 × 3 × 1 Monkhorst–Pack grids were used to sample the Brillouin zone.[9] Thermal broadening of the molecular orbital occupancy was performed with Gaussian smearing (with a smearing width of 0.05 eV).[10] Self-consistent field (SCF) calculations of the electronic structure were considered converged once the electronic energy change between two consecutive steps was less than 10^{-5} eV. All geometries were optimized until the forces acting on each atom were converged below 0.1 eV $Å^{-1}$. Errors introduced from the periodic boundary conditions were accounted for by dipole corrections in the z-direction and the center of charge was set at the center in the unit cell. The reference energy of isolated molecules was determined by a Γ-point calculation during which each species was placed in a 15 × 15.5 × 16 Å box. The climbing image nudge elastic band (CI-NEB) method with eight intermediate images was used to identify transition states (TS) that were confirmed by frequency analysis.[11, 12] Normal vibration modes of adsorbed species were calculated by diagonalization of the Hessian matrix, obtained using a central finite difference approximation with displacements equal to 0.015 Å in the direction of each Cartesian coordinate. All Mo and C atoms from the 2D carbide model were fixed during the frequency calculations of the adsorbed species.

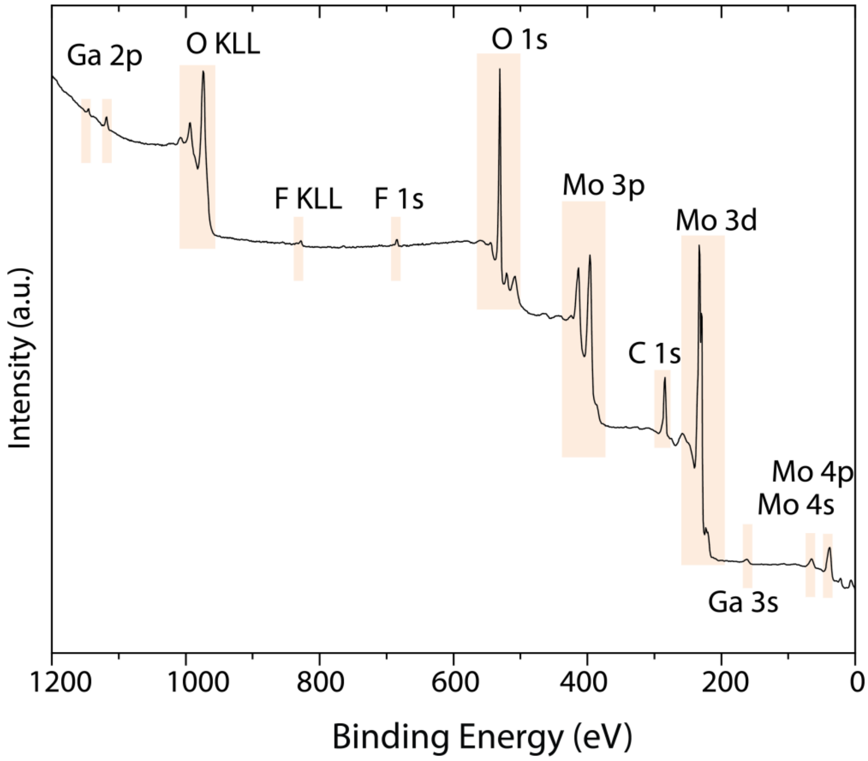

Figure S1. XPS survey spectrum of Mo_2CT_x.

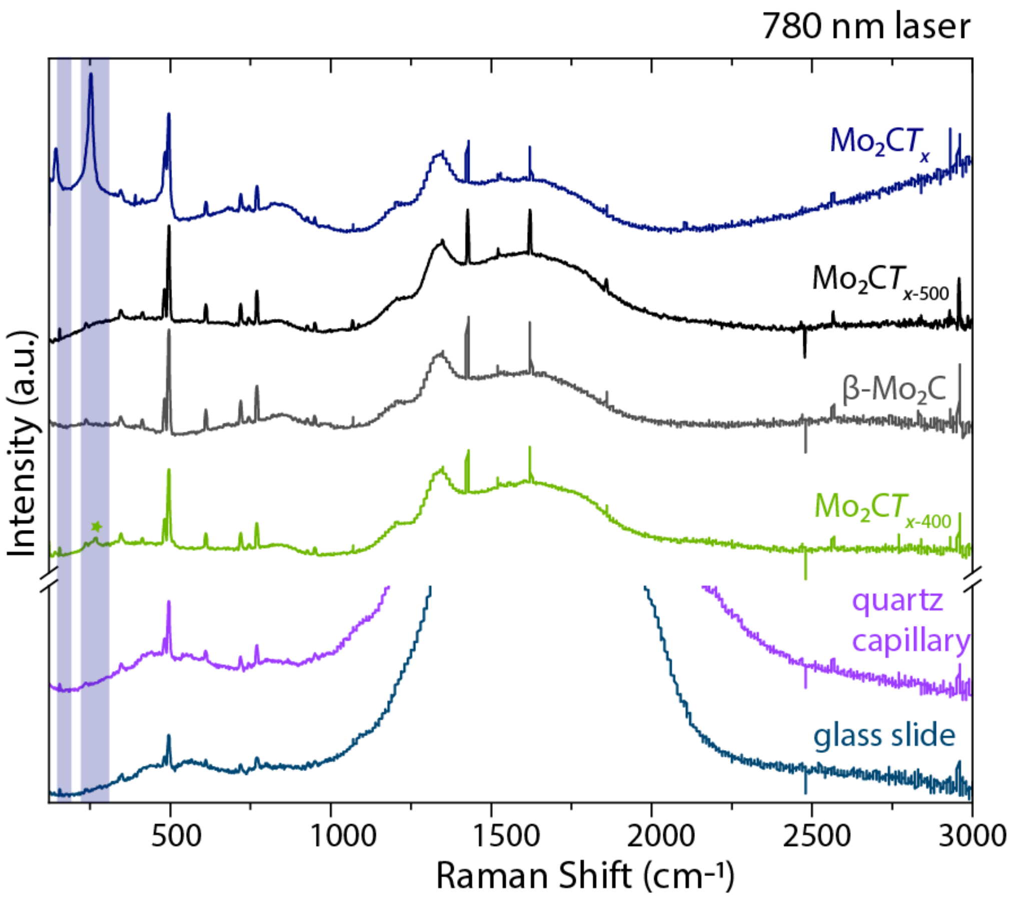

Figure S2. Full range unprocessed Raman spectra of Mo_2CT_x, $Mo_2CT_{x\text{–}400}$, and $Mo_2CT_{x\text{–}500}$ along with the blank measurements of the empty glass slide and the 1 mm quartz capillary.

Note that two bands around 488 cm^{-1} are due to the glass slide/quartz capillary. In addition, the glass slide/quartz capillary show a very intense fluorescence band between ca. 1000 and 2500 cm^{-1}.

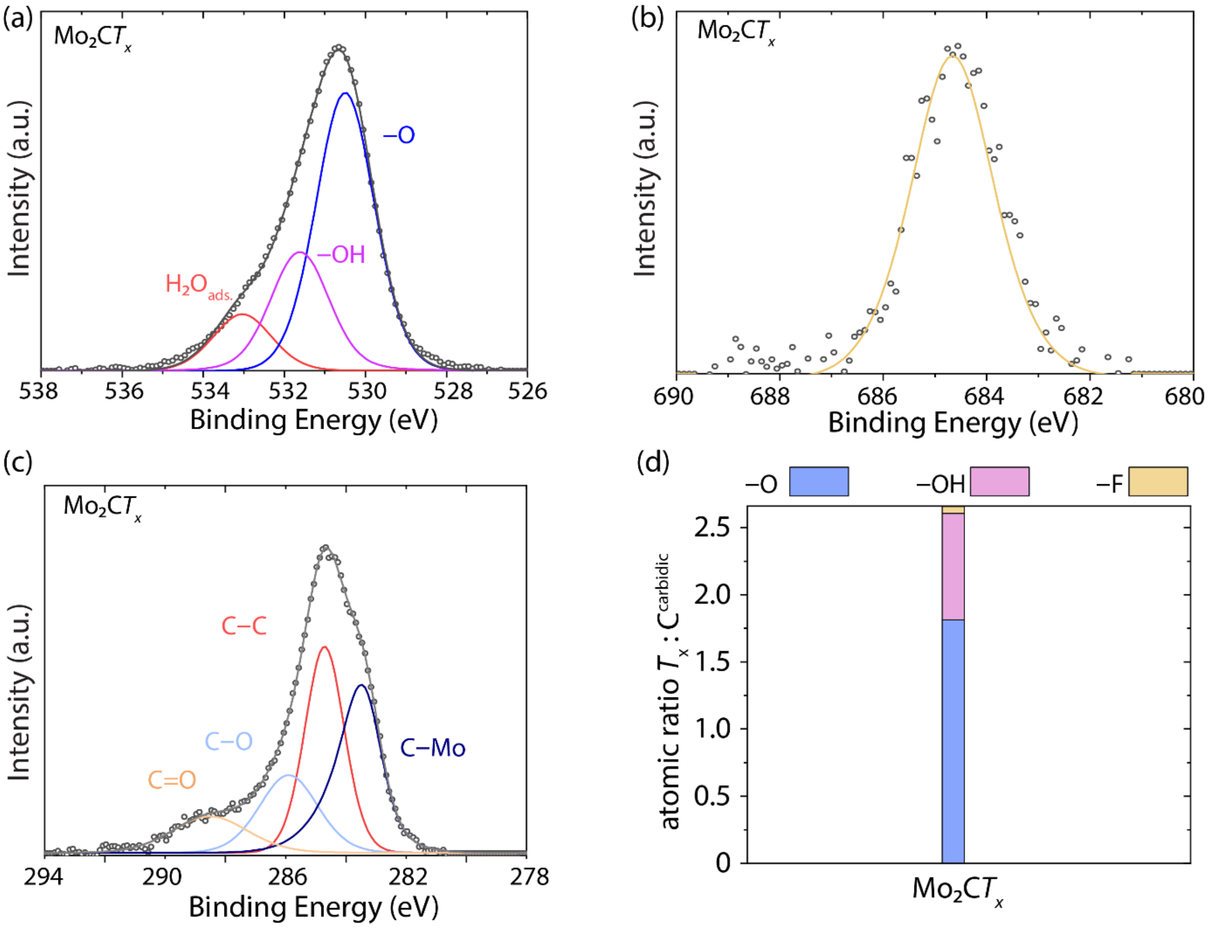

Figure S3. (a) O 1*s* (b) F 1*s* and (c) C 1*s* XPS spectra of Mo_2CT_x along with the (d) atomic ratio between the oxo, hydroxy and fluoro T_x groups and the carbidic carbon.

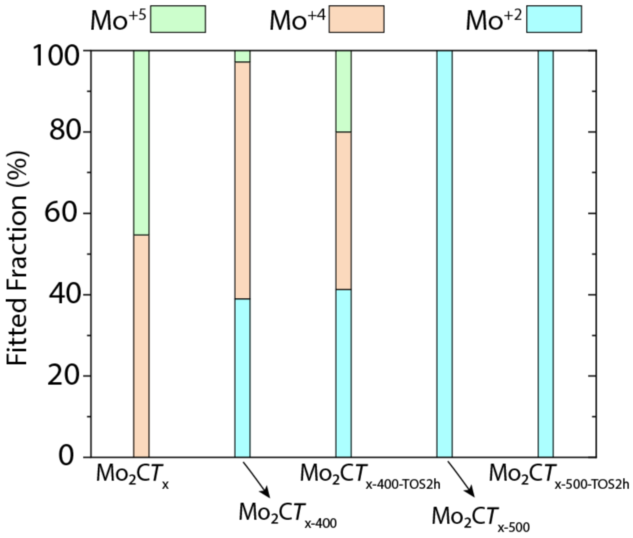

Figure S4. Fractions of the fitted Mo 3*d* XPS states of the fresh and activated catalysts.

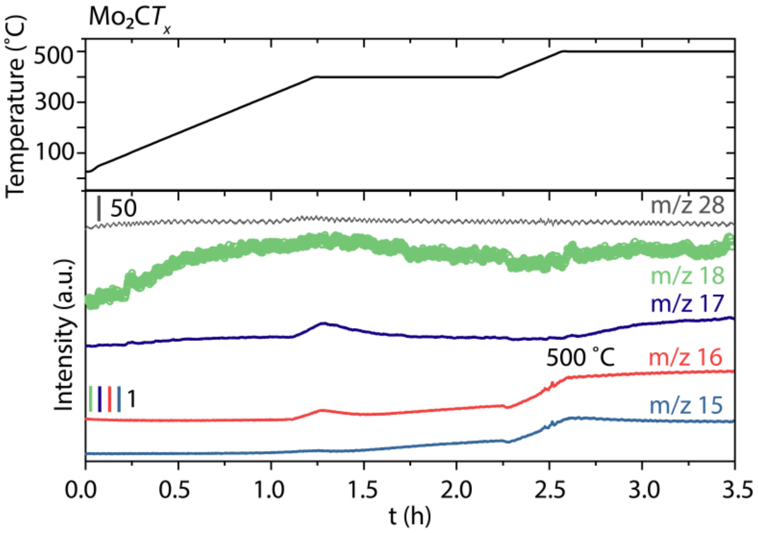

Figure S5. TPR of Mo_2CT_x using 90% H_2 in N_2 followed by MS.

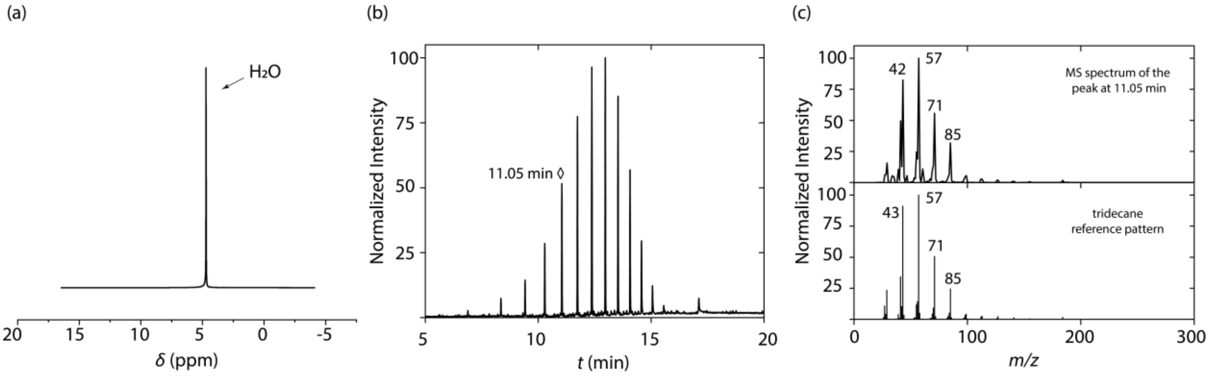

Figure S6. (a)^{1}H NMR analysis, (b) GC-MS data (c) Mass spectrum for retention time 11 min (top) of the of the liquid fraction collected during the catalytic testing of Mo_2CT_{x-400}. The reference pattern for tridecane (NIST database) is shown at the bottom part of panel (c).

The liquid product was analyzed by ^{1}H nuclear magnetic resonance spectroscopy by mixing 0.5 mL of the filtered liquid fraction (collected during the experiment presented in Figure 2c-d of the main text) with a 0.1 mL DMSO/D_2O solution.

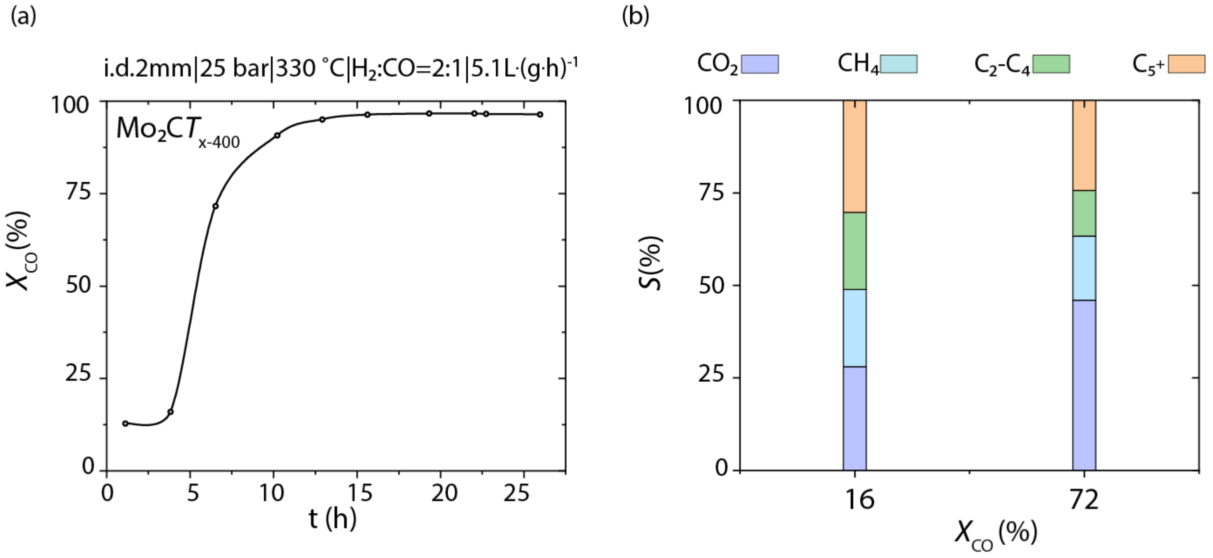

Figure S7. (a) Conversion of CO with time on stream and (b) selectivity to the gas-phase products obtained from Mo_2CT_{x-400} (330 °C, 25 bar) in a reactor with i.d. = 2 mm.

Table S1. Catalytic results for tests using β-$Mo_2C_{(400)}$, $Mo_2CT_{x\text{–}400}$ and $Mo_2CT_{x\text{–}500}$ (T = 330 °C, H_2/CO = 2, P = 25 bar, GHSV = 5.1 L g^{-1} h^{-1}). Total selectivity (i.e., including CO_2) is shown.

Entry	Catalyst	TOS (h)	X_{CO} (%)	$S(CO_2)$ (%)	$S(CH_4)$ (%)	$S(C_2{=}C_4)$ (%)	$S(C_2\text{–}C_4)$ (%)	$S(C_5^+)$ (%)
1	β-$Mo_2C_{(400)}$	1	2	15	44	11	20	10
2	$Mo_2CT_{x\text{–}400}$	3.8	16	28	21	–	21	30
3	$Mo_2CT_{x\text{–}400}$	6.5	72	46	17	–	12	24
4	$Mo_2CT_{x\text{–}400}$	8	88	50	13	–	10	27
5	$Mo_2CT_{x\text{–}500}$	0.9	92	49	29	–	20	2
6	$Mo_2CT_{x\text{–}500}$	1.9	93	48	31	–	20	1
7	$Mo_2CT_{x\text{–}500}$	2.8	94	46	32	–	21	1
8	$Mo_2CT_{x\text{–}500}$	3.7	95	48	31	–	19	1
9	$Mo_2CT_{x\text{–}500}$	4.7	96	46	33	–	20	1
10	$Mo_2CT_{x\text{–}500}$	5.6	96	48	32	–	19	1
11	$Mo_2CT_{x\text{–}500}$	6.5	95	46	33	–	20	1
12	$Mo_2CT_{x\text{–}500}$	7.4	93	48	32	–	19	1
13	$Mo_2CT_{x\text{–}500}$	8.4	95	48	32	–	19	1
14	$Mo_2CT_{x\text{–}500}$	9.3	94	47	32	–	20	1
15	$Mo_2CT_{x\text{–}500}$	10.2	96	47	32	–	20	1
16	$Mo_2CT_{x\text{–}500}$	11.2	95	50	31	–	19	1
17	$Mo_2CT_{x\text{–}500}$	12.1	96	48	32	–	20	1
18	$Mo_2CT_{x\text{–}500}$	13.0	95	46	33	–	20	1
19	$Mo_2CT_{x\text{–}500}$	14.0	93	48	32	–	19	1
20	$Mo_2CT_{x\text{–}500}$	14.9	95	48	32	–	19	1
21	$Mo_2CT_{x\text{–}500}$	15.8	94	47	32	–	20	1
22	$Mo_2CT_{x\text{–}500}$	16.7	91	47	32	–	20	1
23	$Mo_2CT_{x\text{–}500}$	17.7	93	50	31	–	19	1
24	$Mo_2CT_{x\text{–}500}$	18.6	95	46	33	–	20	1
25	$Mo_2CT_{x\text{–}500}$	19.5	95	49	31	–	19	1
26	$Mo_2CT_{x\text{–}500}$	20.5	94	51	30	–	18	1

"–" denotes not detected or less than 0.5%.

Data in entry 1 was acquired in an experiment presented in Figure 2a. Data in entries 2–3 were acquired in an experiment presented in Figure S7. Data in entry 4 was acquired in the experiment presented in Figure 2c. Data in entries 5–26 were acquired in the experiment presented in Figure 2b.

Table S2. Gravimetric rate of CO consumption and CH_4 and C_{5+} production for β-$Mo_2C_{(400)}$, $Mo_2CT_{x\text{–}500}$, as well as for $Mo_2CT_{x\text{–}400\text{-TOS1h}}$ and $Mo_2CT_{x\text{–}400\text{-TOS8h}}$.

Entry	Catalyst	r_{CO} (mmol g_{cat} h^{-1})	$r_{C_{5+}}$ (mmol g_{cat} h^{-1})	r_{CH_4} (mmol g_{cat} h^{-1})
1	β-$Mo_2C_{(400)}$	1.3	0.1	2.2
2	$Mo_2CT_{x\text{–}400\text{-TOS1h}}$	10.6	3.2	3.4
3	$Mo_2CT_{x\text{–}400\text{-TOS8h}}$	58.1	15.7	7.6
4	$Mo_2CT_{x\text{–}500}$	62.1	0.6	19.9

H_2-TPR-MS Results

The TPR experiment using β-Mo_2C was performed in a flow reactor with 5% H_2 in Ar and the off gas was monitored with a mass spectrometer. The MS spectrum features two peaks centred at ca. 377 °C and 400 °C. The peak at 377 °C is associated with the release of CO_2 while the peak at 400 °C is due to the release of H_2O (Figure S8a). The minor peak due to release of CO_2 maybe due to the decomposition of surface carbonate species. No metallic Mo or phases other than β-Mo_2C was identified by XRD in the post-experiment specimen (Figure S8b).

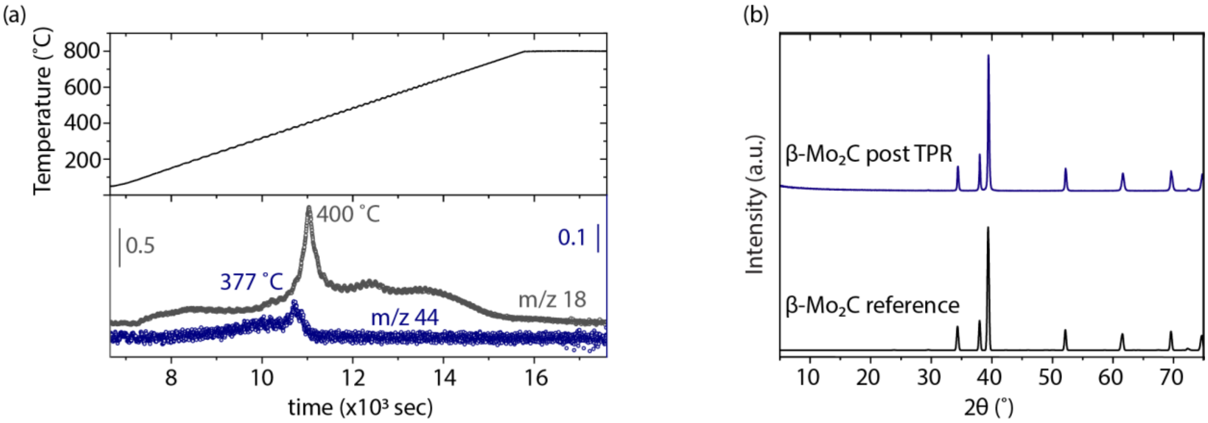

Figure S8. (a) TPR of β-Mo_2C with 5% H_2 in Ar followed by MS. (b) XRD patter of β-Mo_2C after the TPR experiment.

CO-TPD and CO Chemisorption Results

Chemisorption experiments using *in situ* prepared β-$Mo_2C_{(400)}$ and β-$Mo_2C_{(500)}$ show that such materials feature similar CO capacities at −50 °C, determined as 4 and 3 μmol g_{cat}^{-1}, respectively (Table **S3**). The adsorption isotherms are shown in Figure S9a. CO-TPD using β-$Mo_2C_{(500)}$ yields a broad desorption peak around 15 °C, in agreement with the value reported previously.[13] Yet this peak can be deconvolved into two components, one centered at −26 °C and another one centered at 15 °C (Figure S9b). Interestingly, β-$Mo_2C_{(400)}$ displays a symmetric desorption peak centered around 12 °C, fitted by a single component. This suggests that the strongest adsorption sites (represented by the component at −26 °C) are populated by remaining oxygen in β-$Mo_2C_{(400)}$.

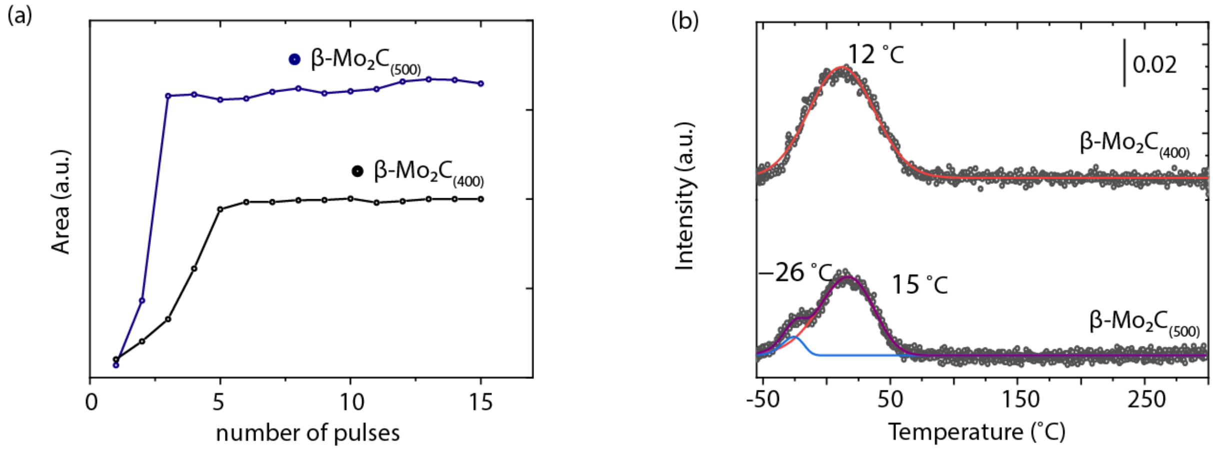

Figure S9. (a) Integrated areas of the MS peaks obtained in the dynamic CO chemisorption measurement of β-$Mo_2C_{(500)}$ and β-$Mo_2C_{(400)}$ at −50 °C and (b) CO TPD from β-$Mo_2C_{(400)}$ and β-$Mo_2C_{(500)}$.

Next, we performed CO chemisorption using $Mo_2CT_{x\text{–}400}$ and $Mo_2CT_{x\text{–}500}$ at −30 °C, a temperature slightly lower than the expected CO desorption temperature (estimated as the ascending inflection point of the desorption peak)[13]. The results are shown in Table **S3** and the adsorption isotherms are plotted in Figure S10a. Notably, this data demonstrates a higher CO capacity for $Mo_2CT_{x\text{–}400}$ relative to $Mo_2CT_{x\text{–}500}$ (entries 1 and 2, Table **S3**). To investigate the effect of diffusion limitations on the determined CO capacity values, we performed CO chemisorption on $Mo_2CT_{x\text{–}400}$ and $Mo_2CT_{x\text{–}500}$ catalysts at 30 °C (Figure S10b and Table **S3**). At this temperature, only a partial surface CO coverage is probed. The CO capacity of $Mo_2CT_{x\text{–}500}$ at 30 °C is ca. 7 times larger than that of $Mo_2CT_{x\text{–}400}$ (entries 3 and 4, Table **S3**).

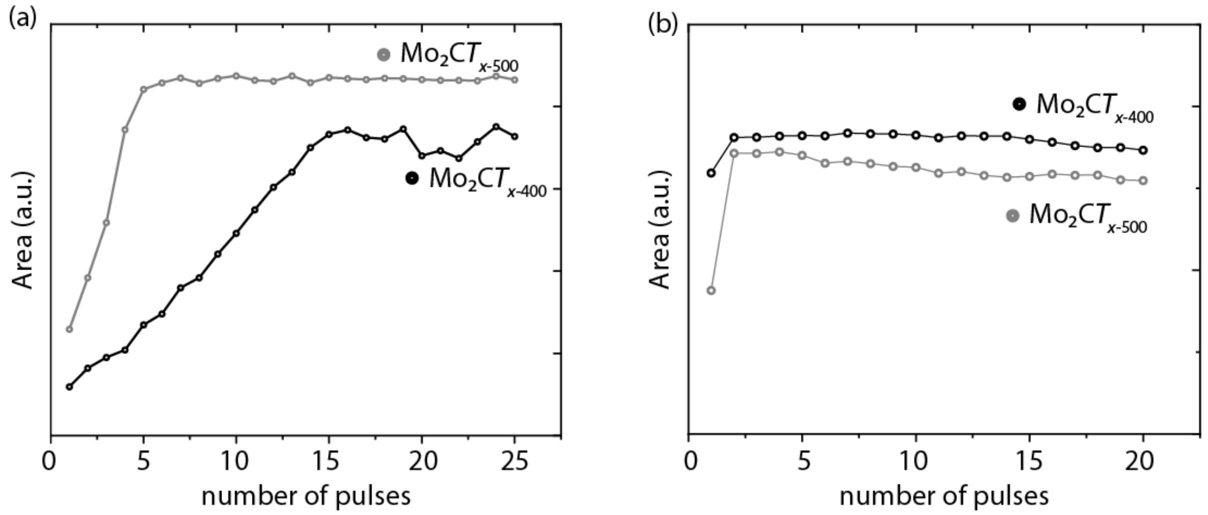

Figure S10. Integrated areas of the MS peaks obtained in the dynamic CO chemisorption measurement of $Mo_2CT_{x\text{–}500}$ and $Mo_2CT_{x\text{–}400}$ (a) at −30 °C and (b) at 30 °C.

Table S3. CO uptake capacities determined in pulse chemisorption measurements.

Entry	Material	CO capacity (μmol g_{cat}^{-1})	Experimental temperature (°C)
1	$Mo_2CT_{x\text{–}400}$	63	−30
2	$Mo_2CT_{x\text{–}500}$	47	−30
3	$Mo_2CT_{x\text{–}400}$	5	30
4	$Mo_2CT_{x\text{–}500}$	34	30
5	β-$Mo_2C_{(400)}$	4	−30
6	β-$Mo_2C_{(500)}$	3	−30
7	β-$Mo_2C_{(400)}$	2	30
8	β-$Mo_2C_{(500)}$	1	30

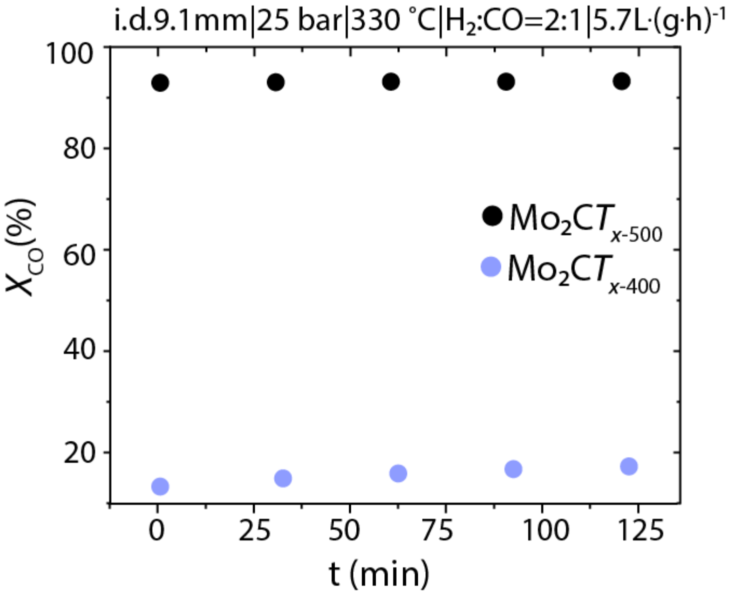

Figure S11. Conversion of CO obtained using $Mo_2CT_{x\text{–}500}$ and $Mo_2CT_{x\text{–}400}$ (330 °C, 25 bar) in a reactor with i.d. = 9.1 mm.

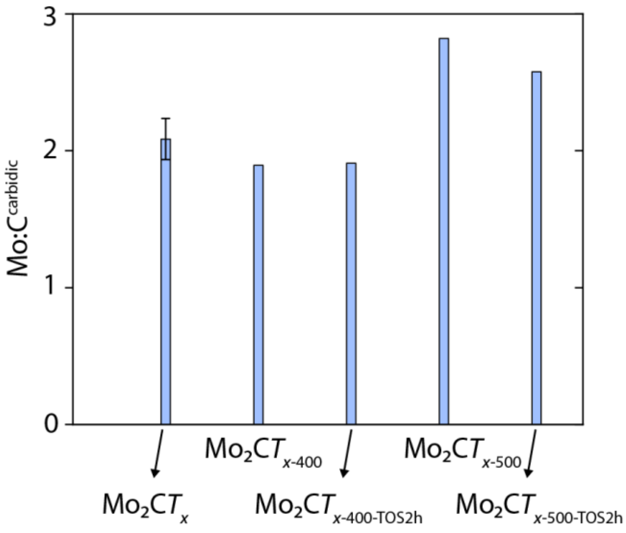

Figure S12. Atomic ratio between molybdenum and carbidic carbon for Mo_2CT_x, $Mo_2CT_{x\text{–}400}$, $Mo_2CT_{x\text{–}400\text{-TOS2h}}$, $Mo_2CT_{x\text{–}500}$ and $Mo_2CT_{x\text{–}500\text{-TOS2h}}$.

Estimated and BET-measured surface area of Mo_2CT_x.

The BET surface area of Mo_2CT_x has been measured at ca. 7 $m^2 \cdot g^{-1}$.[14]

Mo_2CT_x has the lattice parameters a = 2.8629 Å and c = 20.515 Å.[1]

The area of a hexagon is:

$$A = a^2 sin60° = 7.098\ \text{Å}^2 \cdot \text{Mo}^{-1}$$

Using the XPS-derived chemical composition of $Mo_2CO_{1.8}OH_{0.8}F_{0.1}$, we obtained a Mo content of ca. 75% w/w. The upper estimate for its surface area is:

$$SSA = \frac{7.098\ \text{Å}^2 \cdot \text{Mo atom}^{-1} \cdot 6.023 \cdot 10^{23}\ \text{Mo atom}\ \cdot \text{mol}_{\text{Mo}}{}^{-1}}{95.95\ \text{g}_{\text{Mo}} \cdot \text{mol}_{\text{Mo}}{}^{-1}} \cdot 0.75\ \text{g}_{\text{Mo}}\ \cdot \text{g}_{\text{cat}}{}^{-1}$$
$$\approx 2400\ \text{m}^2 \cdot \text{g}_{\text{cat}}{}^{-1}$$

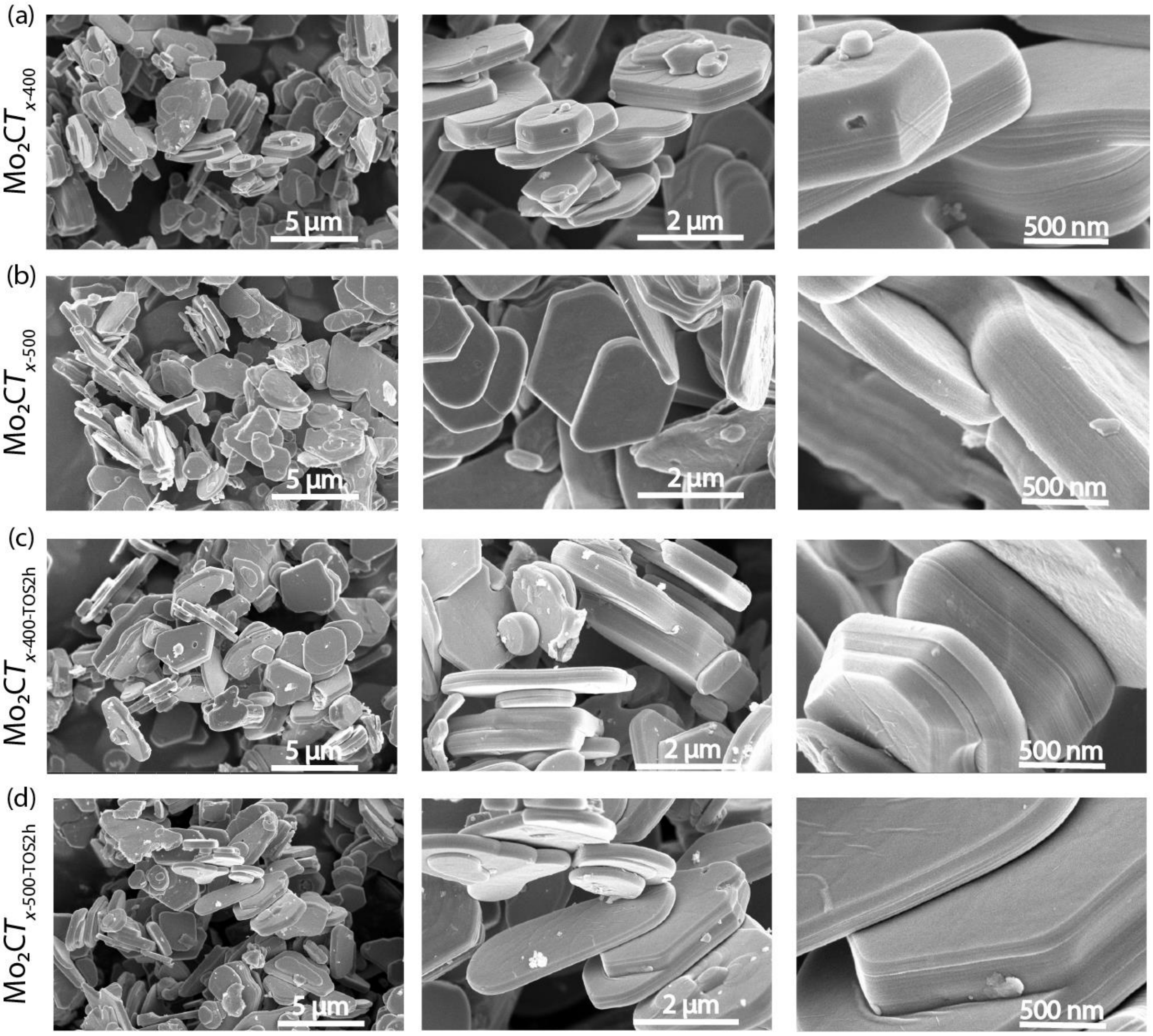

Figure S13. SEM images of (a) $Mo_2CT_{x\text{−}400}$, (b) $Mo_2CT_{x\text{−}500}$, (c) $Mo_2CT_{x\text{−}400\text{−}TOS2h}$ and (d) $Mo_2CT_{x\text{−}500\text{−}TOS2h}$ after their exposure to air.

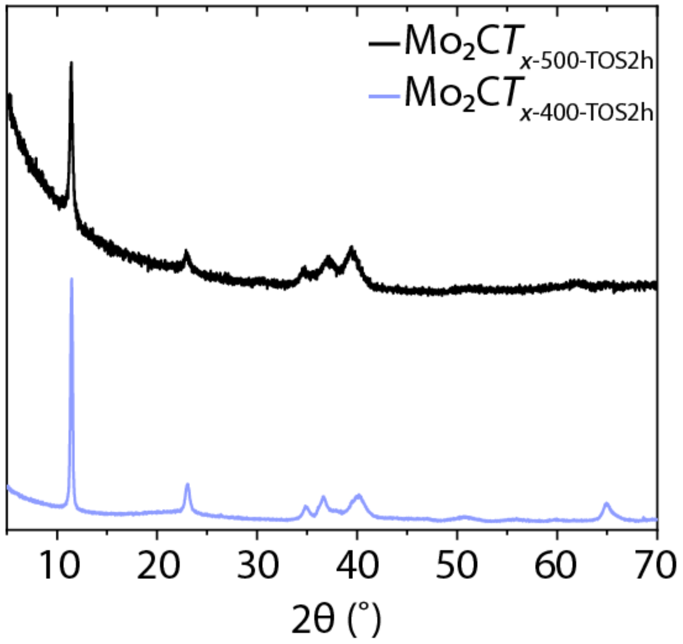

Figure S14. XRD patterns of $Mo_2CT_{x\text{–}400}$ and $Mo_2CT_{x\text{–}500}$ after 2 h of TOS at 330 °C (25 bar).

The drifting baseline towards the lower angles is due to the presence of quartz wool in the recovered catalysts.

To examine whether CO is a more efficient reducing agent compared to H_2, we compared H_2 and CO temperature programmed reduction experiments (Figure S15). Under 5% CO/He, the two reduction peaks centred at ca 400 °C and 730 °C appear at a temperature that is approximately 150 °C higher relative to 5% H_2/Ar (ca. 245 °C and 590 °C). These results suggest that an elevated pressure is necessary for the efficient defunctionalization of Mo_2CT_x when using CO or syngas (or the presence of $Mo_2CT_{x\text{–}400}$ in place of Mo_2CT_x is needed for the defunctionalization reaction).

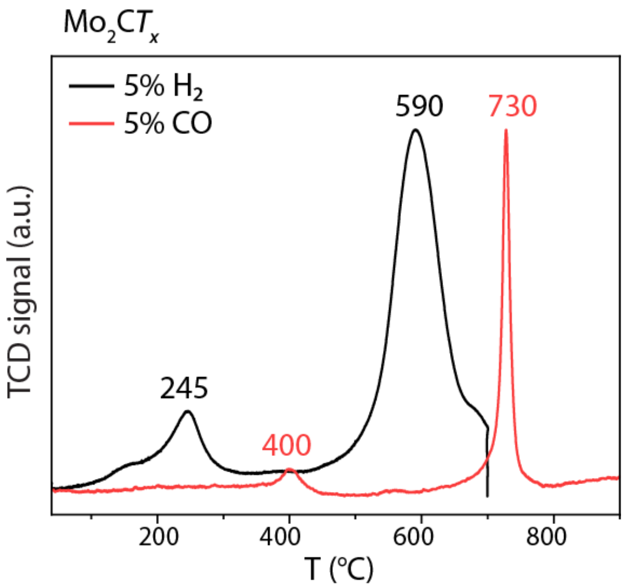

Figure S15. Temperature programmed reduction experiments under 5% H_2 in Ar (black trace) and 5% CO in He (red trace).

DFT Models

The (0001) facet of the 2D-Mo_2C model was constructed from the experimental structure of Mo_2Ga_2C as described in our previous report.[15, 16] This approach is reproduced here for the sake of completeness.

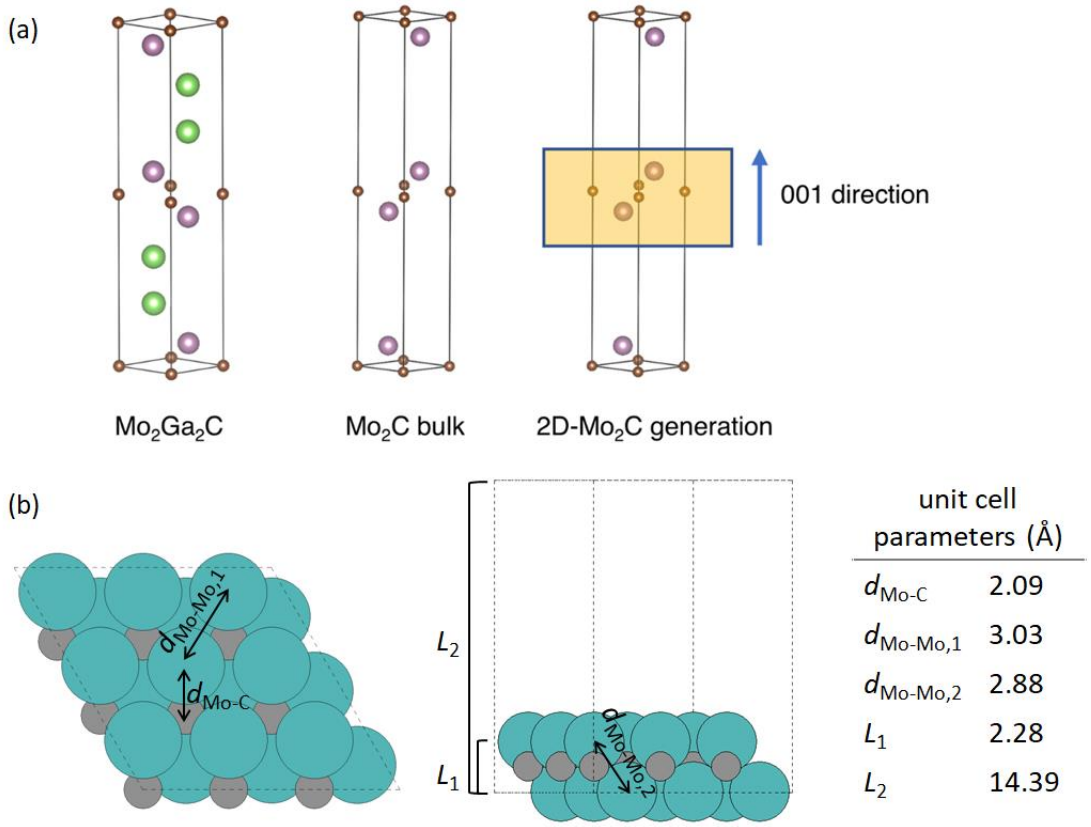

Figure S16. (a) Generation of a DFT model of 2D-Mo_2C. (b) Top view, side view and parameters of the unit cell. Cyan and grey spheres indicate Mo and C atoms, respectively.

Ga atoms (green) were removed from the structure of Mo_2Ga_2C to generate the bulk structure with a Mo_2C stoichiometry. The 2D-facet model of Mo_2C was obtained by selecting the inner fragment of the cell in the 001 direction (Figure S16) and performing a full geometry optimization of the resulting structure. A 3 × 3 unit cell, derived from the Mo_2C (0001) surface slab, was used for all further calculations.

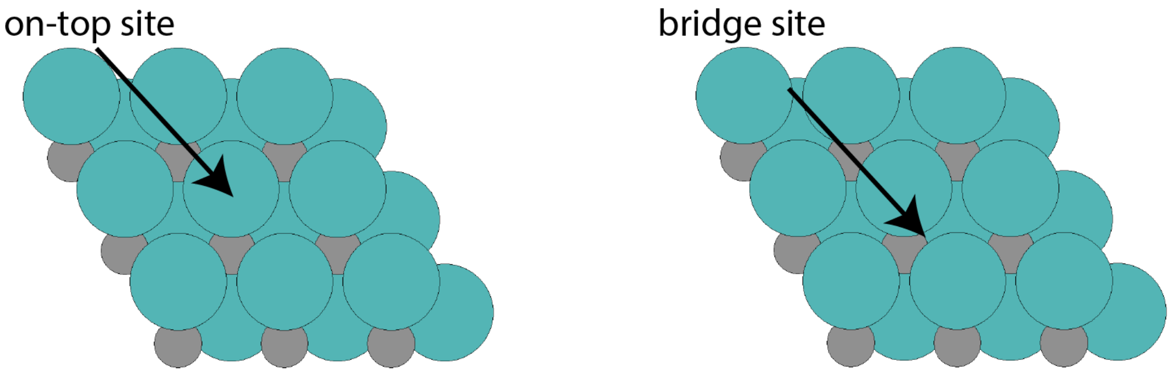

Figure S17. On-top sites and bridge sites.

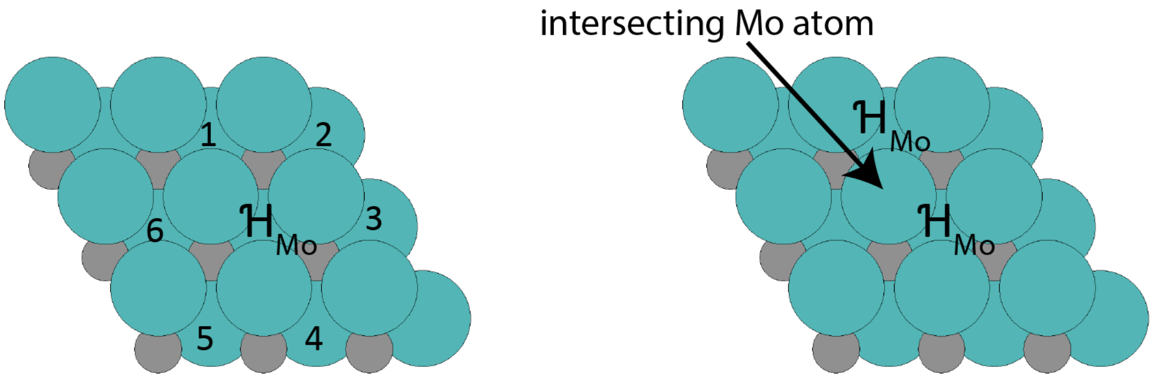

Figure S18. Vicinal three-fold hollow sites and intersecting Mo atom.

Left panel shows the six vicinal $\mathcal{H}_{Mo}$ sites around a central $\mathcal{H}_{Mo}$ site. The right panel shows how an intersecting Mo atom separates two vicinal $\mathcal{H}_{Mo}$ sites. The same terms are also used for the $\mathcal{H}_{C}$ sites.

Comparison of H_2-Assisted and Unassisted CO activation

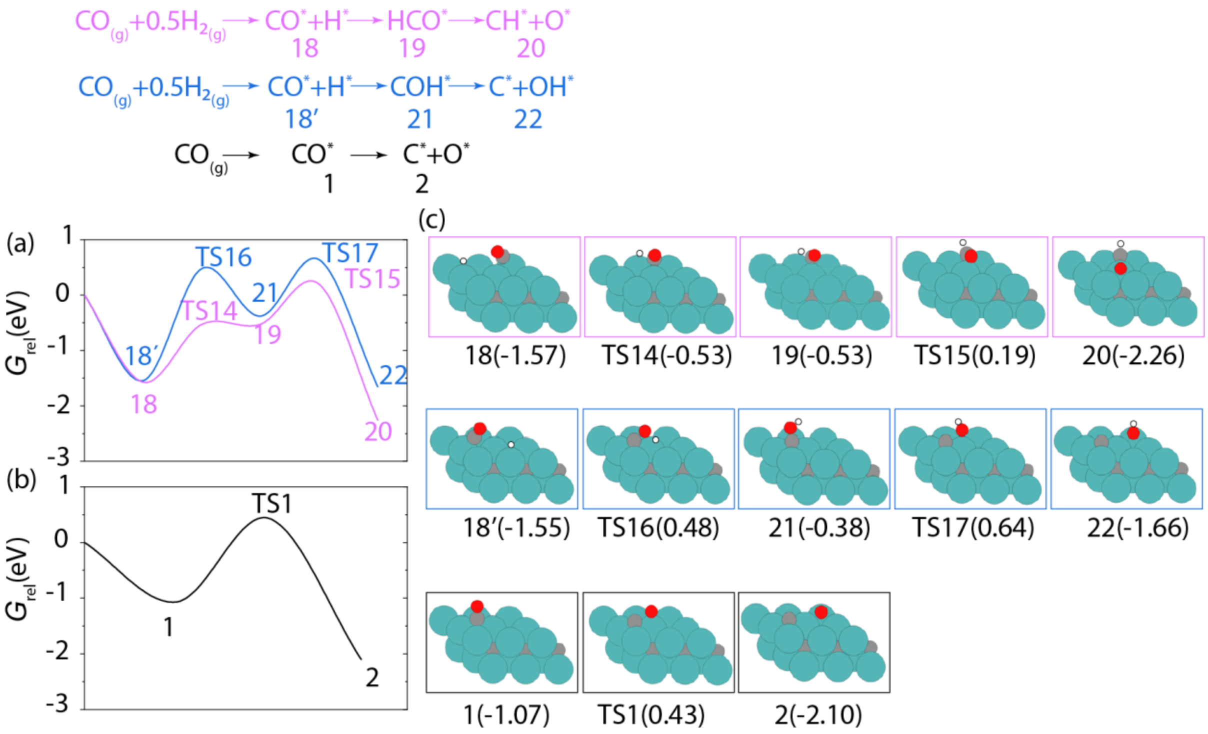

Figure S19. Energy profile for the (a) H_2-assisted and (b) direct CO dissociation pathways. Energies are calculated with respect to the initial reactants (a) 1 CO and 0.5 H_2 (b) 1 CO. Intermediate and transition states are shown in (c). The respective G_{rel} (eV) values are given in parenthesis.

The H_2-assisted activation routes involve the partial hydrogenation of CO^* to the formyl HCO^* species (**19**, Figure S19, magenta trace) or the hydroxyl carbonyl COH^* species (**21**, Figure S19 blue trace). These intermediates form via the addition of H^* to the C or O atoms of bound CO^*, respectively. For the formation of the hydroxyl carbonyl COH^* species to occur, the CO^* species should adopt a distorted μ_3-η^2 geometry (**18**). The transition state (TS14, −0.53 eV) involves migration of H^* from an $\mathcal{H}_{Mo}$ site atop of a neighboring Mo atom. In the product state **19**, both H and O atoms interact with Mo atoms leading to an μ_3–η^3 geometry. This elementary reaction is endergonic by 1.04 eV. Dissociation of the formyl species involves their rotation back to the μ_3–η^2 geometry, in which H interacts only with C via TS15 (0.19 eV), before the movement of O^* to a vicinal $\mathcal{H}_C$ site (intermediate **20**). The overall Gibbs energy difference between TS15 and **18** ($G_{TS15} - G_{18}$) is 1.77 eV. For the formation of the hydroxyl carbonyl, intermediate **18′** (notation prime indicates here a starting configuration as in **18**, but with a different geometry of the surface species) and product **21** states are stabilized in a μ_3-η^1 geometry, whereas an η^2 configuration appears in the transition state (TS16, 0.48 eV) such that the O becomes accessible for the bonding to the H^*. Dissociation of COH^* requires an η^2 configuration for the rupture of the C–O bond and the migration of the OH^* species to a vicinal $\mathcal{H}_C$ site (**22**). The process is exergonic by merely 0.11 eV and has a high overall Gibbs energy barrier (in this case, the energy difference between TS17 and **18′**) of 2.18 eV. The energy profile for the direct CO dissociation is shown in Figure S19 (black trace).

Water Formation

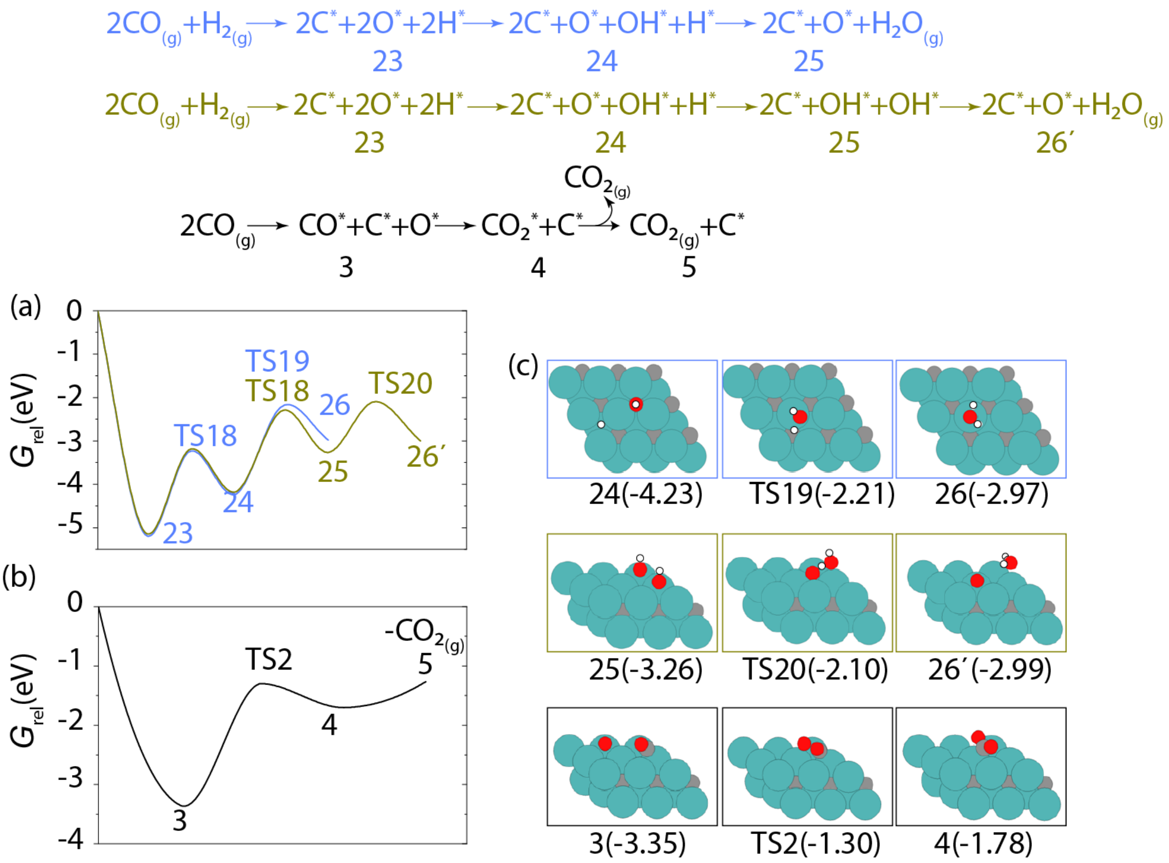

Figure S20. Energy profile for (a) water formation via OH hydrogenation and condensation of two OH sites, yielding H_2O^* and O^* (energies are calculated with respect to 2 CO and 1 H_2), and (b) CO_2 formation (energies are calculated with respect to 2 CO). Snapshots of the intermediate and transition states are shown in (c). The respective G_{rel} (eV) values are given in parenthesis.

Water can form either via a direct hydrogenation of a surface hydroxyl (Figure S20, blue trace) or via a hydrogen transfer between two hydroxyls (condensation of the neighboring hydroxyls, olive trace). For the formation of a hydroxyl, H^* and O^* occupy neighboring three-fold hollow sites ($\mathcal{H}_{Mo}$ and $\mathcal{H}_{C}$ respectively, intermediate **23**). Subsequently, to approach O^* species, H^* migrates on top of the intersecting Mo atom via a barrier of 1.87 eV (TS18). The direct hydrogenation of OH^* species requires the concurrent migration of OH^* and H^* from $\mathcal{H}_{C}$ and $\mathcal{H}_{Mo}$ respectively (**24**), atop a Mo atom (TS19) with an energy cost of 2.02 eV. Water interacts weakly on top of the Mo site (**26**), featuring an adsorption energy of merely 0.01 eV. The overall barrier for the removal of an O^* in the form of water via the direct hydrogenation of OH^* ($G_{TS19} – G_{23}$) is 2.56 eV. In the product state (**26**), two C^* and one O^* species are present on the surface in infinite distance from the formed water molecule (i.e., no interaction). The reverse barriers (dissociation of hydrogenated oxygen species), $OH^* \rightarrow O^* + H^*$ and $H_2O \rightarrow OH^* + H^*$ are 0.98 eV and 0.77 eV, respectively, indicating that the reverse reactions occur much faster than the forward reactions (assuming similar pre-exponential factors).

For the H transfer between two hydroxyls to occur, the two species need to occupy neighboring $\mathcal{H}_{C}$ sites (**25**), and, subsequently, migrate atop to two neighboring Mo atoms. In the transition state TS20, one hydrogen atom is shared between the O^* and OH^* species. After the H transfer, water migrates to an atop position while O^* migrates back to the $\mathcal{H}_{C}$ site (**26´**). The overall barrier for the removal of an O^*

in the form of water via proton transfer between the two OH^* species ($G_{TS20} - G_{23}$) is 2.66 eV. The reaction energy for the formation of a water molecule ($G_{26} - G_{23}$) is +2.15 eV.

Table S4. Activation barriers, reaction energies and reverse barriers of all elementary steps examined in the pathways of CO hydrogenation towards ethane on 2D-Mo_2C.

Entry	Elementary step	G_a (eV)	G_{rxn} (eV)	$G_{a,reverse}$ (eV)
R1	$CO^* \leftrightarrow C^*+O^*$	1.51	−1.02	2.53
R2	$CO^* + H^* \leftrightarrow COH^*$	2.02	1.17	0.86
R3	$COH^* \leftrightarrow C^*+ OH^*$	1.02	−1.28	2.29
R4	$CO^* + H^* \leftrightarrow HCO^*$	1.04	1.04	0.00
R5	$HCO^* \leftrightarrow HC^* + O^*$	0.72	−1.73	2.45
R6	$O^* + H^* \leftrightarrow OH^*$	1.87	0.89	0.98
R7	$OH^* + H^* \leftrightarrow H_2O^*$	2.02	1.25	0.77
R8	$2OH^* \leftrightarrow H_2O^*+O^*$	1.16	0.26	0.90
R9	$CO^* + O^* \leftrightarrow CO_2^*$	2.05	1.58	0.48
R10	$C^* + C^* \leftrightarrow CC^*$	1.43	−0.20	1.63
R11	$C^* + CH^* \leftrightarrow CCH^*$	1.20	−0.18	1.38
R12	$C^* + CH_2^* \leftrightarrow CCH_2^*$	1.53	0.36	1.17
R13	$C^* + CH_3^* \leftrightarrow CCH_3^*$	1.37	0.15	1.22
R14	$CH^* + CH^* \leftrightarrow CHCH^*$	0.85	−0.21	1.06
R15	$CH^* + CH_2^* \leftrightarrow CHCH_2^*$	1.30	0.48	0.82
R16	$CH^* + CH_3^* \leftrightarrow CHCH_3^*$	1.62	0.69	0.93
R17	$CH_2^* + CH_2^* \leftrightarrow CH_2CH_2^*$	1.35	0.30	1.05
R18	$CH_2^* + CH_3^* \leftrightarrow CH_2CH_3^*$	1.47	0.59	0.88
R19	$C^* + H^* \leftrightarrow CH^*$	0.90	0.17	0.74
R20	$CH^* + H^* \leftrightarrow CH_2^*$	0.71	0.56	0.15
R21	$CH_2^* + H^* \leftrightarrow CH_3^*$	0.80	0.35	0.45
R22	$CH_3^* + H^* \leftrightarrow CH_4^*$	1.57	0.70	0.87
R23	$CC^* + H^* \leftrightarrow CCH^*$	1.14	0.06	1.08
R24	$CCH^* + H^* \leftrightarrow CCH_2^*$	0.98	0.96	0.02
R25	$CCH_2^* + H^* \leftrightarrow CCH_3^*$	0.70	0.48	0.22
R26	$CHCH^* + H^* \leftrightarrow CHCH_2^*$	1.41	1.13	0.29
R27	$CCH^* + H^* \leftrightarrow CHCH^*$	1.14	0.07	1.06
R28	$CCH_2^* + H^* \leftrightarrow CHCH_2^*$	0.97	0.28	0.68
R29	$CCH_3^* + H^* \leftrightarrow CHCH_3^*$	0.75	0.41	0.34
R30	$CHCH_2^* + H^* \leftrightarrow CH_2CH_2^*$	0.75	0.51	0.24

R31	$CHCH_3^* + H^* \leftrightarrow CH_2CH_3^*$	0.81	0.52	0.29
R32	$CH_2CH_2^* + H^* \leftrightarrow CH_2CH_3^*$	0.76	0.53	0.23
R33	$CH_2CH_3^* + H^* \leftrightarrow CH_3CH_3^*$	1.28	0.48	0.80
R34	$CH_3CH_2CH_2^* + H^* \leftrightarrow CH_3CH_2CH_3^*$	1.39	0.57	0.81
R35	$CHCH^* + H^* \leftrightarrow CCH_3^*$	2.26	1.24	1.02

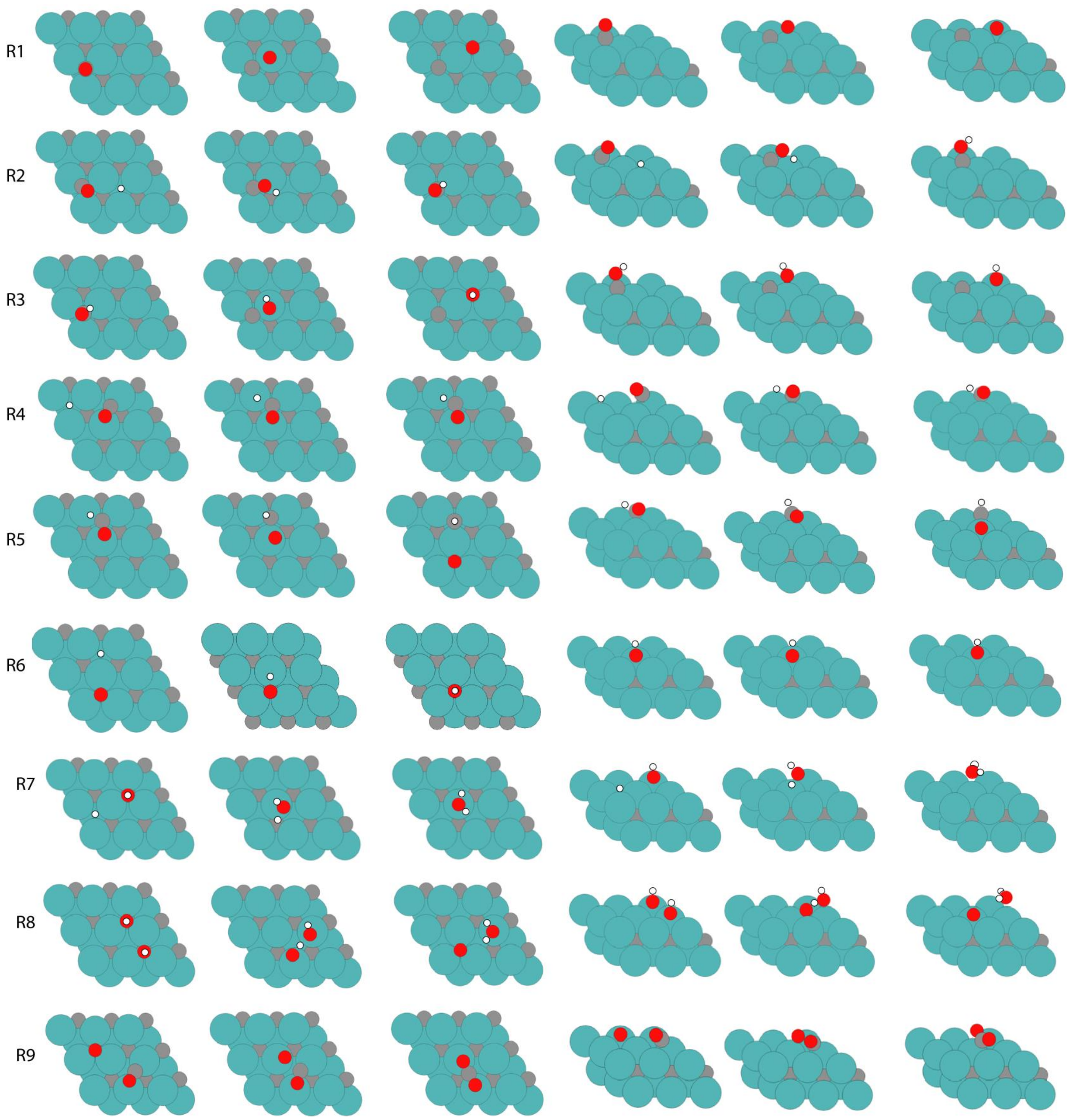

Figure S21. Top view (3 snapshots on the left) and side view (3 snapshots on the right) of initial (left), transition (middle), and final (right) states associated with steps R1-R9 presented in Table S4.

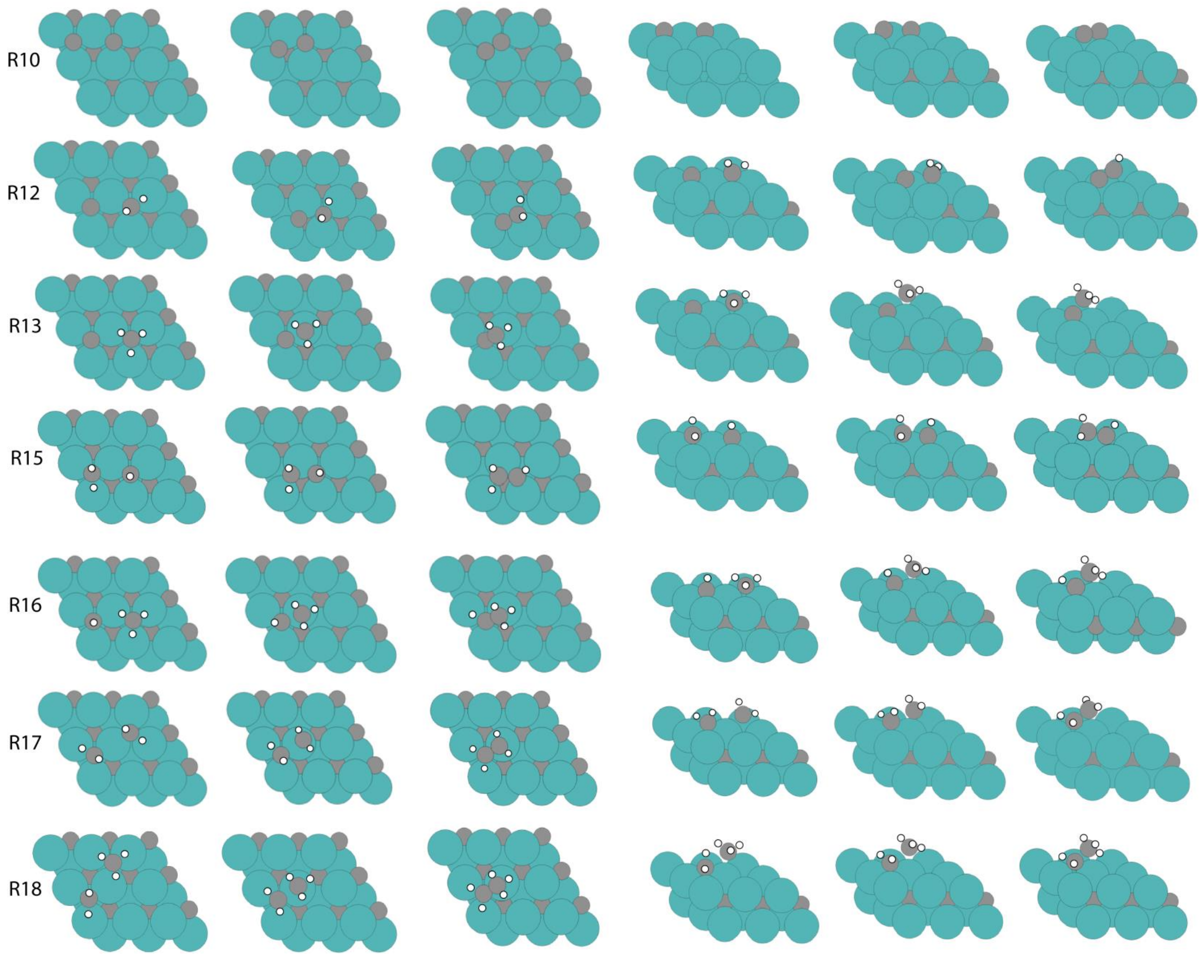

Figure S22. Top view (3 snapshots on the left) and side view (3 snapshots on the right) of initial (left), transition (middle), and final (right) states associated with steps R10-R18 presented in Table S4.

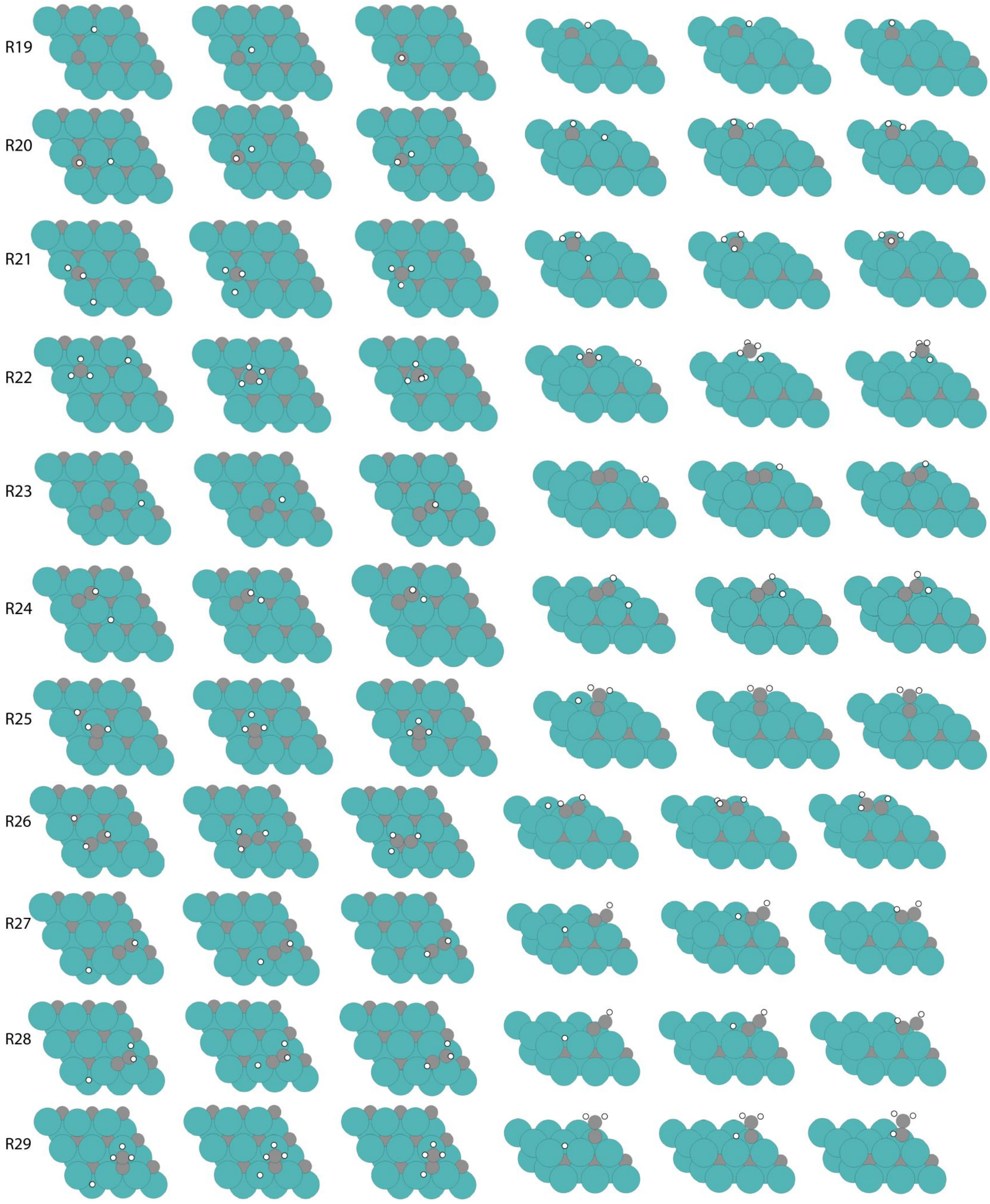

Figure S23. Top view (3 snapshots on the left) and side view (3 snapshots on the right) of initial (left), transition (middle), and final (right) states associated with steps R19-R29 presented in Table S4.

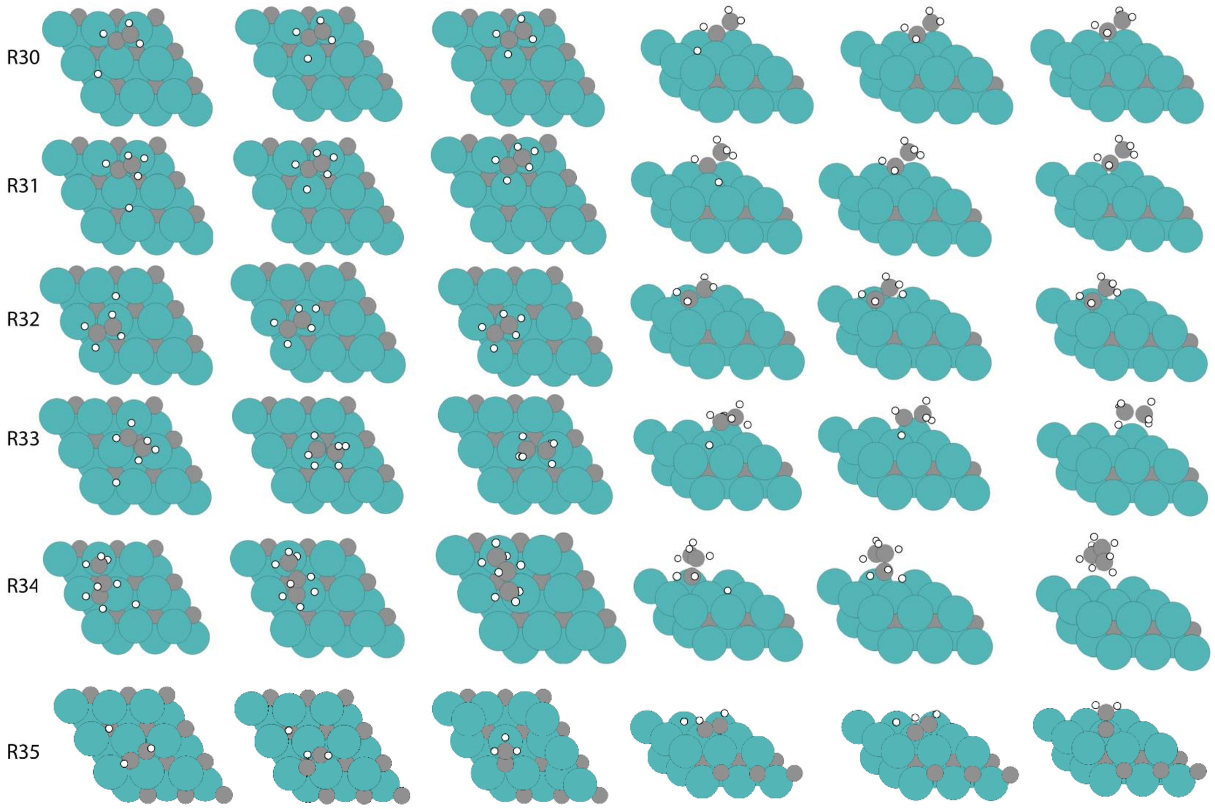

Figure S24. Top view (3 snapshots on the left) and side view (3 snapshots on the right) of initial (left), transition (middle), and final (right) states associated with steps R30-R35 presented in Table S4.

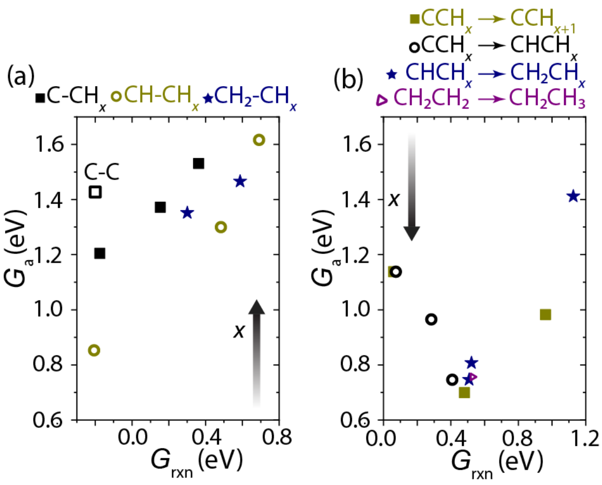

Figure S25. Gibbs energy barriers plotted against Gibbs reaction energies for (a) C–C coupling and (b) hydrogenation elementary steps (R1-R34). The arrow shows the direction of increase with increasing value of index *x*.

Alternative Pathways to Ethane

a) The Ethylene Pathway

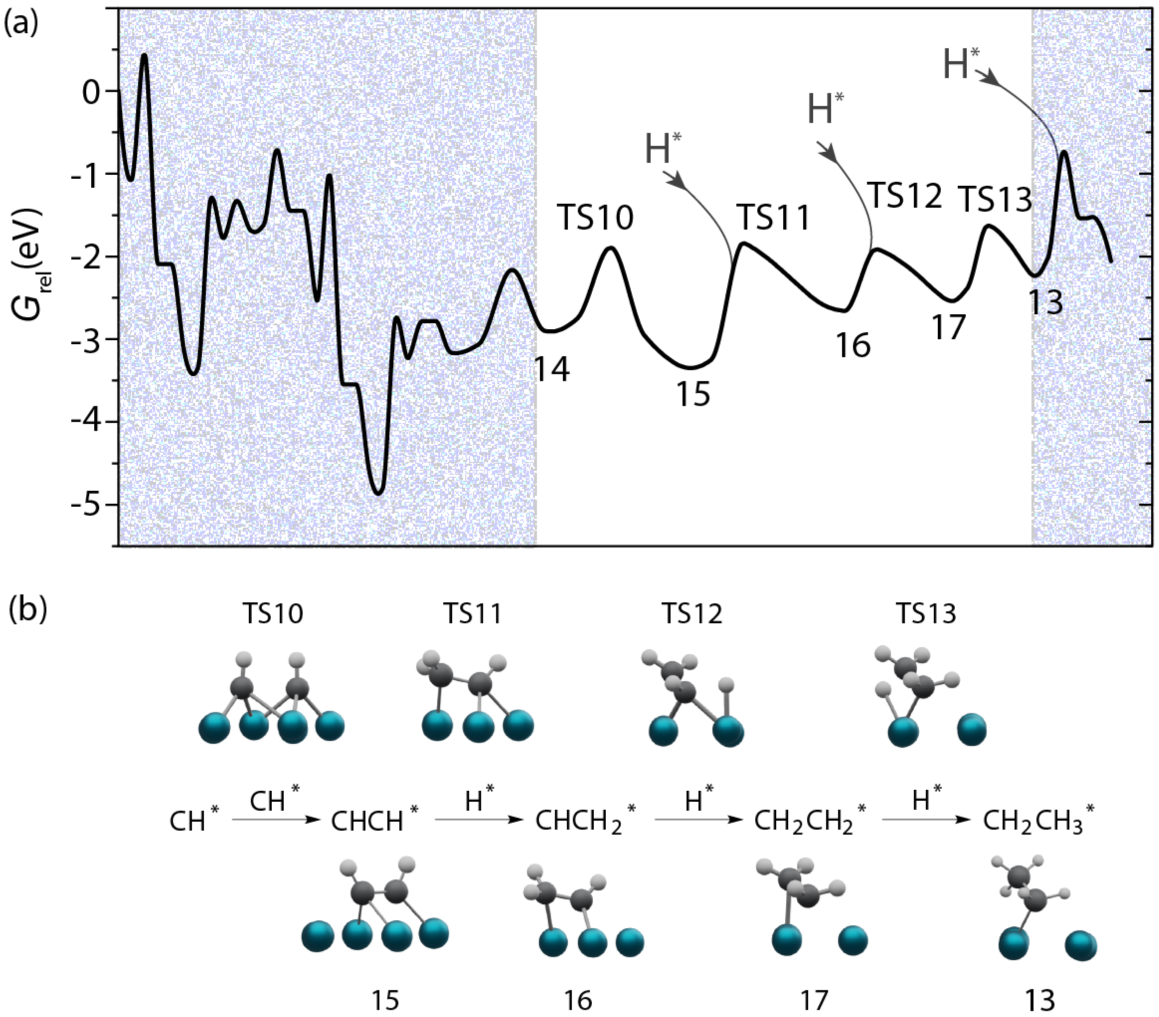

Figure S26. (a) Energy profile for ethane formation on a 2D-Mo_2C slab including CH–CH coupling steps via the formation of ethylene (black trace) as key intermediate. (b) Snapshots of selected intermediates and transition states.

The iris shade denotes steps of the main pathway described in Figure 4 of the main text.

b) H-Assisted Transformation of Acetylene to Ethylidyne

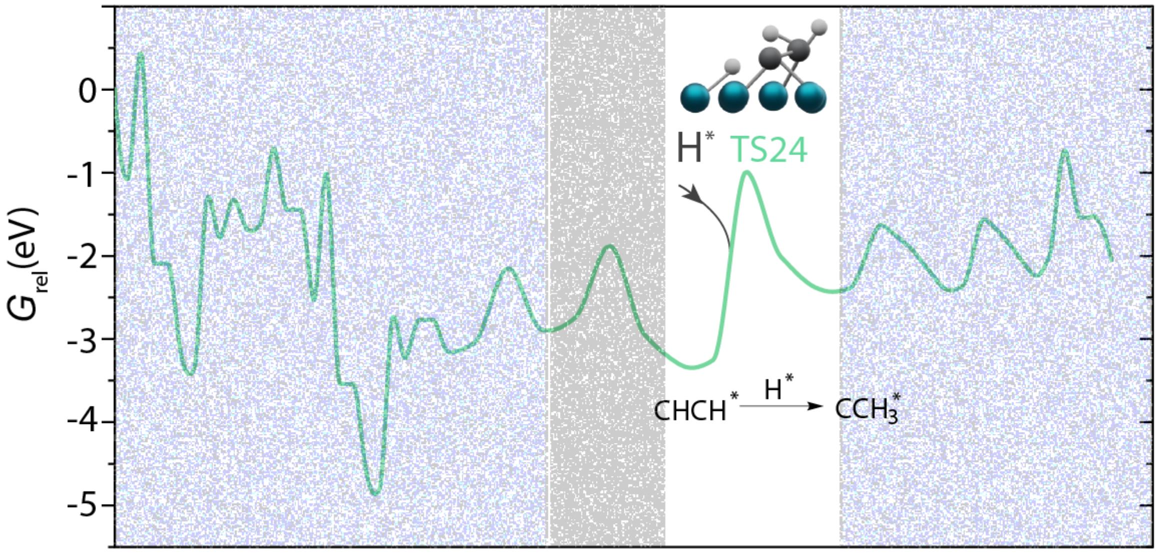

Figure S27. Energy profile for ethane formation on a 2D-Mo_2C slab including CH–CH coupling steps, via the H-assisted transformation of acetylene to ethylidyne.

The iris shade denotes steps of the main pathway described in Figure 4 of the main text. The gray shade denotes steps of the ethylene pathway described in Figure S26. A snapshot of the transition state is presented. Snapshots of the acetylene and ethylidyne are provided in Figure S26 and Figure 4c, respectively.

Methane Formation

The formation of methane presented in Figure S28 has been described in main text up to the intermediate state **7**. In this state, a CH^* species occupies a $\mathcal{H}_{Mo}$ site. Further hydrogenation requires an H^* species to occupy a vicinal $\mathcal{H}_{Mo}$ site. In the transition state TS21, H^* migrates atop of the intersecting Mo atom, a step associated with a barrier of 0.71 eV. Next, CH_2^* species (**27**) adopts an μ_3-η^2 geometry with only one of the hydrogen atoms interacting with the surface. Further hydrogenation requires that one more H^* from a vicinal $\mathcal{H}_{Mo}$ site migrates over the intersecting Mo atom via TS22 with a barrier of 0.80 eV. Formation of the new C–H bond occurs with a simultaneous rotation of the CH_2^* around the principal axis and increase of the H–C–H angle from ca. 98° to 105° for CH_3^* (**28**). For the final step, CH_3^* migrates from the $\mathcal{H}_{Mo}$ site atop to a neighboring Mo atom via TS23. This process requires a barrier of 1.57eV and it is endergonic by 0.70 eV. Once formed, release of the gas molecule of methane is exothermic by 0.35 eV.

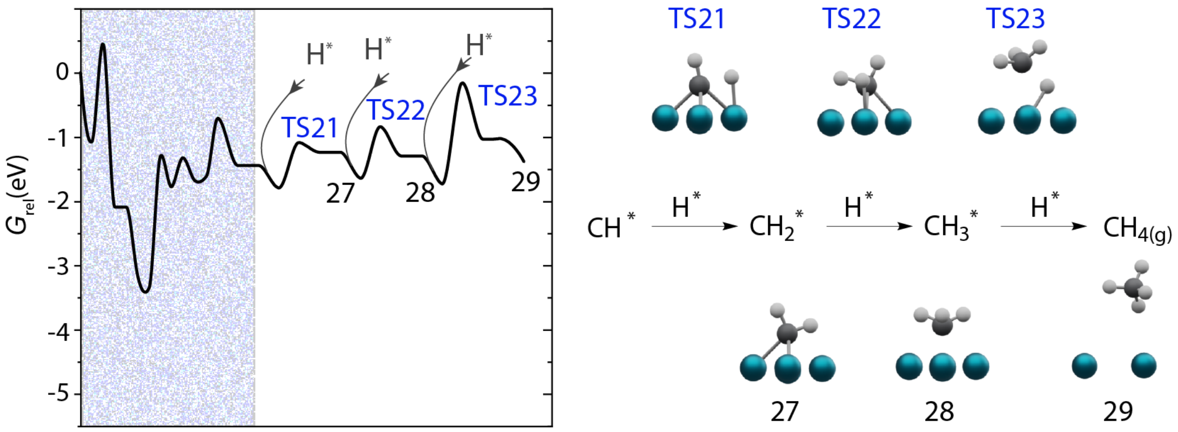

Figure S28. Energy profile for methane formation.

The iris shade denotes steps that are present in the main pathway described in Figure 4 of the main text.

Effect of O* species on the reaction energetics

$Mo_2CT_{x\text{-}400}$ has an average oxidation state of +3.3 and can be represented as 2D-Mo_2C with a 0.67 O^* monolayer coverage (ML), i.e., 2D-Mo_2C-0.67 O ML.[16] The 2D-Mo_2C-0.67 O ML model surface containing 6 O^* atoms per 9 Mo atoms, placed on $\mathcal{H}_C$ sites on the side of the reaction adsorbates, was considered to provide an explanation for the experimentally observed catalytic differences between $Mo_2CT_{x\text{–}400}$ prior to and after *in situ* activation. The reaction steps considered are: i) the dissociation of CO^*; ii) the removal of O^* as CO_2; iii) the coupling of C^* and CH^* species; iv) the hydrogenation of CH_3^* to methane. Activation barriers and reaction energies for the 2D-Mo_2C-0.67 O ML surface are presented in Table S5.

Table S5. Activation barriers, reaction energies and reverse barriers of selected elementary steps in the pathway of CO hydrogenation to ethane on the 2D-Mo_2C-0.67 O ML model.

Entry	Elementary step	G_a (eV)	G_{rxn} (eV)	$G_{a,reverse}$ (eV)
R1a	$CO^* \leftrightarrow C^*+O^*$	2.65	0.82	1.83
R9a	$CO^* + O^* \leftrightarrow CO_2^*$	0.75	0.72	0.03
R11a	$C^* + CH^* \leftrightarrow CCH^*$	1.12	−1.05	2.16
R22a	$CH_3^* + H^* \leftrightarrow CH_4^*$	0.96	−0.57	1.52

In the presence of 6 O^* surface species, corresponding to 0.67 O ML, the adsorption of CO at on-top sites becomes more favorable, despite the presence of remaining vacant $\mathcal{H}_{Mo}$ sites. The presence of 6 O^* adatoms results in a decreased CO adsorption energy, i.e., from −1.07 eV in 2D-Mo_2C to −0.46 eV in 2D-Mo_2C-0.67 O ML. The adsorption energy of CO_2 on 2D-Mo_2C-0.67 O ML is 0.79 eV (compared to −0.44 eV for 2D-Mo_2C). Figure S29 presents the Gibbs energy barriers and Gibbs reaction energies of the selected elementary steps mentioned above. 2D-Mo_2C-0.67 O ML features a low G_a for the oxygen removal step that produces CO_2, that is 0.75 eV, which is notably less than 2.05 eV for 2D-Mo_2C; the Gibbs reaction energy changes from strongly endergonic for 2D-Mo_2C (2.02 eV) to mildly exergonic for 2D-Mo_2C-0.67 O ML (−0.07 eV). Yet the dissociation of CO^* is hindered on 2D-Mo_2C-0.67 O ML relative to 2D-Mo_2C, i.e., the barrier for the CO^* dissociation increases from 1.51 eV for 2D-Mo_2C to 2.65 eV for 2D-Mo_2C-0.67 O ML. The reaction energy for the same elementary step changes from strongly exergonic for 2D-Mo_2C (−1.02 eV) to endergonic for 2D-Mo_2C-0.67 O ML (0.82 eV). The barrier for the coupling step is similar between 2D-Mo_2C and 2D-Mo_2C-0.67 O ML, i.e., 1.20 eV and 1.12 eV respectively, although the reaction is more exergonic in 2D-Mo_2C-0.67 O ML, with the respective Gibbs reaction energies of −0.18 eV and −1.05 eV, respectively. Finally, the hydrogenation of CH_3^* species towards methane is both kinetically easier (i.e., lower activation barriers) and thermodynamically more favorable on 2D-Mo_2C-0.67 O ML, showing a barrier of 0.96 eV and reaction energy −0.82 eV, compared to the barrier of 1.57 eV and reaction energy of 0.35 eV for 2D-Mo_2C. This result suggests that 2D-Mo_2C-0.67 O ML may show a higher selectivity to methane relative to 2D-Mo_2C, which is indeed observed experimentally (Table S1, entries 2-4). Overall, the lower activity of $Mo_2CT_{x\text{-}400}$ compared to $Mo_2CT_{x\text{-}400\text{-}SS}$ is, at least in part, due to the significantly higher barrier for the dissociation of CO on 2D-Mo_2C-0.67 O ML relative to 2D-Mo_2C.

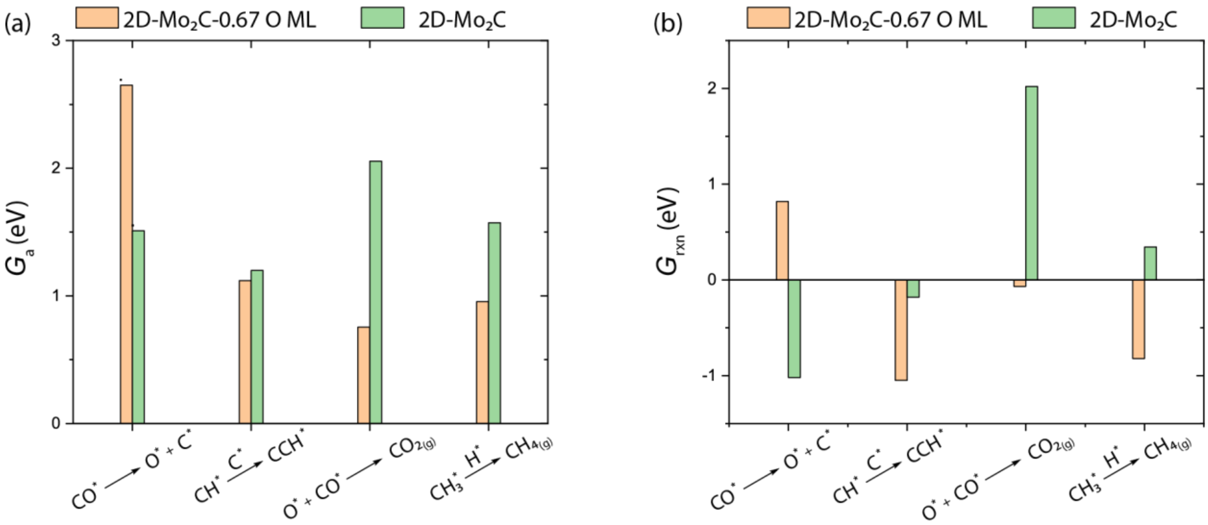

Figure S29. (a) Activation barriers and (b) reaction energies of selected elementary steps in the pathway of CO hydrogenation towards ethane on 2D-Mo_2C-0.67 O ML (orange bars) compared to 2D-Mo_2C (green bars).

Gibbs free energy correction

Polyatomic molecules are treated in the rigid rotator-harmonic oscillator approximation. The total energy is the sum of the electronic, translational, vibrational and rotational energies. The electronic contribution is derived from the periodic DFT calculations. The thermal contribution (translational, vibrational and rotational) in the Gibbs free energy of a given state is calculated as:[17, 18]

$$G_{thermal} = U + PV_{\mathrm{m}} - TS = U_{\mathrm{tran}} + U_{\mathrm{vib}} + U_{\mathrm{rot}} + PV_{\mathrm{m}} - T\left(S_{\mathrm{tran}} + S_{\mathrm{vib}} + S_{\mathrm{rot}}\right) \text{ (Eq. 1)}$$

The internal energy and the entropy are calculated from the partition function of an ideal polyatomic molecule, consisting of N atoms via equations Eq. 3.1- Eq. 3.3:

$$Q(N,V,T) = \frac{(q_{\mathrm{tran}} q_{\mathrm{rot}} q_{\mathrm{vib}} q_{\mathrm{elec}})^N}{N!} \text{ (Eq. 2)}$$

$$U_i = -\frac{\partial \ln q_i}{\partial \beta} \text{ (Eq. 3.1) } A_i = -\frac{\ln q_i}{\beta} \text{ (Eq. 3.2) } S_i = \frac{U_i - A_i}{T} \text{ (Eq. 3.3)}$$

The partition function for the translational mode is given by equation (Eq. 4)

$$q_{\mathrm{tran}} = \left(\frac{2\pi M k_{\mathrm{B}} T}{h^2}\right)^{3/2} V \text{ (Eq. 4)}$$

$$U_{\mathrm{tran}} = \frac{3}{2} N k_{\mathrm{B}} T \text{ (Eq. 5.1); } S_{\mathrm{tran}} = N k_{\mathrm{B}} \left(\frac{5}{2} + \frac{5}{2}\ln k_{\mathrm{B}} + \frac{5}{2}\ln T - \ln P + \frac{3}{2}\ln M + \frac{3}{2}\ln\left(\frac{2\pi}{h^2}\right)\right)$$

(Eq. 5.2)

The respective molar equations are:

$$U_{\text{tran,m}} = \frac{3}{2}RT \text{ (Eq. 6.1) and } S_{\text{tran,m}} = R\left(\frac{5}{2} + \frac{5}{2}\ln k_{\text{B}} + \frac{5}{2}\ln T - \ln P + \frac{3}{2}\ln\frac{\text{M}_{\text{r}}}{10^3 N_{\text{A}}} + \frac{3}{2}\ln\left(\frac{2\pi}{h^2}\right)\right)$$

(Eq. 6.2)

$$k_{\text{B}}[=]\frac{\text{J}}{\text{K}} \quad P[=]\frac{\text{N}}{\text{m}^2} \quad T[=]\text{K} \quad \text{M}_{\text{r}}[=]\frac{\text{g}}{\text{mol}}$$

The vibrational partition function is the product of functions for each normal vibrational mode:

$$q_{\text{vib}} = q_{\text{vib}_1} q_{\text{vib}_2} q_{\text{vib}_3} \cdots \text{(Eq. 7)}$$

For a linear polyatomic molecule, consisting of N atoms, there are $3N-5$ normal vibration modes, and $3N-6$ for a nonlinear molecule.

$$q_{\text{vib}} = \prod_i \frac{e^{-\frac{1}{2}\beta h\nu_i}}{1-e^{-\beta h\nu_i}} \text{ (Eq. 8)}$$

$$U_{\text{vib}} = \sum_i \frac{h\nu_i}{2} + \frac{h\nu_i e^{-\beta h\nu_i}}{1-e^{-\beta h\nu_i}} \text{ (Eq. 9.1)} \quad S_{\text{vib}} = k_{\text{B}} \sum_i \left\{ \frac{\frac{h\nu_i}{k_{\text{B}}T} e^{-\frac{h\nu_i}{k_{\text{B}}T}}}{1-e^{-\frac{h\nu_i}{k_{\text{B}}T}}} - \ln\left(1-e^{-\frac{h\nu_i}{k_{\text{B}}T}}\right) \right\} \text{ (Eq. 9.2)}$$

The respective molar equations are:

$$U_{\text{vib,m}} = \frac{1}{N_{\text{A}}} \sum_i \frac{h\nu_i}{2} + \frac{h\nu_i e^{-\beta h\nu_i}}{1-e^{-\beta h\nu_i}} \text{ (Eq. 10.1)} \quad S_{\text{vib}} = R \sum_i \left\{ \frac{\frac{h\nu_i}{k_{\text{B}}T} e^{-\frac{h\nu_i}{k_{\text{B}}T}}}{1-e^{-\frac{h\nu_i}{k_{\text{B}}T}}} - \ln\left(1-e^{-\frac{h\nu_i}{k_{\text{B}}T}}\right) \right\} \text{ (Eq. 10.2)}$$

Vibrational frequencies were obtained from optimized geometries by fixing the positions of the atoms in the slab. A vibrational energy cutoff of 50 cm^{-1} was used to treat the spurious imaginary frequency (or a positive frequency not exceeding 50 cm^{-1}).

The rotational partition for a linear molecule function is given by equation (Eq. 11)

$$q_{\text{rot}} = \sum_{j=0}^{\infty} (2J+1) \cdot e^{-\beta\varepsilon_J} \text{ (Eq. 11)}$$

where

$$\varepsilon_J = J(J+1)\frac{\hbar^2}{2I} \text{ (Eq. 12)}$$

and the degeneracy of rotational energy levels is given by the factor $(2J+1)$. Therefore:

$$q_{\text{rot}} = \frac{2I}{\beta\hbar^2} \text{ (Eq. 13)}$$

To account for the equivalent configurations in the phase space due to symmetry, the symmetry number σ is introduced:

$$q_{\text{rot}} = \frac{1}{\sigma}\frac{2I}{\beta\hbar^2} \text{ (Eq. 14)}$$

$\sigma = 1$ for asymmetric linear and $\sigma = 2$ for symmetric linear molecules.

I is the moment of inertia and for a molecule consisting of N atoms, it is given by equation (Eq. 15):

$$I = \sum_{j=1}^{N} m_j \left|\mathbf{r}_j - \mathbf{r}_{\text{cm}}\right|^2 \text{ (Eq. 15)}$$

where $\mathbf{r}_j = x_j\mathbf{x} + y_j\mathbf{y} + z_j\hat{\mathbf{z}}$ and $\mathbf{r}_{\text{cm}} = x_{\text{cm}}\mathbf{x} + y_{\text{cm}}\mathbf{y} + z_{\text{cm}}\hat{\mathbf{z}}$

$$x_{\text{cm}} = \frac{1}{M}\sum_{j=1}^{N} m_j x_j,\ y_{\text{cm}} = \frac{1}{M}\sum_{j=1}^{N} m_j y_j,\ z_{\text{cm}} = \frac{1}{M}\sum_{j=1}^{N} m_j z_j$$

Therefore

$$U_{\text{rot}} = k_{\text{B}}T \text{ (Eq. 16.1)} \quad S_{\text{rot}} = k_{\text{B}}\left(1 + \ln\left(\frac{k_{\text{B}}T}{\sigma\hbar^2}2I\right)\right) \text{ (Eq. 16.2)}$$

The respective molar values are:

$$U_{\text{rot,m}} = RT \text{ (Eq. 17.1)} \quad S_{\text{rot,m}} = R\left(1 + \ln\left(\frac{k_{\text{B}}T}{\sigma\hbar^2}2I\right)\right) \text{ (Eq. 17.2)}$$

For non-linear molecules $\varepsilon_J = J\left(J+1\right)\frac{\hbar^2}{2I}$ and the degeneracy of the energy levels is $\left(2J+1\right)^2$. The partition function depends on the principal moments of inertia.

- For a spherical top (rotor): $I_{\text{A}} = I_{\text{B}} = I_{\text{C}} = I$

$$q_{\text{rot}} = \frac{\sqrt{\pi}}{\sigma}\left(\frac{2I}{\beta\hbar^2}\right)^{3/2} \text{ (Eq. 18)}$$

- For a symmetric top: $I_{\text{A}} = I_{\text{B}} \neq I_{\text{C}}$

$$q_{\text{rot}} = \frac{\sqrt{\pi}}{\sigma}\left(\frac{2I_C}{\beta\hbar^2}\right)^{1/2}\left(\frac{2I_{A,B}}{\beta\hbar^2}\right)^{1/2} \text{ (Eq. 19)}$$

- For an asymmetric top: $I_{\text{A}} \neq I_{\text{B}} \neq I_{\text{C}}$

$$q_{\text{rot}} = \frac{\sqrt{\pi}}{\sigma}\left(\frac{2I_A}{\beta\hbar^2}\right)^{\frac{1}{2}}\left(\frac{2I_B}{\beta\hbar^2}\right)^{\frac{1}{2}}\left(\frac{2I_C}{\beta\hbar^2}\right)^{\frac{1}{2}} \text{ (Eq. 20)}$$

$$U_{\text{rot}} = \frac{3}{2}k_{\text{B}}T \text{ (Eq. 21.1)} \quad S_{\text{rot}} = k_{\text{B}}\left(\frac{3}{2} + \ln\left(\frac{\sqrt{\pi}}{\sigma}\right) + \ln\left[\left(\frac{8\pi^2 k_{\text{B}}T}{h^2}\right)^{\frac{3}{2}}\left(I_A I_B I_C\right)^{\frac{1}{2}}\right]\right) \text{ (Eq.21.2)}$$

$$U_{\text{rot,m}} = \frac{3}{2}RT \text{ (Eq. 22.1)} \quad S_{\text{rot,m}} = R\left(\frac{3}{2} + \ln\left(\frac{\sqrt{\pi}}{\sigma}\right) + \ln\left[\left(\frac{8\pi^2 k_{\text{B}}T}{h^2}\right)^{\frac{3}{2}}\left(I_A I_B I_C\right)^{\frac{1}{2}}\right]\right) \text{ (Eq. 22.2)}$$

To calculate the moments of inertia, the sdf files of the NIST database and the ABC Rotational Constant calculator where used (https://www.colby.edu/chemistry/PChem/scripts/ABC.html). For molecules adsorbed on the surface, the hindered translator/rotor model is used to calculate the thermal correction. In this model the term PV_{m} is considered negligible $U \approx H$. The rotational and vibrational part of the contribution is attributed to vibration ($3N$ degrees of freedom constitute the vibrational motion). Therefore, the thermal energy of species I adsorbed on the slab is given below:

$$G_{I/\text{slab,thermal}} = U_{\text{vib}} - TS_{\text{vib}} \text{ (Eq .23)}$$

All the energy values reported include the thermal correction.

The adsorption energy of a molecule I is calculated as follows:

$$G_{\text{ads},I} = \left(G_{I/\text{slab,elec}} - G_{slab,\text{elec}}\right) + G_{I/\text{slab,thermal}} - G_I \text{ (Eq. 24)}$$

Where $G_{I/\text{slab,elec}}$ is the electronic energy of the optimized slab with an adsorbed molecule I, $G_{slab,\text{elec}}$ is the electronic energy of the clean slab, $G_{I/\text{slab,thermal}}$ is the thermal energy of the adsorbed I calculated by Eq. 23 and G_I is the Gibbs free energy (electronic and thermal) of the same species, I, in the absence of the surface (empty unit cell) but at a constant PT ensemble.

The adsorption on three-fold hollow, bridge and on-top sites was examined for carbon, hydrogen, and oxygen. Adsorption on bridge sites was unstable for all atomic adsorbates. For oxygen and hydrogen, on-top sites were stable, but adsorption was less favorable than adsorption on a three-fold hollow site. For carbon, on-top sites were not stable. H and C adsorbed preferentially on $\mathcal{H}_{\text{Mo}}$ sites, whereas O over $\mathcal{H}_{\text{C}}$ sites. The adsorption energies and the details on the choice of reference energies are given in Table S2. $\mathcal{H}_{\text{C}}$ sites were used as an initial position for the relaxation of all OH_x adsorbates, whereas $\mathcal{H}_{\text{Mo}}$ sites was used to optimize all carbon containing species. To calculate the adsorption energies of atomic species we assume that molecular hydrogen, $H_{2(g)}$, is the source of atomic hydrogen and gas phase $CO_{(g)}$ is the source of C^* and O^* species. The Gibbs free energy of an adsorbed hydrogen atom (Eq. 25) is the sum of the electronic energy of the slab with the adsorbed atom on site j ($G_{\text{H}^*/\text{slab,elec}}[j]$), minus the electronic energy of the clean slab ($G_{\text{slab,elec}}$) plus the thermal correction ($G_{\text{H}^*/\text{slab,thermal}}[j]$). For the

products of CO dissociation, we calculate the adsorption energy (Eq. 26) for pairs of atomic species at different combinations of atomic sites ($\boldsymbol{j}$: $\mathcal{H}_{\text{Mo}}$, $\mathcal{H}_{\text{C}}$ and $\boldsymbol{k}$: $\mathcal{H}_{\text{Mo}}$, $\mathcal{H}_{\text{C}}$, on-top).

$$G_{\text{ads,H}^*}[j] = \left(G_{\text{H}^*/\text{slab,elec}}[j] - G_{\text{slab,elec}}\right) + G_{\text{H}^*/\text{slab,thermal}}[j] - \frac{1}{2}G_{\text{H}_{2(g)}} \quad \text{(Eq. 25)}$$

$$G_{\text{ads,C}^*,\text{O}^*}[j,k] = \left(G_{\text{C}^*/\text{slab,elec}}[j] - G_{\text{slab,elec}}\right) + G_{\text{C}^*/\text{slab,thermal}}[j] + \left(G_{\text{O}^*/\text{slab,elec}}[k] - G_{\text{slab,elec}}\right) + G_{\text{O}^*/\text{slab,thermal}}[k] - G_{\text{CO}_{(g)}}$$

(Eq. 26)

Table S6. Adsorption energy of atomic adsorbates C^*, O^*, H^* on $\mathcal{H}_{Mo}$, $\mathcal{H}_{C}$ and on-top sites.

$I[j]$	$G_{ads,I}$ (eV)
$H^*[\mathcal{H}_{Mo}]$	−0.51
$H^*[\mathcal{H}_{C}]$	−0.47
H^*[on-top]	−0.09
$C^*[\mathcal{H}_{Mo}]$ O^*[on-top]	−1.30
$C^*[\mathcal{H}_{Mo}]$ $O^*[\mathcal{H}_{Mo}]$	−2.10
$C^*[\mathcal{H}_{Mo}]$ $O^*[\mathcal{H}_{C}]$	−2.23
$C^*[\mathcal{H}_{C}]$ O^*[on-top]	−0.63
$C^*[\mathcal{H}_{C}]$ $O^*[\mathcal{H}_{Mo}]$	−1.42
$C^*[\mathcal{H}_{C}]$ $O^*[\mathcal{H}_{C}]$	−1.56

Table S7. XPS fitting parameters for fresh and activated (TOS = 2 h) catalysts for the Mo region.

Catalyst	State	Mo $3d_{5/2}$ B.E.	ΔB.E.	FWHM	Peak shape	Amount (%)
Mo_2CT_x	Mo^{4+}	229.5	3.3	1.4	LF[a]	55
	Mo^{5+}	232.4	3.2	2.7	GL[b]	45
Mo_2CT_{x-400}	Mo^{carb}	228.3	3.3	2.7	LF	38
	Mo^{4+}	229.1	3.2	1.6	LF	57
	Mo^{5+}	232.3	3.2	3.0	GL	5
Mo_2CT_{x-500}	Mo^{carb}	228.2	3.2	1.5	LF	100
$Mo_2CT_{x-400-TOS2h}$	Mo^{carb}	228.5	3.2	1.4	LF	41
	Mo^{4+}	229.2	3.2	1.1	LF	39
	Mo^{5+}	232.5	3.0	3.6	GL	20
$Mo_2CT_{x-500-TOS2h}$	Mo^{carb}	228.2	3.2	1.5	LF	100

[a]LF Lorentzian asymmetric lineshape with tail damping. [b]GL- Gaussian/Lorentzian line shape.

Table S8. XPS fitting parameters for fresh and activated (TOS = 2 h) catalysts in the C region.

Catalyst	State	C 1*s* B.E.	FWHM	Peak shape	Amount (%)
Mo_2CT_x	C^{carb}	283.4	1.5	LF[a]	34
	C–C	284.7	1.6	GL[b]	35
	C=O	285.9	2.3	GL	19
	O–C=O	288.5	3.0	GL	12
Mo_2CT_{x-400}	C^{carb}	283.0	1.8	LF	10
	C–C	284.9	2.2	GL	73
	C=O	286.0	2.2	GL	12
	O–C=O	288.3	2.3	GL	5
Mo_2CT_{x-500}	C^{carb}	283.2	1.8	LF	25
	C–C	284.8	2.3	GL	75
$Mo_2CT_{x-400-TOS2h}$	C^{carb}	283.1	1.5	LF	15
	C–C	284.8	1.6	GL	43
	C=O	285.9	2.2	GL	24
	O–C=O	288.1	1.6	GL	2
	CO^*	288.9	3.8	GL	16
$Mo_2CT_{x-500-TOS2h}$	C^{carb}	283.2	2.5	LF	15
	C–C	284.8	2.4	GL	85

[a]LF Lorentzian asymmetric lineshape with tail damping. [b]GL- Gaussian/Lorentzian line shape

The positions of the electronic states were constrained within ±0.2 eV for all analyzed materials (to account for the experimental error).[19] For each pair of the Mo $3d_{5/2}$ – $3d_{3/2}$ doublet in a spectrum, the FWHM was kept equal with a $3d_{5/2}/3d_{3/2}$ area ratio of 3/2. Mo^{+4} and Mo^{+2} states were fitted with asymmetric functions to account for the high density of states around the Fermi level, while the Mo^{+5} state was symmetric as the valence charge density is considerably lower for this state.[1] Under these fitting constrains, the FWHM and areas of each doublet were optimized for the best fit of the experimental data. All C 1*s* components were fitted with symmetric functions, except for the carbidic carbon at 283.2 eV that was fitted with an asymmetric function to account for the ionicity of the Mo–C bond.[2]

References

(1) Deeva, E. B.; Kurlov, A.; Abdala, P. M.; Lebedev, D.; Kim, S. M.; Gordon, C. P.; Tsoukalou, A.; Fedorov, A.; Müller, C. R. In Situ XANES/XRD Study of the Structural Stability of Two-Dimensional Molybdenum Carbide Mo_2CT_x: Implications for the Catalytic Activity in the Water–Gas Shift Reaction. *Chem. Mater.* **2019**, *31*, 4505-4513.
(2) Halim, J.; Kota, S.; Lukatskaya, M. R.; Naguib, M.; Zhao, M.-Q.; Moon, E. J.; Pitock, J.; Nanda, J.; May, S. J.; Gogotsi, Y.; et al. Synthesis and Characterization of 2D Molybdenum Carbide (MXene). *Adv. Funct. Mater.* **2016**, *26*, 3118-3127.
(3) Shirley, D. A. High-Resolution X-Ray Photoemission Spectrum of the Valence Bands of Gold. *Phys. Rev. B* **1972**, *5*, 4709-4714.
(4) Scofield, J. H. Hartree-Slater Subshell Photoionization Cross-Sections at 1254 and 1487 eV. *J. Electron Spectrosc. Relat. Phenom.* **1976**, *8*, 129-137.
(5) Blöchl, P. E. Projector Augmented-Wave Method. *Phys. Rev. B* **1994**, *50*, 17953-17979.
(6) Kresse, G.; Joubert, D. From Ultrasoft Pseudopotentials to the Projector Augmented-Wave Method. *Phys. Rev. B* **1999**, *59*, 1758-1775.
(7) Wellendorff, J.; Lundgaard, K. T.; Møgelhøj, A.; Petzold, V.; Landis, D. D.; Nørskov, J. K.; Bligaard, T.; Jacobsen, K. W. Density Functionals for Surface Science: Exchange-Correlation Model Development with Bayesian Error Estimation. *Phys. Rev. B* **2012**, *85*, 235149.
(8) Vosko, S. H.; Wilk, L.; Nusair, M. Accurate Spin-Dependent Electron Liquid Correlation Energies for Local Spin Density Calculations: a Critical Analysis. *Can. J. Phys.* **1980**, *58*, 1200-1211.
(9) Monkhorst, H. J.; Pack, J. D. Special Points for Brillouin-Zone Integrations. *Phys. Rev. B* **1976**, *13*, 5188-5192.
(10) Methfessel, M.; Paxton, A. T. High-Precision Sampling for Brillouin-Zone Integration in Metals. *Phys. Rev. B* **1989**, *40*, 3616-3621.
(11) Henkelman, G.; Jónsson, H. Improved Tangent Estimate in the Nudged Elastic Band Method for Finding Minimum Energy Paths and Saddle Points. *J. Chem. Phys.* **2000**, *113*, 9978-9985.
(12) Henkelman, G.; Uberuaga, B. P.; Jónsson, H. A Climbing Image Nudged Elastic Band Method for Finding Saddle Points and Minimum Energy Paths. *J. Chem. Phys.* **2000**, *113*, 9901-9904.
(13) Zhou, H.; Chen, Z.; Kountoupi, E.; Tsoukalou, A.; Abdala, P. M.; Florian, P.; Fedorov, A.; Müller, C. R. Two-Dimensional Molybdenum Carbide 2D-Mo_2C as a Superior Catalyst for CO_2 Hydrogenation. *Nat. Commun.* **2021**, *12*, 5510.
(14) Wu, Y.; Wang, L.; Chai, Z.; Shi, W. Heterostructure Engineering of MoS_2/Mo_2CT_x Nanoarray via Molten Salt Synthesis for Enhanced Hydrogen Evolution Reaction. *J. Mater.* **2023**, *9*, 1122-1128.
(15) Zhou, H.; Chen, Z.; López, A. V.; López, E. D.; Lam, E.; Tsoukalou, A.; Willinger, E.; Kuznetsov, D. A.; Mance, D.; Kierzkowska, A.; et al. Engineering the Cu/Mo_2CT_x (MXene) Interface to Drive CO_2 Hydrogenation to Methanol. *Nat. Catal.* **2021**, *4*, 860-871.
(16) Kurlov, A.; Deeva, E. B.; Abdala, P. M.; Lebedev, D.; Tsoukalou, A.; Comas-Vives, A.; Fedorov, A.; Müller, C. R. Exploiting Two-Dimensional Morphology of Molybdenum Oxycarbide to Enable Efficient Catalytic Dry Reforming of Methane. *Nat. Commun.* **2020**, *11*, 4920.
(17) McQuarrie, D. A. *Statistical Mechanics*; University Science Books, **2000**.
(18) McQuarrie, D. A.; Simon, J. D. *Physical Chemistry: A Molecular Approach*; University Science Books, **1997**.
(19) Gengenbach, T. R.; Major, G. H.; Linford, M. R.; Easton, C. D. Practical Guides for X-Ray Photoelectron Spectroscopy (XPS): Interpreting the Carbon 1s Spectrum. *J. Vac. Sci.* **2021**, *39*, 013204.
